# Proceedings of the International Cancer Imaging Society (ICIS) 18th Annual Teaching Course: Menton, France. 7-9 October 2018

**DOI:** 10.1186/s40644-018-0160-x

**Published:** 2018-09-03

## Faculty Abstracts

### Sunday 7th October – Morning Session: 9:00 – 10:30 Myeloma

#### A1 Potential of MRI

##### Christina Messiou^1,2^ (Christina.Messiou@rmh.nhs.uk)

###### ^1^Department of Radiology, The Royal Marsden NHS Foundation Trust, Fulham Rd, London, UK; ^2^Division of Radiotherapy and Imaging, The Institute of Cancer Research, 15 Cotswold Road, Sutton, UK

Whole body MRI is recognized as the most sensitive imaging test for the diagnosis of myeloma bone involvement and is recommended by the International Myeloma Working Group for work up of patients with asymptomatic myeloma or solitary plasmacytoma [1]. In some countries such as the United Kingdom, whole body MRI is now recommended as first line imaging in all patients with a suspected diagnosis of myeloma [2]. This is because abnormal signal on MRI has been shown to be associated with very high risk of asymptomatic myeloma progression with development of lytic bone lesions [3,4] and because treatment of patients with high risk asymptomatic disease confers a survival benefit [5].

Contemporary whole body MRI protocols, which incorporate diffusion weighted MRI, combine high sensitivity with quantitative response assessments, in addition to early detection of other features of myeloma such a vertebral collapse, fractures, spinal cord/nerve root progression and extramedullary disease [6].

The incremental net monetary benefit of whole body MRI and whole body CT are reportedly similar compared to a negative benefit reported for FDG PET/CT. Deterministic base case results reveal whole body MRI to have the largest rise in quality-adjusted life years of 0.0142 compared with 0.0119 for whole body CT and 0.0103 for FDG PET/CT [2].

The non-invasive nature of whole body MRI facilitates longitudinal assessments which are particularly important for monitoring patients with non-secretory disease, oligosecretory disease and extramedullary disease. It is important to recognise that patients with secretory disease may become oligosecretory at relapse [7] and therefore as patients with myeloma live longer and undergo multiple lines of treatment, follow up imaging will gain in importance. Whole body MRI can also be used to direct biopsy or to judge the potential of sampling error from posterior iliac crest trephine.

The capability of whole body MRI to demonstrate both focal and diffuse marrow infiltration throughout the whole skeleton makes this extremely promising as a subjective tool for monitoring disease status and assessment of response. For focal lesions changes in size and number can be easily assessed and for diffuse infiltration signal changes are evident.

However, diffusion weighted MRI offers the capability to quantify disease which is particularly relevant as changes in Apparent Diffusion Coefficient (ADC) predate changes in size of focal deposits [8,9] and dimension based assessments are not applicable to diffuse infiltration. Reassuringly, reproducibility of ADC in the marrow have been reported as low as 2.8% and ADC changes have successfully separated responders from non-responders as well as indicating depth of response [10,11]. Although the literature on imaging Minimal Residual Disease is currently dominated by FDG PET/CT studies, conventional whole body MRI in patients before and after autologous stem cell transplantation (ASCT) treatment has also been shown to correlate with and predict overall survival [12] and studies of diffusion weighted MRI in this setting are underway (ClinicalTrials.gov NCT02403102). It is likely that imaging measures of minimal residual disease will be complimentary to serum and marrow estimates [12,13] in guiding therapeutic decisions.

1. Dimopoulos M, Hillengass J, Usmani, S et al. Role of Magnetic Resonance Imaging in the Management of Patients with Multiple Myeloma: A Consensus Statement. Journal of Clinical Oncology 2015; 6: 657-664.
2. Myeloma Diagnosis and Management. NICE (NG35). https://www.nice.org.uk/guidance/ng35
3. Kastritis E., Terpos E., Moulopoulos L et al. Extensive bone marrow infiltration and abnormal free light chain ratio identifies patients with asymptomatic myeloma at high risk for progression to symptomatic disease. Leukemia 2013; 27: 947-953.
4. Hillengass J, Fechtner K, Weber MA et al. Prognostic significance of focal lesions in whole-body magnetic resonance imaging in patients with asymptomatic multiple myeloma. JCO 2010;28:1606-10.
5. Mateos M, Hernandez M, Giraldo P. Lenalidomide plus Dexamethasone for High-Risk Smoldering Multiple Myeloma. The New England Journal of Medicine 2013; 369: 438-447.
6. Messiou C and Kaiser M. Whole body diffusion weighted MRI – a new view of myeloma. BJH 2015; 171: 29-37.
7. Chantry A, et al. British Society for Haematology. Guidelines for the use of imaging in the management of patients with myeloma. Br J Haematol. 2017 Jul 5. [Epub] PMID: 28677897
8. Messiou C, Collins D, Giles S et al. Assessing response in prostate bone metastases with diffusion weighted MRI. Eur Radiology 2011; 21:2169-77.
9. Latifoltojar A, Hall-Craggs M, Rabin N et al. Whole body magnetic resonance imaging in newly diagnosed multiple myeloma: early changes in lesional signal fat fraction predict disease response. BJH 2017; 176: 222-233.
10. Giles S, Messiou C, Collins D et al. Whole-body diffusion-weighted MR imaging for assessment of treatment response in myeloma. Radiology 2014;271:785-94.
11. Wu C, Huang J, Xu W et al. Discriminating depth of response to therapy in multiple myeloma using whole body diffusion weighted MRI with apparent diffusion coefficient.
12. Hillengass J, Ayyaz S, Kilk K et al. Changes in magnetic resonance imaging before and after autologous stem cell transplantation correlate with response and survival in multiple myeloma. Haematologica 2012; 97: 1757-1760.
13. Moreau P, Attal M, Caillot D et al. Prospective Evaluation of Magnetic Resonance Imaging and [18F]Fluorodeoxyglucose Positron Emission Tomography-Computed Tomography at Diagnosis and Before Maintenance Therapy in Symptomatic Patients With Multiple Myeloma Included in the IFM/DFCI 2009 Trial: Results of the IMAJEM Study. JCO 2017; 35(25):2911-2918.

#### A2 The Potential of PET/CT in Myeloma

##### Rodney J. Hicks

###### The Sir Peter MacCallum Department of Oncology the University of Melbourne, Parkville, Australia

Current consensus guidelines recommend the use of either whole-body MRI or FDG PET/CT in the primary evaluation of newly diagnosed myeloma or solitary plasmacytoma depending on local availability. Most comparisons of these techniques suggest that MRI finds more sites of apparent disease but the value of these extra findings is diminished by a higher false-positive rate and poorer ability to stratify prognosis than found with FDG PET/CT. Similarly, FDG PET/CT is generally favoured for therapeutic response assessment because of its strong ability to stratify prognosis but MRI have also been advocated, particularly with newer techniques. Nevertheless, both techniques have limitations and therefore might be complementary if performed as a single PET/MRI investigation. The major disadvantage of this technology is the loss of the detailed evaluation of bone architecture that is provided by the CT component of PET/CT as lytic bone disease has bone prognostic and therapeutic implications. While false-positive results constrain the utility of MRI, false-negative results are the major limitation of FDG PET/CT but false-positive results due to granulomatous disease, infection or other cancers also limit overall accuracy. Accordingly, there have been efforts to develop alternative molecular imaging agents to better characterize disease. Because the production of para-proteins requires a supply of amino-acids, C-11 methionine PET/CT has been investigated and appears to be at least as sensitive as FDG PET/CT and possibly more specific. Although the LAT1 amino-acid transporter has been found to be upregulated in myeloma, F-18 fluoro-ethyl-tyrosine (FET) PET/CT has generally been disappointing relative to FDG. Other LAT1 ligands, including radiolabeled phenylalanine analogues may be superior in this regard. Other cellular targets include proliferation markers, specific antigens and receptors that modulate cell behaviour. F-18 fluorocholine (FCH), which traces cell membrane production, looks promising but F-18 fluoro-L-thymidine (FLT) has limited capacity for assessment of the marrow due to high uptake in normal proliferating marrow. Radiolabeled analogues of the therapeutic anti-CD38 daratumumab also show promise while the agents that target the CXCR4 receptor seem to offer the most immediate prospect as theranostic agents with Ga-68 pentixafor having impressive sensitivity and specificity and providing the ability to select patients who might benefit from the use of therapeutic Lu-177 or Y-90 pentixafor. Thus, while FDG PET/CT and MRI both provide the ability to stage disease and potentially provide prognostic stratification, the potential of PET/CT for the future probably lies in the use of more specific diagnostic techniques that help to define optimal treatment pathways. This is important, particularly in refractory myeloma, which has an extremely bleak prognosis, where new therapeutic options are sorely needed.

**References**

1. Moreau, P., et al., Multiple myeloma: ESMO Clinical Practice Guidelines for diagnosis, treatment and follow-up. *Annals of Oncology*, 2017. 28(suppl_4): iv52-iv61.

2. Cavo, M., et al., Role of (18)F-FDG PET/CT in the diagnosis and management of multiple myeloma and other plasma cell disorders: a consensus statement by the International Myeloma Working Group. *Lancet Oncology*, 2017. 18(4): e206-e217.

3. Lapa, C., et al., (11)C-Methionine-PET in Multiple Myeloma: A Combined Study from Two Different Institutions. *Theranostics*, 2017. 7(11): 2956-2964.

4. Cassou-Mounat, T., et al., 18F-fluorocholine versus 18F-fluorodeoxyglucose for PET/CT imaging in patients with suspected relapsing or progressive multiple myeloma: a pilot study. *European Journal of Nuclear Medicine and Molecular Imaging*, 2016. 43(11): 1995-2004.

5. Sachpekidis, C., et al., Assessment of glucose metabolism and cellular proliferation in multiple myeloma: a first report on combined (18)F-FDG and (18)F-FLT PET/CT imaging. *EJNMMI Research*, 2018. 8(1): 28.

6. Caserta, E., et al., Copper 64-labeled daratumumab as a PET/CT imaging tracer for multiple myeloma. *Blood*, 2018. 131(7): 741-745.

7. Philipp-Abbrederis, K., et al., In vivo molecular imaging of chemokine receptor CXCR4 expression in patients with advanced multiple myeloma. *EMBO Molecular Medicine*, 2015. 7(4): 477-87.

8. Lapa, C., et al., [(68)Ga]Pentixafor-PET/CT for imaging of chemokine receptor CXCR4 expression in multiple myeloma - Comparison to [(18)F]FDG and laboratory values. *Theranostics*, 2017. 7(1): 205-212.

9. Herrmann, K., et al., First-in-Human Experience of CXCR4-Directed Endoradiotherapy with 177Lu- and 90Y-Labeled Pentixather in Advanced-Stage Multiple Myeloma with Extensive Intra- and Extramedullary Disease. *Journal of Nuclear Medicine*, 2016. 57(2): p. 248-51.

10. Lapa, C., et al., CXCR4-directed endoradiotherapy induces high response rates in extramedullary relapsed Multiple Myeloma. *Theranostics*, 2017. 7(6): 1589-1597.

### 11:00 – 12:30 Lymphoma and Myelofibrosis

#### A3 Utility of PET/CT for imaging bone marrow in lymphoma

##### Sally F Barrington

###### KCL and Guy’s and St Thomas’ PET Centre, School of Biomedical Engineering and Imaging Sciences, Kings College London, London, UK

**Staging**

The utility of Positron Emission Tomography (PET) for staging in lymphomas relates to the pattern of marrow involvement, with high sensitivity where there is predominantly focal involvement of the marrow, including Hodgkin and Large B Cell Lymphomas^1^. In these subtypes, PET-CT is very sensitive and more sensitive than the bone marrow biopsy (BMB) which is taken from the iliac crest and will miss sites of focal disease elsewhere in the marrow.

Approximately 1% of patients with Hodgkin lymphoma (HL) are upstaged by BMB compared to PET imaging^2^, but this has no impact on management^3^ and PET has replaced the BMB at diagnosis^4^. In diffuse large B cell lymphoma (DLBCL) PET has high sensitivity for large cell lymphoma in the marrow and it is uncommon to miss clinically significant marrow disease^5^. ‘Discordant’ small cell disease, with a mixture of large cells in the lymph nodes and small cells in the marrow may be missed on PET imaging^5^, however this does not affect prognosis. There is no evidence that altering treatment or closer follow up for patients with discordant disease in the marrow affects outcome, however practice varies as to whether routine BMB is still performed as part of routine staging.

Marrow involvement with indolent lymphomas including many follicular lymphomas where there is diffuse, often low grade involvement of the marrow is not well imaged using PET-CT and bone marrow biopsy remains a better test to stage the bone marrow^6^.

**Response assessment**

Diffuse uptake frequently occurs in the marrow related to chemotherapy and following administration of myeloid colony stimulating factors, which reporters of PET should recognise and not misinterpret as lymphomatous involvement^7^. Ablation of diseased marrow can occur after successful treatment with photopaenia at these sites. Stimulation of normal adjacent marrow may lead to increased uptake, whereby the uptake in the marrow at response looks like a ‘mirror image’ of the baseline scan. Correct interpretation of serial scans in this situation relies on having a baseline scan for comparison. Complete metabolic response may be inferred if uptake at sites of initial involvement in the marrow is no greater than surrounding normal marrow even if higher than normal liver uptake using the Deauville criteria^8^.

Response in the marrow should be viewed in the context of response in the nodes. If there are persistent focal changes in the marrow in the context of a nodal response, consideration should be given to further evaluation with MRI, biopsy or an interval scan^8^, as increased marrow uptake may persist where there is active healing/inflammation.

**References**

1. Barrington SF, Mikhaeel NG, Kostakoglu L, et al. Role of imaging in the staging and response assessment of lymphoma: Consensus of the international conference on malignant lymphomas imaging working group. *J Clin Oncol*. 2014;32(27):3048-3058.

2. Barrington SF, Kirkwood AA, Franceschetto A, et al. PET-CT for staging and early response: Results from the response-adapted therapy in advanced Hodgkin lymphoma study. *Blood*. 2016;127(12):1531-1538.

3. El-Galaly TC, d'Amore F, Mylam KJ, et al. Routine bone marrow biopsy has little or no therapeutic consequence for positron emission tomography/computed tomography-staged treatment-naive patients with hodgkin lymphoma. *J Clin Oncol*. 2012;30(36):4508-4514.

4. Cheson BD. Hodgkin lymphoma: Protecting the victims of our success. *J Clin Oncol*. 2012;30(36):4456-4457.

5. Alzahrani M, El-Galaly TC, Hutchings M, et al. The value of routine bone marrow biopsy in patients with diffuse large B-cell lymphoma staged with PET/CT: A danish-canadian study. *Ann Oncol*. 2016;27(6):1095-1099.

6. Luminari S, Biasoli I, Arcaini L, et al. The use of FDG-PET in the initial staging of 142 patients with follicular lymphoma: A retrospective study from the FOLL05 randomized trial of the fondazione italiana linfomi. *Ann Oncol*. 2013;24(8):2108-2112.

7. Zwarthoed C, El-Galaly TC, Canepari M, et al. Prognostic value of bone marrow tracer uptake pattern in baseline PET scans in Hodgkin lymphoma: Results from an international collaborative study. *J Nucl Med*. 2017;58(8):1249-1254.

8. Cheson BD, Fisher RI, Barrington SF, et al. Recommendations for initial evaluation, staging, and response assessment of Hodgkin and non-Hodgkin lymphoma: The Lugano classification. *J Clin Oncol*. 2014;32(27):3059-3068.

#### A4 What is expected from imaging in myelofibrosis

##### Stéphane Giraudier^1,2^, Laetitia Vercellino^1^

###### ^1^Saint Louis Hospital Assistance Publique Hôpitaux de Paris, Paris, France; ^2^Paris Diderot University, Paris, France

Myelofibrosis (MF) is a histological term associated to the development of high density collagen and reticulin fiber depositions in the hematopoietic bone marrow. This histological feature can be observed in numerous disorders from tuberculosis to hematologic disorders such as myelodysplastic syndromes, leukemias and lymphomas. We usually call MF two entities: Primitive Myelofibrosis (PMF) and secondary myelofibrosis (post Essential thrombocytemia (PostET-MF) and polycythemia vera (Post-PV-MF)) that are myeloproliferative neoplasms (MPN). The other pathologies are associated to myelofibrosis but the fibrosis development is usually milder than what is observed in PMF and PET-MF or PPV-MF.

**Myelofibrosis: pathophysiology, Clinical findings and therapeutics**

PMF is classified broadly as a chronic myeloproliferative disorder. The main cause of the disorder is the acquisition in the hematopoietic stem cell compartment of mutations in proliferative-associated transduction pathways such as JAK2, CALR or Thrombopoietin-receptor (MPL) genes. These mutations are at least in part responsible for the disease initiation. Secondary molecular events are also frequent in PMF. Whatever the mutational status, the driver mutations are responsible for megakaryocytic hyperplasia, ineffective erythropoiesis, dysplastic megakaryocytes, and an increase in the ratio of immature granulocytes to total granulocytes. The fibrosis development is considered to be reactive to this hematopoietic disorder and is associated to abnormal cytokine releases (TGFb, FGF, VEGF..) by the hematopoietic cells. These releases are also considered as responsible for the constitutional symptoms observed in this disorder (fever, weight loss, night sweats, pruritus…). The impaired bone marrow hematopoiesis is associated to a shift of blood production to spleen and liver, and to spleen and liver enlargement. This disorder is the most severe myeloproliferative disorder with a median life expectancy reduced to 4 years after diagnosis and varying from 6 months to 10 years). Complications associate thrombo-hemorrhagic events and transformations in acute myeloid leukemias.

Current treatments are palliative and bone marrow transplantation (BMT) is the only curative approach. Unfortunately, BMT can be proposed only to young patients (median age at diagnosis of PMF is 60 years) without comorbidities. Prognosis classifications have been proposed in PMF and PPV-MF and PET-MF to delineate indications of such therapy and only patients with life expectancy lower than 3 years are considered as BMT-eligible.

Other palliative treatments consist in anti-JAK2, Hydroxyurea or Interferon and supportive therapies just like Erythropoietin. Despite palliative, these treatments can increase the life expectancy.

**Myelofibrosis: The Unsolved Questions concerning imaging**

Imaging in MF could be relevant in three main aspects i: diagnosis, complications and response to therapies. 1- MPN classification has been recently modified to include the misdiagnosed essential thrombocytemia (ET) patients who present mild myelofibrosis. Because differentiating ET and PMF impact survival, histological analysis is now recommended in ET. Could imaging differentiate ET and smoldering PMF in order to limit the biopsy for ET patients? The second aspect concerning diagnosis is about the quantification of fibrosis and its homogeneity in the bones. It is not yet clear if quantification of collagen and reticulin amounts in the bone marrow are correlated to prognosis. Could imaging answer this question?

2- About the diagnosis of PMF complications and more particularly extramedullary hematopoiesis (EMH) that is a specific complication of this disorder. It is well known that such extramedullary hematopoiesis can involve spleen, liver, lymph nodes, lung, bones, pleural localization are also observed… What is the best way to diagnose such complications?

3- Since new therapies emerge and seem to be more efficient than the ones previously prescribed, fibrosis quantification could be relevant as a marker of response to therapies. What is the kinetic of response of fibrosis and hematopoietic pathogenic clone?

**References**

1. **Myelofibrosis**: clinicopathologic features, prognosis, and management.O'Sullivan JM, Harrison CN. Clin Adv Hematol Oncol. 2018 Feb;16(2):121-131

2. Philadelphia chromosome-negative classical myeloproliferative neoplasms: revised management recommendations from European LeukemiaNet. Barbui T, Tefferi A, Vannucchi AM, Passamonti F, Silver RT, Hoffman R, Verstovsek S, Mesa R, Kiladjian JJ, Hehlmann R, Reiter A, Cervantes F, Harrison C, Mc Mullin MF, Hasselbalch HC, Koschmieder S, Marchetti M, Bacigalupo A, Finazzi G, Kroeger N, Griesshammer M, Birgegard G, Barosi G. Leukemia. 2018 May;32(5):1057-1069.

3. **Imaging** findings in patients with **myelofibrosis**.**Guermazi** A, de Kerviler E, Cazals-Hatem D, Zagdanski AM, Frija J. Eur Radiol. 1999;9(7):1366-75.

#### A5 PET in myelofibrosis

##### Laetitia Vercellino^1^, Stéphane Giraudier^1,2^

###### ^1^Saint Louis Hospital Assistance Publique Hôpitaux de Paris, Paris, France; ^2^Paris Diderot University, Paris, France

Imaging bone marrow in myelofibrosis (MF) can be useful for initial staging of the disease, for therapy monitoring and adjustment. Bone marrow (BM) scintigraphy has long been used in several hematological diseases. It allows qualitative assessment of uptake in axial skeleton, as well as identification of extramedullary hematopoiesis, especially myeloid metaplasia of the spleen. Positron emission tomography (PET), with its enhanced imaging quality, and quantitative analysis possibilities appears promising in this clinical context. Two PET tracers have especially been studied to explore MF, 18F-Fluorodeoxyglucose (18F-FDG) and 18F-Fluoro-L-thymidine (18F-FLT).

**18F-FDG PET in myelofibrosis**

In 30 patients assessed with FDG PET before stem cell transplantation (SCT), Derlin et al. analyzed the distribution of FDG uptake in bone marrow and the spleen, resulting from the inflammation present in MF [1]. They described four patterns of FDG uptake: from grade 1, with a mildly increased uptake in the central skeleton and the proximal extremities, to grade 4 with an increased uptake in the central skeleton and the extremities extending into the small bones of the feet. They showed that there was an inverse correlation between bone marrow uptake extent and histolopathological grade of fibrosis, and an inverse correlation between extent of metabolically active osseous disease and time since diagnosis of MF, suggesting that grade 4 corresponded to early and highly active state of the disease, while grade 1 was observed when a burn-out state of the disease was reached, with established fibrosis resulting from the longstanding BM inflammation*.*

They also retrospectively reviewed pre and post-SCT FDG PET in 12 patients [2]. In most patients with complete response, FDG uptake in bone marrow and spleen decreased, whereas it did not significantly change in patients with partial response or not responding disease.

Despite these promising results, they emphasized that the interpretation of FDG uptake in MF could be difficult, because FDG uptake caused by inflammation and by myelopoiesis could not be discriminated. Besides, FDG uptake in BM can be influenced by other factors, such as infection, or injection of granulocyte-colony stimulating factor.

**18F-FLT PET in myelofibrosis**

18F-FLT is a tracer of cell proliferation, mostly used in the oncological setting for staging and therapeutic evaluation. It has also been proved useful to identify the toxicity of radiotherapy and chemotherapy on BM, illustrating the ability of the tracer to evaluate BM function [3].

It could provide some complementary information in the context of MF. Indeed, 18F-FLT PET is expected to become more and more pathological while the condition worsens [4].

In a pilot study on 15 patients, three patterns of FLT distribution in MF were described, from a slight extension of hematopoiesis with normal uptake in axial skeleton, to a major decrease of uptake in axial skeleton, with or without extension in long bones, and increased uptake in the spleen [5]. Though the patterns did not correlate with histological grade of fibrosis, SUVmax in axial BM did, suggesting that severity of disease could be assessed non-invasively with FLT PET. Besides, splenic myeloid metaplasia could be easily assessed, when compared with standard bone marrow scintigraphy, with a high FLT uptake.

**References**

1. Derlin T, Alchalby H, Bannas P, et al (2015) Assessment of bone marrow inflammation in patients with myelofibrosis: an 18F-fluorodeoxyglucose PET/CT study. Eur J Nucl Med Mol Imaging 42:696–705 . doi: 10.1007/s00259-014-2983-4

2. Derlin T, Alchalby H, Bannas P, et al (2016) Serial 18F-FDG PET for Monitoring Treatment Response After Allogeneic Stem Cell Transplantation for Myelofibrosis. J Nucl Med Off Publ Soc Nucl Med 57:1556–1559 . doi: 10.2967/jnumed.115.166348

3. Agool A, Slart RH, Thorp KK, et al (2011) Effect of radiotherapy and chemotherapy on bone marrow activity: a 18F-FLT-PET study. Nucl Med Commun 32:17–22

4. Agool A, Schot BW, Jager PL, Vellenga E (2006) 18F-FLT PET in hematologic disorders: a novel technique to analyze the bone marrow compartment. J Nucl Med 47:1592–8

5. Vercellino L, Ouvrier MJ, Barré E, et al (2017) Assessing Bone Marrow Activity in Patients with Myelofibrosis: Results of a Pilot Study of (18)F-FLT PET. J Nucl Med Off Publ Soc Nucl Med 58:1603–1608 . doi: 10.2967/jnumed.116.188508

### 11:00 – 12:30 Abdominal Oncology

#### A6 Artificial intelligence and machine learning in pelvic tumours

##### Gigin Lin

###### Chang Gung Memorial Hospital, Linkou, Taiwan

This talk reviews the critical concepts of artificial intelligence (AI) and machine learning, describes emerging applications in pelvic tumours, and outlines the limitations and future directions in this field. The aim is to help the audience to integrate machine learning into clinical workflow.

AI and machine learning refer to computer algorithms that improve with the exposure of more data, and deep learning is the subtype that attracts particular interest in radiology. Deep learning has the benefit of not requiring feature identification and calculation as a first step; instead, features are identified as part of the learning process. For computer vision tasks such as radiology, convolutional neural networks (CNN) have proven to be effective. The weightings of connections between nodes or neurons are iteratively adjusted based on the inputs and target outputs by back-propagating a corrective error signal through the network.

Recently, several clinical applications of machine learning have been proposed and studied in pelvic tumours. Most prominent applications are found in prostate cancer, including tumour detection, segmentation and computer-aided diagnosis. Machine learning also helps in tumour segmentation in rectal and cervical cancers. Radiologists who become familiar with the principles and potential applications of machine learning would have the opportunity to leverage AI to become a centre of diagnostic information to improve patient outcomes.

#### A7 GIST – Imaging of Gastrointestinal Stromal Tumors

##### Dietmar Tamandl

###### Department of Biomedical Imaging and Image-Guided Therapy, Medical University of Vienna, Vienna, Austria

Imaging requirements for Gastrointestinal Stromal Tumors (GIST) are quite challenging due to the spare occurrence of this tumor entity and its biology, which differs from most epithelial cancers. Also, patterns of appearance and response to treatment are somewhat unique and therefore require distinct knowledge for the treating multidisciplinary team including the radiologist.

GIST arise from the interstitial cells of Cajal and are characterized by expression of c-KIT and PDGFR-A; mutations in these genes drive tumor biology and define the medical treatment that can be effective only if certain prerequisites exist. GIST can occur anywhere in the GI tract, yet the most common location is the stomach with over 60% of primary GIST diagnosed there. The role of radiology is detection/specification of tumors, securing diagnosis by performing biopsies if adequate, response assessment and follow up.

For diagnosis, multidetector CT (MDCT) is the modality that should be used in the primary workup, with specific technical requirements that need to be followed when performing the scans. MRI is used mainly to assess liver involvement (especially in situations where liver-directed therapies are considered), in rectal GIST and in young patients requiring multiple follow-up examinations. Biopsies should always be performed after consultation with the primary treating physician since the method of choice remains to be endoscopic ultra-sonography-guided biopsy. Percutaneous biopsy approaches should only be performed in situations where no endoscopic biopsy is possible since seeding has been reported quite frequently and thus should be omitted.

Response assessment to treatment with tyrosine kinase inhibitors is one of the major tasks for the radiologists – no other clinical tool enables to monitor treatment effect in this disease. Specific response criteria have been developed, which, quite in opposite to RECIST criteria, take also qualitative tumor appearance into account. Knowledge of those “Choi” criteria is crucial when determining treatment response and examples of pit-falls will be presented. PET/CT and MRI is used in this setting as adjunct modalities, since the Choi criteria are only valid for CT interpretation. An outlook will be given about novel strategies to better assess response in the future.

Follow-up is also a crucial topic in GIST since those patients might require many (20 or more) follow-up scans after initial tumor clearance. Individual recurrence risk, which can be predicted and calculated, should be taken into account when recommending the next follow up scan. Some patients will require tight follow-up and some will require no follow-up at all, based on their individual recurrence risk.

In conclusion, radiology plays a central role in the management of GIST and helps the clinician to reach the correct management decisions in the primary diagnosis, during response assessment and in the follow-up of this challenging tumor entity.

### 13:30 – 14:00 Keynote Lecture 1

#### K1 Big data initiatives changing the future of cancer imaging and therapy

##### F. Prior (FWPrior@uams.edu)

###### Department of Biomedical Informatics, University of Arkansas for Medical Sciences, Little Rock, Arkansas, USA

Since the turn of the millennium biomedical research has undergone a fundamental transition to large-scale experiments and trials that produce massive volumes of highly complex data. “Big data” has been characterized in a four-dimensional space defined by: volume, variety, velocity and veracity. Dealing with this data deluge requires new tools and new approaches to data: capture, curation, storage, search, sharing, transfer, analysis, and visualization. Big data analysis methods enable researchers to identify new directions for research and provide critical support for the development of precision methods for healthcare.

Cancer is a complex, multifactorial disease state. Medical Imaging, particularly Radiology and Pathology imaging, provide valuable diagnostic and prognostic indicators. Imaging based cancer research is undergoing a transition from analyses based on human observers to the use of advanced computing platforms and software to automatically extract quantitative features relevant to prognosis or treatment response. Identifying quantitative imaging phenotypes across scales through the use of robust image analysis and deep learning methods is an evolving approach to improving our understanding of cancer biology. Such methodologies require large collections of well-curated data for development, validation and to ensure research reproducibility. Thanks to advances in imaging technologies and computational platforms, we are able to capture high resolution images in a variety of modalities and compute large volumes of imaging features. This information explosion mandates the need to build a systematic, integrated, reproducible way to evaluate cancer diagnosis, prognosis and treatment.

Prediction of response to treatment and development of optimized therapies require integration of Radiology and Pathology imaging information, molecular and genomics data, and clinical information. High quality imaging feature sets from Radiology, (radiomics) and Pathology images, (pathomics) play a critical role in the development of robust and predictive models for characterization of cancer onset and progression. Clinical and research studies now have greater opportunities to incorporate high throughput generation, mining, and integration of imaging features in their research protocols to aid optimization of treatments and better prediction of response to treatment.

As basic and translational investigators dig deeper into the complexity of underlying disease processes, larger sample populations are required for research studies and increasing quantities of data are being generated. In addition, well-curated research and trial data are being pooled in public data repositories to support large-scale analyses. Our ability to extract new knowledge from huge volumes of research data is the key to advances in cancer diagnosis and treatment.

1. Chennubhotla C, Clarke LP, Fedorov A, Foran D, Harris G, Helton E, Nordstrom R, Prior F, Rubin D, Saltz JH, Shalley E, Sharma A. An Assessment of Imaging Informatics for Precision Medicine in Cancer. Yearbook of Medical Informatics. 2017:26(01):110-119.
2. Rosenstein B, Capala J, Efstathiou J, Hammerbacher J, Kerns S, Kong F-M, Ostrer H, Prior F, Vikram B, Wong J, Xia Y, How Will Big Data Improve Clinical and Basic Research in Radiation Therapy? International Journal of Radiation Oncology*Biology*Physics. 2016;95(3):895-904; 10.1016/j.ijrobp.2015.11.009
3. Kalpathy-Cramer J, Freymann J, Kirby J, Kinahan P, Prior F, Quantitative Imaging Network Data Sharing and Competitive Algorithm Validation Leveraging The Cancer Imaging Archive. Trans Onc, 2014; 7(1), 147-152.
4. Thrall JH. Trends and Developments Shaping the Future of Diagnostic Medical Imaging: 2015 Annual Oration in Diagnostic Radiology. Radiology. 2016;279(3):660-6. doi: doi:10.1148/radiol.2016160293.
5. Chatellier G, Varlet V, Blachier-Poisson C. “Big data” and “open data”: What kind of access should researchers enjoy? Thérapie. 2016;71(1):107-14
6. Hitzler, P. and Janowicz, K., Linked Data, Big Data, and the 4th Paradigm. *Semantic Web*. 2013;*4*(3):233-235.

### Afternoon Session: 14:00 – 15:30 Imaging Musculoskeletal Cancer

#### A8 Radiological response assessment of soft tissue sarcomas

##### Christina Messiou^1,2^ (Christina.Messiou@rmh.nhs.uk)

###### ^1^Department of Radiology, The Royal Marsden NHS Foundation Trust, Fulham Rd, London, UK; ^2^Division of Radiotherapy and Imaging, The Institute of Cancer Research, 15 Cotswold Road, Sutton, UK

**Primary Soft Tissue Sarcoma**

Limb-sparing surgery is potentially curative for primary non-metastatic extremity soft tissue sarcomas. However, the addition of radiotherapy results in higher local control rates [1].

MRI is standard imaging before and after radiotherapy and is critical for surveillance of often complex post treatment appearances. However, histopathologic changes that occur after radiotherapy confound dimension-based assessments of response. Except for myxoid liposarcoma, significant dimensional radiologic responses are rare. Dimension-based assessments have been shown to have no correlation with outcome or histopathologic response, and alternative assessments are needed [2].

Current consensus from the EORTC-STBSG (European Organization for Research and Treatment of Cancer Soft Tissue and Bone Sarcoma Group) suggests that the addition of functional diffusion-weighted MRI to standard anatomic and post contrast MRI greatly facilitates interpretation because the combination of reduced enhancement and increasing apparent diffusion coefficient (ADC) may increase confidence for response. After radiotherapy, new enhancement alone should be interpreted with caution because this may be a result of vascular disruption rather than histologic response [3]. Machine learning is likely to play a crucial role in allowing us to fully exploit the quantitative capabilities for use in routine, research and adaptive treatment regimens.

Increasing conversations in the sarcoma community regarding the role for neoadjuvant chemotherapy in primary soft tissue sarcoma has highlighted the need for an early marker of response. Preliminary data on FDG PET/CT has been promising, indicating that FDG PET/CT predicts histopathologic responses after one cycle of neoadjuvant chemotherapy as well as predicting survival [4].

**Metastatic Soft Tissue sarcoma**

CT is the mainstay of surveillance and response assessment for metastatic disease. However, patients with the best radiological responses by RECIST 1.1 often have the worst survival outcomes. This is because patients with high grade sarcomas respond very well to chemotherapy but responses are short lived followed by rapid deterioration and short overall survival [5]. This highlights the need to appreciate the significance of imaging responses with regards to outcome. A novel alternative approach to interpretation of RECIST 1.1 response assessments is to shift focus from response to absence of progression. A large EORTC database study has shown that absence of progression in patients with metastatic soft tissue sarcoma does indicate survival benefit [6]. One of the most promising features of Choi criteria, which incorporate size and density for response assessment in Gastrointestinal Stromal Tumours (GIST), is the correlation between response by Choi criteria with improved survival [7]. There is also evidence to suggest that Choi criteria may also be useful in non-GIST sarcoma response assessment [8].

1. Haas RL, Gronchi A, van de Sande MAJ et al. Perioperative management of extremity soft tissue sarcomas. JCO 2018; 36:118-124.
2. Miki Y, Ngan S, Clark JC et al. The significance of size change of soft tissue sarcoma during preoperative radiotherapy. Eu J Surg Onc 2010; 36:678-83.
3. Messiou C, Bonvalot S, Gronchi A et al. Evaluation of response afterpre-operative radiotherapy in soft tissue sarcomas: the EORTC-STBSG and Imaging Group recommendations for radiological examination and reporting with an emphasis on magnetic resonance imaging. Eu J Cancer 2016; 56: 37-44.
4. Herrmann K, Matthias R, Czernin et al. 18F FDG-PET-CT imaging as an early survival predictor in patients with primary high grade soft tissue sarcomas undergoing neoadjuvant therapy. Clin Cancer Research 2012; 18:2024-2031.
5. Sleijfer S, Ouali M, Glabbeke M et al. prognostic and predictive factors for outcome to first-line ifosfamide-containing chemotherapy for adult patients with advanced soft tissue sarcomas. Eu J Cancer 2010; 46:72-83.
6. Cesne A, Van Glabbeke M, Verweij J et al. Absence of progression by response evaluation criteria I solid tumours predicts survival in advanced GI stromal tumours treated with imatinib mesylate: The Intergroup EORTC-ISG-AGITG phase III trial. JCO 2009; 27:3969-74.
7. Benjamin R, Choi H, Homer A et al. We should desist using RECIST at least in GIST.JCO 2007; 25: 1760-4.
8. Stacchioti S, Verderio P, Messina A et al. Tumour response assessment by modified choi criteria in localized high-risk soft tissue sarcoma treated with chemotherapy. Cancer 2012;118: 5857-66.

#### A9 Pediatric Bone and Soft-Tissue Sarcomas

##### MB McCarville

###### St. Jude Children’s Research Hospital, Memphis, TN, USA

Imaging plays a central role in the diagnosis, staging and management of bone and soft-tissue sarcomas in children. When taken together with clinical findings, imaging can narrow the differential diagnosis and, thus, guide the diagnostic work-up. The most common bone and soft-tissue sarcomas affecting children include osteosarcoma, Ewing sarcoma family of tumors and rhabdomyosarcoma [1,2]. These tumors present unique imaging challenges that are not encountered with other solid malignancies, especially regarding assessment of response to therapy. Most notably, bone tumors do not significantly decrease in size, even when they respond well to therapy. Therefore, the conventional method of assessing solid tumor response (i.e. the RECIST criteria) may not reflect true therapeutic response in bone tumors [2]. Furthermore, response to neoadjuvant therapy, prior to local control of rhabdomyosarcoma has not accurately predicted patient outcome [3]. In this lecture I will review the salient imaging and clinical features that are useful in guiding patient management and will briefly discuss challenges and future directions in assessing pediatric sarcoma response to therapy.

1. Hawkins DS, Bolling T, Dubois S, Hogendoorn PCW, Jurgens H, Paulussen M, Randall L, Lessnick SL. Ewing Sarcoma. In Principles and Practice of Pediatric Oncology. 6^th^ Ed. Pizzo and Poplack Editors. 2011 Lippincott, Williams and Wilkins; 987-1015
2. Wexler LH, Meyer W, Helman LJ. Rhabdomyosarcoma. In Principles and Practice of Pediatric Oncology. 6^th^ Ed. Pizzo and Poplack Editors. 2011 Lippincott, Williams and Wilkins; 923-954.
3. Degnan AJ, Chung CY, Shah AJ. Quantitative diffusion-weighted magnetic resonance imaging assessment of chemotherapy treatment response of pediatric osteosarcoma and Ewing sarcoma malignant bone tumors. Cinical Imaging, 2018, Volume 47, Pages 9-13
4. Crist WM, Anderson JR, Meza JL, Fryer C, Raney RB, Ruymann FB, Breneman J, Qualman SJ, Wiener E, Wharam M, Lobe T, Webber B, Maurer HM, Donaldson SS. Intergroup Rhabdomyosarcoma Study-IV: Results for Patients With Nonmetastatic Disease. Journal of Clinical Oncology, Vol 19, No 12 (June 15), 2001: pp 3091-3102

#### A10 Imaging of bone metastases

##### Giuseppe Petralia^1-2^ (giuseppe.petralia@ieo.it)

###### ^1^European Institute of Oncology - IEO, Milan, Italy; ^2^University of Milan, Milan, Italy

In the era of precision oncology, we aim to give the right treatment, to the right patient, for the right time and the right duration. Next generation diagnostic, including imaging and genomics, as well as blood, serum and tissue samples, will be needed to drive next generation clinical trials based on molecular pathology, and will enable to have a measure of tumour activity.

One of the main challenges for the next generation diagnostics will be the management of bone metastases, as bone is the first site of metastases for the most common cancers in men and women (prostate and breast). Current imaging tools for bone assessment are limited for detecting presence, volume, viability and response of metastatic bone disease, and may not be adequate for promoting precision oncology. BS has limited sensitivity compared to PET/CT and WB-MRI^1^ (78% vs 91% vs 97%, respectively) and does not directly evaluate malignant bone disease. Due to the risk of flare responses, any progressive disease (PD) has to be confirmed on a subsequent BS examination. CT shows osteoblastic and osteolytic activity, but it still shows limitations: RECIST criteria are not applicable for bone metastases (they are considered not measurable^2^), and osteosclerotic progression and response are difficult to be distinguished. Such limitations for current imaging tools may result in a delayed detection of PD. WB-MRI has demonstrated the potential to detect PD earlier than BS or CT^3^, thus promoting timely interventions and therapeutic changes.

Another big challenge for the next generation diagnostics will be capturing tumour heterogeneity. Cancer evolves in time and space, in a kind of Darwinian process^4^. Various factors drive this selection, including anti-cancer therapies, and this evolutionary process eventually leads to the formation of metastases. MET-RADS guidelines^5^ for reporting of WB-MRI enable recording of the presence, location and extent of discordant responses in bone metastases, that could be linked to a different gene expression or to the emergence of clonal resistance, and therefore to tumour heterogeneity.

**References**

1. Shen G, et al., Skeletal Radiol. 2014 Nov;43(11):1503-13.
2. Eisenhauer EA et al. - Eur J Cancer. 2009.
3. Kosmin M, et al. Eur J Cancer. 2017 May;77:109-116.
4. Gundem G et al. Nature. 2015 Apr 16;520(7547):353-357.
5. Padhani AR et al. Eur Urol. 2017 Jan;71(1):81-92.

### 16:00 – 17:30 Imaging Immunotherapy

#### A11 FDG PET/CT for Monitoring of Immunotherapy

##### Rodney J. Hicks

###### The Sir Peter MacCallum Department of Oncology the University of Melbourne, Parkville, Australia

Immunotherapy has become the latest addition to oncological therapeutics. A number of different agents are entering clinical trials but anti-CTLA-4 and anti-PD1/PDL1 agents have been the first to gain regulatory approval and reimbursement in many countries. In all but a few malignancies that have either a high mutational burden or incorporation of viral DNA, overall response rates are low. Nevertheless, immunotherapy has captured the public imagination because of apparent “miracle” cures in patients who have failed all other available therapies. Despite its promise, immunotherapy has significant direct financial costs and, secondarily, those related to management of a wide array of immune-related toxicities. Accordingly, early identification of non-responders could ameliorate the negative impact of ineffective or toxic therapy. In particular, early recognition of immune-related toxicity could prevent life-threatening complications. Conventional imaging with CT and MRI has been recognized to be suboptimal for early immunotherapy response assessment and has led to recent adaptions of response criteria for such treatment. The use of FDG PET/CT has also come into focus for this purpose but is similarly challenged by the unique mechanisms of response to immunotherapy. A significant issue for both anatomical and molecular imaging is the phenomenon of pseudoprogression. For cancer imaging specialists, it is important to understand that the two broad classes of immunotherapy, anti-CTLA4 and Anti-PD-1/PDL-1 agents, have different mechanisms of action. The former acts primarily on the afferent limb of immune response to expand and recruit immune cells into tumour deposits, whereas the latter enhance immune-cell killing by diminishing immune checkpoints. Accordingly, the anti-CTLA-4, in particular, can lead to a transient increase in metabolic activity and lesion enlargement due to increased infiltrating lymphocytes that precede cell killing as reflected by progression followed by regression, which is the process known as pseudoprogression. The anti-PD1/PDL1 agent generally leads to tumour cells being cleared and a consequent reduction in FDG-uptake paralleling morphological regression. In this regard, these agents lead to responses that are more similar to response to more conventional cytotoxic therapies. When these agents are combined, the degree and temporal profile of these conflicting tendencies to increase or decrease global lesional FDG accumulation can make interpretation more difficult. There can, however, be additional clues that can help to differentiate between true and pseudoprogression. These include development of new lymph node abnormalities in the drainage basin of otherwise stable or responding lesions and activation of lymphoid organs, especially the spleen. Similarly, immune-related complications of therapy are manifold and more common with anti-CTLA4 agents than with anti-PD-1/PDL-1 agents. It is important to be aware of these complications and to conscientiously evaluate scans for them. Most important of these, since they can have fatal consequences are colitis and pneumonitis but other important complications include endocrinopathies, hepatitis and rheumatological disorders. Novel agents are emerging that may further enhance the role of molecular imaging not only in monitoring response but also potentially in selecting patients for treatment with these agents and to identify sites that might benefit from combination therapies. There is, in particular, growing interest in the possibility of enhancing abscopal effects through increased neo-antigenic challenge following radiation of lesions, which could be external or internal.

**References**

1. Kee, D. and G. McArthur, Immunotherapy of melanoma. *European Journal of Surgical Oncology*, 2017;43:594-603.
2. Wong A, McArthur GA, Hofman MS, Hicks RJ. Invited Review: The Advantages and Challenges of Using FDG PET/CT for Response Assessment in Melanoma in the Era of Targeted Agents and Immunotherapy. *The European Journal of Nuclear Medicine and Molecular Imaging,* 2017;44(Suppl 1):67-77.
3. Sharabi, A.B., et al., Radiation and checkpoint blockade immunotherapy: radiosensitisation and potential mechanisms of synergy. *Lancet Oncology*, 2015. 16:e498-509.

#### A12 Complications of immunotherapy

##### Richard M. Gore, Silvers RI, Daniel R. Wenzke, Kiran H. Thakrar, Gregory P. Jackson

###### Department of Radiology, North Shore University Health System-University of Chicago, Evanston, IL, USA

####### **Correspondence:** Richard M. Gore (rgore@uchicago.edu)

**Introduction:** Over the last 10 years, the management of cancer patients has been revolutionized by the advances in molecular targeted therapy and immunotherapy with significant benefits for patient outcomes and comfort. These therapies however are associated with new toxicities and complications that: can be mild, moderate or life-threatening; may require alteration or cessation of therapy; or simulate disease progression. In this presentation the various classes of immunotherapy associated with pulmonary complications are reviewed and the drug-associated injuries and their differential diagnosis are presented.

**Pneumonitis:** Drug induced pneumonitis develops in up to 10% of patients on immunotherapy and remains a diagnosis of exclusion that must be differentiated from infection and malignant lung infiltration. Five different patterns have been described on CT: ground glass opacities with preserved bronchovascular markings; increased interstitial markings, interlobular septal thickening, peribronchovascular infiltration, subpleural reticulation, and honeycomb pattern in severe cases ; cryptogenic organizing pneumonia-like, with discrete patchy or confluent consolidation with or without air bronchograms, predominantly peripheral or subpleural in location; non-specific, with a mixture of nodular and other subtypes, not clearly fitting into other subtype classifications.

**Bronchiolitis Obliterans:** There is myxoid fibrous tissue filling the distal bronchioles and extending into alveolar ducts and associated with inflammatory cells. On CT imaging findings include: bilateral regions of patchy consolidation or small irregular nodular opacities, bronchial wall thickening and dilation, and small pleural effusions.

**Radiation Recall Pneumonitis:** This is an inflammatory reaction in previously irradiated areas of lung producing well defined areas of alveolar consolidation, ground glass opacities or infiltrates corresponding to the radiation portals. This pneumonitis usually presents 3-4 months following radiotherapy and the patient presents with cough and dyspnea.

**Pulmonary Veno-Occlusive Disease**: Progressive occlusion of postcapillary pulmonary venules leads to increased pulmonary resistance, pulmonary hypertension, and right ventricular failure. CT findings include diffuse ground-glass opacification, septal thickening, peribronchial thickening, soft tissue oedema around the hila and mediastinum, small pleural effusions, and dilatation of the central pulmonary arteries.

**Sarcoid-Like Granulomatous Reactions:** Intrathoracic lymphadenopathy simulating sarcoidosis develops in up to 10% of patients following ipilimuab and nivolumab therapy. The adenopathy may manifest and newly enlarged lymph nodes or enlargement of pre-existing lymph nodes that occur in isolation or associated with bilateral upper lobe and middle lobe predominant ground glass opacities, parenchymal consolidations and/or irregular nodules. Most patients are asymptomatic and biopsy show non-caseating granulomas with elevated CD4:CD8 levels. Extrathoracic diffuse adenopathy and cutaneous non-caseating granulomas have also been described.

**Pseudoprogression:** Immunotherapy often may initially provoke infiltration of cytotoxic T lymphocytes and other immune cells into the tumor bed. This may cause an increase in tumor size or the development of new lesions as an early response. Pseudoprogression is defined as ≥ 25% increase in tumor burden that is not seen on repeat imaging performed 4 weeks or more after the initial study. Mixed immune-related responses or pseudoprogression are quite problematic in assessing treatment response using RECIST criteria.

This abstract has been previously published [4].

**References**

1. Kroschinsky F, Stolzel F, von Bonin S, et al: **New drugs, new toxicities: severe side effects of modern targeted and immunotherapy of cancer and their management.** *Critical Care* 2017; **21**: 89—100.

2. Shannon VR: **Pneumotoxicity associated with immune checkpoint inhibitors**. *Curr Opin Oncol* 2017; **23**: 1-18.

3. Tabchi S, Messier C, Blais N: **Immune-mediated respiratory adverse events of checkpoint inhibitors**. *Curr Opin Oncol* 2017; **28:** 269-277.

4. Gore RM, Silvers RI, Wenzke DR, Thakrar KH, Jackson GP. Proceedings of the International Cancer Imaging Society (ICIS) 17th Annual Teaching Course. In *Cancer Imaging* 2017 Sept (Vol. 17, No. 1, p. 24). BioMed Central.

#### A13 Cancer imaging in drug development: Focus on immunotherapy

##### Annick D. Van den Abbeele^1,2,3,4^ (abbeele@dfci.harvard.edu)

###### ^1^Department of Imaging and Center for Biomedical Imaging in Oncology, Dana-Farber Cancer Institute, Harvard Medical School, Boston, MA, USA; ^2^Division of Cancer Imaging, Department of Radiology, Brigham and Women’s Hospital, Harvard Medical School, Boston, MA, USA; ^3^Tumor Imaging Metrics Core, Dana-Farber/Harvard Cancer Center, Harvard Medical School, Boston, MA, USA; ^4^Cancer Imaging, International Cancer Imaging Society, London, United Kingdom

Bringing novel cancer drugs from discovery to regulatory approval is a lengthy, expensive and high-risk undertaking that takes about 10 to 15 years to complete with risk of failure at each step. Oncology leads the number of products in clinical development with more than 4,000 projects as of August 2016 [1]. However, only approximately 12% of the drugs entering the clinical trial phase make it to regulatory submission [2], and the average cost to yield a single FDA-approved drug has been reported to range from 648 million USD [3] to as high as 2.6 billion USD [4]. New strategies that can help identify promising new drug candidates early, while eliminating those that are unlikely to be successful could significantly improve the overall drug development process [5].

Cancer imaging is well positioned to make a significant impact on the different phases of drug development, and shorten the timeline between discovery, pivotal preclinical/clinical trials and regulatory approval [6,7]. Current multimodality anatomic, functional, and molecular imaging techniques provide longitudinal whole-body evaluations of the entire tumor burden in one setting and noninvasive in vivo characterization and quantification of biologic, metabolic and molecular processes in the tumor and its microenvironment equivalent to a “virtual biopsy”. Cancer imaging technologies can, therefore, serve as a biomarker with potential prognostic value, help enrich trials with proper patient selection and stratification, confirm target hit (or lack thereof) within hours or days after initiation of treatment with potential predictive value, and detect primary and secondary resistance [2-8].

One of the most exciting recent breakthrough in cancer therapy is the introduction of cancer immunotherapy with immune checkpoint modulators (anti-CTLA4, anti-PD1 and anti-PD-L1), bispecific antibodies, T cell therapy and personalized cancer vaccines [9-12]. This novel therapeutic strategy can be a “game-changer” for patients with a variety of solid and liquid tumors with some patients achieving long term survival. This paradigm shift has also instigated a wide range of challenges for cancer imaging to minimize translational failures, assess response (or lack thereof), and evaluate toxicity [13,14]. Adding specificity to the tumor microenvironment by developing robust and predictive biomarkers that can assess the presence and phenotypes of immune cells and their activation status prior to and after treatment would greatly help stratify patients and differentiate inflammatory response from tumor progression. These immune imaging biomarkers could be validated as companion diagnostics, and serve as a pharmacodynamic marker, or “virtual biopsy” in integrated prospective preclinical/clinical studies, such as small phase I co-clinical trials that can inform the conduct of each trial bi-directionally, and where pre- and post-biopsies are taken and appropriate clinical trial endpoints are chosen. This approach would help assess who to treat, and identify promising drug candidates and combinations with the greatest likelihood of success, efficacy and safety.

The systemic inclusion of cancer imaging techniques and probes in prospective pre-clinical/clinical trials or co-clinical trials would greatly contribute to, and increase the successful translation of the development of novel immunotherapeutic drugs for cancer patients in a safe, timely and cost-effective manner.

**References**

1. **The biopharmaceutical pipeline: innovative therapies in clinical development.** Pharmaceutical Research and Manufacturers of America website. http://phrmadocs.phrma.org/files/dmfile/Biopharmaceutical-Pipeline-Full-Report.pdf. Updated July 2017. Accessed May 1, 2018.
2. Waaijer SJH, Kok IC, Eisses B, Schröder CP, Jalving M, Brouwers AH, Lub-de Hooge MN, de Vries EGE. **Molecular Imaging in Cancer Drug Development.** *J Nucl Med.* 2018 Jan 25. pii: jnumed.116.188045. doi: 10.2967/jnumed.116.188045. [Epub ahead of print] PMID:29371402
3. Prasad V, Mailankody S. **Research and development spending to bring a single cancer drug to market and revenues after approval**. *JAMA Intern Med*. 2017;**177**: 1569–1575
4. DiMasi, JA, Grabowski, HG, and Hansen, RW. **Innovation in the Pharmaceutical Industry: New Estimates of R&D Costs**. *Journal of Health Economics* 2016;47:20-33
5. **Willmann** JK, van Bruggen N, Dinkelborg LM, Gambhir SS. **Molecular imaging in drug development.** *Nat Rev Drug Discov*. **2008** Jul;**7**(7):591-607. doi: 10.1038/nrd2290. Review. PMID:18591980
6. Van den Abbeele AD, Krajewski KM, Tirumani SH, Fennessy, DiPiro PJ, Nguyen QD, Harris GJ, Jacene HA, Lefever G, Ramaiya NH. **Cancer Imaging at the Crossroads of Precision Medicine: Perspective from an Academic Imaging Department in a Comprehensive Cancer Center**. *J Am Coll Radiol.* 2016 Apr;**13**(4):365-71. PMID: 26774886
7. Hricak H. **2016 New Horizons Lecture: Beyond Imaging-Radiology of Tomorrow** *Radiology* Mar;**286**(3):764-775. doi: 10.1148/radiol.2017171503. Epub 2018 Jan 18. PMID: 29346031
8. Van den Abbeele AD. **The Lessons of GIST--PET and PET/CT: A new paradigm for imaging.** *Oncologist* 2008, **13** Suppl 2:8-13. PMID 18434632
9. Hodi, F. S. *et al*. **Improved survival with ipilimumab in patients with metastatic melanoma.** *N. Engl. J. Med.* 2010, **363**, 711–723.
10. Topalian, S. L. *et al*. **Safety, activity, and immune correlates of anti-PD-1 antibody in cancer.** *N. Engl. J. Med.* 2012, **366**, 2443–2454.
11. Androulla MN, Lefkothea PC. **CAR T-cell Therapy: A New Era in Cancer Immunotherapy.** *Curr Pharm Biotechnol*. 2018 Apr 17. doi: 10.2174/1389201019666180418095526. [Epub ahead of print] PMID: 29667553
12. Sahin U, Türeci Ö. **Personalized vaccines for cancer immunotherapy.** *Science***.** 2018 Mar 23;**359**(6382):1355-1360. doi: 10.1126/science.aar7112. Review. PMID: 29567706
13. Kwak JJ, Tirumani, SH, Van den Abbeele AD, Koo PJ, Jacene HA. **Cancer Immunotherapy: Imaging Assessment of Novel Treatment Response Patterns and Immune-related Adverse Events**. *Radiographics*. 2015 Mar-Apr;**35**(2):424-37. doi: 10.1148/rg.352140121. PMID: 25763727
14. Carter, B. W., Bhosale, P. R. and Yang, W. T. **Immunotherapy and the role of imaging**. *Cancer* 2018. doi:10.1002/cncr.31349

### 16:00 – 17:30 Interventional Radiology

#### A14 Image guided biopsy; how I do it

##### Jurgen J. Fütterer (Jurgen.Futterer@radboudumc.nl)

###### Department of Radiology and Nuclear Medicine, Radboudumc, Nijmegen, The Netherlands

Diagnosis of prostate cancer by systematic TRUS-guided biopsies has led to a substantial raise of the detection of clinically insignificant tumors [1]. Which has resulted in overtreatment and overdiagnosis of prostate cancer. Furthermore, TRUS-guided biopsies misses a substantial proportion of significant tumors because of sampling error, especially in the anterior part and apical region of the prostate [2]. This is related to the fact that TRUS, when performed as a first line imaging modality, lacks both sensitivity and specificity in the detection of cancer foci. Conversely, the high accuracy of multiparametric MRI for localizing significant prostate cancers has been established and MR findings can thus be used to target any suspicious prostatic area to improve the detection of aggressive tumors and to avoid the detection of non-significant tumors.

References:

1. Schroder FH, Hugosson J, Roobol MJ, Tammela TL, Ciatto S, Nelen V, et al. Screening and prostate-cancer mortality in a randomized European study. The New England journal of medicine. 2009;360(13):1320-8.
2. Schouten MG, van der Leest M, Pokorny M, et al. Why and Where do We Miss Significant Prostate Cancer with Multi-parametric Magnetic Resonance Imaging followed by Magnetic Resonance-guided and Transrectal Ultrasound-guided Biopsy in Biopsy-naïve Men? Eur Urol. 2017 Jun;71(6):896-903.

#### A15 Percutaneous non-vascular interventions in GU oncology

##### Bernhard Gebauer (bernhard.gebauer@charite.de)

###### Charité Universitätsmedizin Berlin, Radiological Clinic, Charité Campus Virchow-Klinikum, Augustenburger Platz 1, Berlin, Germany

In genitourinary (GU) cancers, like prostate, bladder and kidney many non-vascular interventions are established. The most frequent used non-vascular interventions are diagnostic punctures (in kidney and prostate cancer) and thermal (radiofrequency (RFA), microwave (MWA) or cryotherapy (CRYO)) or non-thermal (irreversible electroporation (IRE)) ablations of kidney and prostate cancer.

In kidney cancer the main indication for local ablative therapy are T1a (≤ 4 cm in diameter) tumors, especially in patients with reduced renal function or single kidney. The main competitor of local ablation in the kidney is nephron-sparing surgery or partial nephrectomy (PN). In a retrospective analysis of 1424 T1aN0M0 renal cell cancers treated by PN, RFA or CRYO in the Mayo Clinic the local recurrence-free survival was similar between all three techniques, whereas the metastasis-free survival was significantly better with PN and CRYO compared to RFA. But RFA and CRYO patients were in mean more than 10 years older than PN patients due to the retrospective nature of the study and probably the clinical decision was made to treat the unfitter patients with local ablation and the fitter with PN. Without an prospective randomized trial this clinical selection bias will largely influence the analysis of outcome between surgical and ablative techniques.

Many other studies on renal cell tumor ablation could show very similar results of ablation to surgical resection.

In prostate cancer due to the higher availability of multiparametric prostate MRI the demand of image-guided prostate biopsies is increasing. Two techniques are well established: MRI-guided biopsy and fusion biopsy of transrectal ultrasound and MRI (MRI-TRUS fusion).

Additionally these new technologies are increasing the demand for local treatment of localizes prostate cancer using thermal ablation (high-focused ultrasound (HIFUS)), radioablation (brachytherapy, cyberknife) or biochemical ablation (irreversible electroporation (IRE)).

1. Wah TM. Image-guided ablation of renal cell carcinoma. Clin Radiol. 2017;72(8):636-44.

2. Mir MC, Derweesh I, Porpiglia F, Zargar H, Mottrie A, Autorino R. Partial Nephrectomy Versus Radical Nephrectomy for Clinical T1b and T2 Renal Tumors: A Systematic Review and Meta-analysis of Comparative Studies. Eur Urol. 2017;71(4):606-17.

3. Marcelin C, Ambrosetti D, Bernhard JC, Roy C, Grenier N, Cornelis FH. Percutaneous image-guided biopsies of small renal tumors: Current practice and perspectives. Diagn Interv Imaging. 2017;98(9):589-99.

4. Marconi L, Dabestani S, Lam TB, Hofmann F, Stewart F, Norrie J, Bex A, Bensalah K, Canfield SE, Hora M, Kuczyk MA, Merseburger AS, Mulders PF, Powles T, Staehler M, Ljungberg B, Volpe A. Systematic Review and Meta-analysis of Diagnostic Accuracy of Percutaneous Renal Tumour Biopsy. Eur Urol. 2016;69(4):660-73.

5. Kim HJ, Park BK, Park JJ, Kim CK. CT-Guided Radiofrequency Ablation of T1a Renal Cell Carcinoma in Korea: Mid-Term Outcomes. Korean J Radiol. 2016;17(5):763-70.

6. Thompson RH, Atwell T, Schmit G, Lohse CM, Kurup AN, Weisbrod A, Psutka SP, Stewart SB, Callstrom MR, Cheville JC, Boorjian SA, Leibovich BC. Comparison of partial nephrectomy and percutaneous ablation for cT1 renal masses. Eur Urol. 2015;67(2):252-9.

7. He Q, Wang H, Kenyon J, Liu G, Yang L, Tian J, Yue Z, Wang Z. Accuracy of Percutaneous Core Biopsy in the Diagnosis of Small Renal Masses (</= 4.0 cm): A Meta-analysis. Int Braz J Urol. 2015;41(1):15-25.

8. Cooper CJ, Teleb M, Dwivedi A, Rangel G, Sanchez LA, Laks S, Akle N, Nahleh Z. Comparative Outcome of Computed Tomography-guided Percutaneous Radiofrequency Ablation, Partial Nephrectomy or Radical Nephrectomy in the Treatment of Stage T1 Renal Cell Carcinoma. Rare Tumors. 2015;7(1):5583.

9. Chang X, Liu T, Zhang F, Ji C, Zhao X, Wang W, Guo H. Radiofrequency ablation versus partial nephrectomy for clinical T1a renal-cell carcinoma: long-term clinical and oncologic outcomes based on a propensity score analysis. J Endourol. 2015;29(5):518-25.

10. Katsanos K, Mailli L, Krokidis M, McGrath A, Sabharwal T, Adam A. Systematic review and meta-analysis of thermal ablation versus surgical nephrectomy for small renal tumours. Cardiovasc Intervent Radiol. 2014;37(2):427-37.

11. Volpe A, Finelli A, Gill IS, Jewett MA, Martignoni G, Polascik TJ, Remzi M, Uzzo RG. Rationale for percutaneous biopsy and histologic characterisation of renal tumours. Eur Urol. 2012;62(3):491-504.

#### A16 Biopsy of pancreatic cancer

##### Mirko D’Onofrio

###### Department of Radiology, GB Rossi University Hospital, University of Verona, Verona, Italy

Biopsy and fine-needle aspiration is widely used as an invasive diagnostic technique for solid pancreatic masses. Radiological guidance is required in most cases. Many imaging modalities can be chosen: ultrasound, either trans-abdominal (US) or endoscopic (EUS), computed tomography (CT), or magnetic resonance imaging (MRI). Different factors may influence this choice: availability, safety, and, last but not least, local expertise. Ultrasound provides real-time guidance, and is widely available and biologically safe. Previously published papers have reported high sensitivity and accuracy values for percutaneous US-FNA, with low complication rates. Percutaneous biopsy or FNA can be also performed under CT guidance, but this technique has some drawbacks, such as the lack of real time visualization and the radiation dose. EUS guidance can be also used for FNA; however, it is not available in all institutions, is more expensive than percutaneous FNA, and must be performed at least under deep sedation. No significant differences in terms of diagnostic accuracy have been reported between the endoscopic and percutaneous approaches for the diagnosis of solid pancreatic malignant masses. Percutaneous FNA and EUS-FNA have similar relatively low complication rates, ranging between 0 % and 5 %. Guidelines from EFSUMB society will be analyzed.

This abstract was taken from a previous publication [1].

References

1. D’Onofrio M, De Robertis R, Barbi E, Martone E, Manfrin E, Gobbo S, Puntel G, Bonetti F, Mucelli RP. Ultrasound-guided percutaneous fine-needle aspiration of solid pancreatic neoplasms: 10-year experience with more than 2,000 cases and a review of the literature. *European radiology*. 2016 Jun 1;26(6):1801-7.

### Monday 8^th^ October – Morning Session: 9:00 – 10:30 Clinical needs in Pancreatic Tumours

#### A17 Resectable and non-resectable pancreatic ductal adenocarcinoma

##### IR Francis

###### Department of Radiology, Michigan Medicine, Ann, Arbor, Michigan, USA

**Introduction:**

About 55,440 people will be diagnosed with and approximately 44,330 people will die of pancreatic cancer in the US in 2018. Pancreatic cancer also accounts for about 3% of all cancers in the US and about 7% of all cancer deaths. The 5-year survival rate for people with stage I tumors (confined to the pancreas) the 5-year survival rate is about 12-14%. For stage II pancreatic cancer (spread to less than 3 lymph nodes), the 5-year survival rate is about 5-7%. The 5-year survival rate for stage III pancreatic cancer (spread to more than 3 lymph nodes) is about 3% and for Stage IV pancreatic cancer (distant spread) has a 5-year survival rate of about 1% (American Cancer Society - https://www.cancer.org/cancer/pancreatic-cancer/about/key-statistics.html) [Ref.1].

Decisions regarding resectability status and diagnostic management should involve multidisciplinary consultation at a high volume center with capability of using appropriate high-quality imaging studies for diagnosis and staging [Ref.2 NCCN- version 1.2018]. A structured radiology reporting template that has been approved by the American Pancreatic Association and the Society of Abdominal Radiology has been devised using the morphology and imaging appearances of the tumor and its local and distant spread using imaging findings [Ref.3].

**Resectability Status**

Based on the clinical examination, imaging findings, and at multidisciplinary tumor boards, pancreatic ductal adenocarcinomas, can be categorized into:

1) Resectable 2) borderline resectable and 3) unresectable categories. The unresectable category may be based on a) presence of locally advanced or b) metastatic disease.

**I. RESECTABLE**

A tumor is deemed resectable if:

There is no arterial contact or abutment of the celiac axis (CA), SMA or common (PV)hepatic artery (CHA), nor contact with the SMV or portal vein) or < 180 degrees contact without vein contour deformity/irregularity.

**II. BORDERLINE RESECTABLE**

A tumor is deemed borderline resectable depending on its location and presence of vascular involvement as follows:

1. **Tumor located in pancreatic head/uncinated process:**

- Tumor contact with CHA without extension to CA or hepatic artery bifurcation allowing for resection and arterial reconstruction

- Tumor contact with < 180 degree of SMA

- Tumor contact with variant arterial anatomy (accessory right hepatic or replaced right hepatic artery, replaced CHA, and origin or replaced or accessory artery). Presence and degree of tumor contact should be noted

- Tumor contact with SMV or PV of > 180 degrees, contact of < 180 degrees with vein contour deformity/irregularity or vein thrombosis but suitable vessel proximal and distal to the site of involvement for resection and venous reconstruction

- Tumor contact with IVC

**2. Tumor located in body/tail of pancreas:**

- Tumor contact with CA of < 180 degrees

- Tumor contact with CA of > 180 degrees without aortic involvement and uninvolved GDA (permitting performance of Appleby procedure- controversial- some put this in unresectable category)

**III. UNRESECTABLE**

- Presence of distant metastasis (incl. non regional nodal involvement)

1. **Tumor located in head and uncinate Process**

- Tumor contact with SMA >180 degrees

- Tumor contact with CA > 180 degrees

- Unreconstructible SMV/PV due to tumor involvement or occlusion (can be due to tumor or bland thrombus)

- Tumor contact with most proximal jejunal branch draining into SMV

**2. Body and Tail**

- Tumor contact > 180 degrees with SMA or CA

- Tumor contact with CA and aortic involvement

- Unreconstructible SMV/PV due to tumor involvement or occlusion (can be due to tumor or bland thrombus)

**Limitations of NCCN:**

The NCCN guidelines are a general set of guidelines that are applicable to most cases of pancreatic ductal carcinomas but has its limitations. Not all scenarios are that may be seen in individual cases as there may be many variations of tumor contact with vessels that have not been included. This was addressed in the recent manuscript by Garces-Decovich A et al [Ref. 4). In this report they report that while the NCCN guidelines accurately classified 90% of the cases they reported on, that in 10% of cases, they encountered 6 scenarios of vascular involvement that were not covered in the NCCN guidelines.

In addition, Noda and colleagues have recently reported that the NCCN guidelines are less accurate in predicting resectability when compared to the Japanese Pancreatic Surgery scoring system (Ref.5). In addition, they found that a combination of the NCCN and JPS scores that the highest AUC for distinguishing a R0 resection from a R1/R2 resection.

**Conclusion:**

Overall the designation of resectable, borderline and unresectable categories appears to be suitable for the majority of pancreatic ductal adenocarcinoma, management decisions. But in some scenarios, decisions by management teams at local institutions will have to be made depending on local expertise. The NCCN guidelines are flexible/changeable, and future modifications may be made depending on changing surgical and medical therapies.

**References:**

1. American Cancer Society - https://www.cancer.org/cancer/pancreatic-cancer/about/key-statistics.html.

2. National Comprehensive Cancer Network Guidelines Version 1.2018- Pancreatic Adenocarcinoma.

3. Al-Hawary MM, Francis IR, Chari ST, Fishman EK, Hough DM, Lu DS, Macari M, Megibow AJ, Miller FH, Mortele KJ, Merchant NB, Minter RM, Tamm EP, Sahani DV, Simeone DM. Pancreatic ductal adenocarcinoma radiology reporting template: consensus statement of the Society of Abdominal Radiology and the American Pancreatic Association. Gastroenterology. 2014 Jan;146(1):291-304 and Radiology 2014 Jan;270(1):248-60

4. Garces-Decovich A, Beker D, Jaramillo-Cardoso A et al. Applicability of current NCCN guidelines for pancreatic adenocarcinoma resectability : analysis and pitfalls.

5. Noda Y, Goshima S, Kawada H et al. Modified National Comprehensive Cancer Network criteria for assessing resectability of pancreatic ductal adenocarcinoma. AJR 2018:210:1252-1258

#### A18 Imaging response to treatment

##### Dow-Mu Koh

###### Royal Marsden Hospital, Sutton, Surrey, UK

Pancreatic adenocarcinomas are associated with significant mortality because tumours often present late or with metastatic disease. Patients suitable for surgical resection of the primary disease are associated with better treatment outcomes. In patients with disease of borderline surgical resectability, the use of neoadjuvant chemotherapy can downsize and downstage the tumour to maximise the chance of surgical resection. In this regard, CT and MRI are increasingly used to assess the response of locally advanced pancreatic cancers to neoadjuvant treatment, and the potential for surgical resection. The diagnostic utility of the different imaging techniques in this scenario will be discussed, including the imaging criteria for disease resectability.

For patients with non-resectable or advanced metastatic disease, treatment is usually with chemotherapy and/or radiotherapy. In this setting, conventional CT and MR imaging are most frequently used to assess the effectiveness of therapy. However, with the increasing use of novel therapeutics, including therapies that modify the stromal component of pancreatic cancers and immunotherapies, functional imaging and PET imaging are increasingly used to assess treatment response. Techniques that have been applied in this setting include CT perfusion, diffusion-weighted MRI, dynamic contrast-enhanced MRI, MR elastography and FDG-PET/CT imaging. These techniques are now being investigated as potential response, predictive and prognostic biomarkers. Such techniques are also being evaluated for the early detection of disease relapse.

**References:**

1. Zhan HX, Xu JW, Wu D, Wu ZY, Wang L, Hu SY, Zhang GY. Neoadjuvant therapy in pancreatic cancer: a systematic review and meta-analysis of prospective studies. Cancer Med. 2017 Jun;6(6):1201-1219.

2. Noda Y, Goshima S, Kawada H, Kawai N, Miyoshi T, Matsuo M, Bae KT. Modified National Comprehensive Cancer Network Criteria for Assessing Resectability of Pancreatic Ductal Adenocarcinoma. AJR Am J Roentgenol. 2018 Jun;210(6):1252-1258.

3. Cassinotto C, Mouries A, Lafourcade JP, Terrebonne E, Belleannée G, Blanc JF, Lapuyade B, Vendrely V, Laurent C, Chiche L, Wagner T, Sa-Cunha A, Gaye D, Trillaud H, Laurent F, Montaudon M. Locally advanced pancreatic adenocarcinoma: reassessment of response with CT after neoadjuvant chemotherapy and radiation therapy. Radiology. 2014 Oct;273(1):108-16.

4. Amer AM, Zaid M, Chaudhury B, Elganainy D, Lee Y, Wilke CT, Cloyd J, Wang H, Maitra A, Wolff RA, Varadhachary G, Overman MJ, Lee JE, Fleming JB, Tzeng CW, Katz MH, Holliday EB, Krishnan S, Minsky BD, Herman JM, Taniguchi CM, Das P, Crane CH, Le O, Bhosale P, Tamm EP, Koay EJ. Imaging-based biomarkers: Changes in the tumor interface of pancreatic ductal adenocarcinoma on computed tomography scans indicate response to cytotoxic therapy. Cancer. 2018 Apr 15;124(8):1701-1709.

5. Akisik MF, Sandrasegaran K, Bu G, Lin C, Hutchins GD, Chiorean EG. Pancreatic cancer: utility of dynamic contrast-enhanced MR imaging in assessment of antiangiogenic therapy. Radiology. 2010 Aug;256(2):441-9.

#### A19 Ablation of pancreatic cancer

##### Mirko D’Onofrio

###### Department of Radiology, GB Rossi University Hospital, University of Verona, Verona, Italy

Pancreatic ductal adenocarcinoma is a highly aggressive tumor with an overall 5-year survival rate of less than 5%. Prognosis and treatment depend on whether the tumor is resectable or not, which mostly depends on how quickly the diagnosis is made. Chemotherapy and radiotherapy can be both used in cases of nonresectable pancreatic cancer. In cases of pancreatic neoplasm that is locally advanced, non-resectable, but non-metastatic, it is possible to apply percutaneous treatments that are able to induce tumor cytoreduction. To describe the multiple currently available treatment techniques (radiofrequency ablation, microwave ablation, cryoablation, and irreversible electroporation), their results, and their possible complications.

This abstract was taken from a previous publication [1].

References

1. D’Onofrio M, Ciaravino V, De Robertis R, Barbi E, Salvia R, Girelli R, Paiella S, Gasparini C, Cardobi N, Bassi C. Percutaneous ablation of pancreatic cancer. *World journal of gastroenterology*. 2016 Nov 28;22(44):9661.

### 11:00 – 11:30 Keynote Lecture 2

#### K2 PSMA molecular imaging and theranostic

##### K. Herrmann

###### Dept. of Nuclear Medicine, Universitätsklinikum Essen, Essen, Germany

Recent introduction of highly specific ligands binding to the prostate specific membrane antigen labelled with either diagnostic or therapeutic radionuclides have boosted the field of prostate cancer imaging and therapy (Eiber et al., doi: 10.2967/jnumed.117.198119). Aim of this presentation is to give an overview over this quickly developing field, helping to understand which clinical data is already available and what is needed to achieve FDA- and EMA-approval. For diagnostic imaging recent meta-analyses (Perera et al., doi: 10.1016/j.eururo.2016.06.021; Corfield et al., doi: 10.1007/s00345-018-2182-1) as well as the summary of the recent EANM FOCUS meeting in Valencia (Fanti et al.) will be taken into account. For the theranostic part the current available body of evidence will be summarized and presented (Rahbar et al., 10.2967/jnumed.116.183194; Hofman et al., 10.1016/S1470-2045(18)30198-0). Of course, also the recently started prospective VISION trial will be touched upon. Moreover, this presentation also dares an outlook into what could be the next steps to enhance the efficacy of currently clinically translated approaches. In summary, this presentation addresses one of the most exciting and quickly developing fields in cancer imaging and therapy.

Key References:

Corfield et al., doi: 10.1007/s00345-018-2182-1

Eiber et al., doi: 10.2967/jnumed.117.198119

Fanti et al., in review

Hofman et al., 10.1016/S1470-2045(18)30198-0

Perera et al., doi: 10.1016/j.eururo.2016.06.021

Rahbar et al., 10.2967/jnumed.116.183194

### 11:30 – 13:00 Liver Tumours I

#### A20 PRETEXT classification for paediatric liver tumours

##### AJ Towbin

###### Cincinnati Children’s Hospital, Cincinnati, OH, USA

The PRETEXT classification system has been used as a means to assess paediatric liver tumours in cooperative group trials since its inception. In 2017, paediatric radiologists, oncologists, and surgeons from Europe, Japan, and the United States met to update the classification system via a consensus session. The purpose of this update was to bridge variations across the cooperative groups, standardize terminology, and simplify the methodology for determining vascular involvement. In this lecture, attendees will learn how to apply the 2017 PRETEXT classification system to paediatric liver tumours, how to define the different annotation factors, and how this classification system has changed from prior versions.

Reference:

Towbin AJ, Meyers RL, Woodley H, Miyazaki O, Weldon CB, Morland B, Hiyama E, Czauderna P, Roebuck DJ, Tiao GM. 2017 PRETEXT: radiologic staging system for primary hepatic malignancies of childhood revised for the Paediatric Hepatic International Tumour Trial (PHITT). Pediatr Radiol. 2018 Apr;48(4):536-554. doi: 10.1007/s00247-018-4078-z. Epub 2018 Feb 9.

#### A21 LI-RADS

##### Thorsten Persigehl (thorsten.persigehl@uk-koeln.de)

###### University Hospital Cologne, Cologne, Germany

The Liver Imaging Reporting and Data System (LI-RADS®) was created by the American College of Radiology (ACR) to standardize the reporting and data collection for hepatocellular carcinoma (HCC) [1]. LI-RADS® allows a characterization of liver findings in patients with risk for HCC. These imaging criteria aim to apply a consistent terminology, reduce imaging interpretation variability and errors, and enhance communication with referring clinicians.

LI-RADS® was recently updated in 2017 including a modified diagnostic algorithm for CT/MRI, a recommendation for the use of hepatobiliary contrast agents (e.g. Gd-EOB-DTPA), a standardized reporting guidance, a new treatment response assessment algorithm, and a basic management guidance.

The focus of this talk is the presentation of LI-RADS® v2017 for characterization and monitoring of hepatic lesions in CT and MRI. Moreover, structured reporting in the daily routine will be discussed.

Reference:

1. https://www.acr.org/Clinical-Resources/Reporting-and-Data-Systems/LI-RADS/CT-MRI-LI-RADS-v2017

#### A22 Response assessment after local therapy

##### Bernhard Gebauer (bernhard.gebauer@charite.de)

###### Charité Universitätsmedizin Berlin, Radiological Clinic, Charité Campus Virchow-Klinikum, Augustenburger Platz 1, Berlin, Germany

The assessment of response after localized tumor treatment differs from response assessment after systemic tumor treatment. First not all tumor manifestations in the body have been treated, second hemorrhages within the treated tumor could be misinterpreted as contrast enhancement and third tumor diameter and volume could even increase due to treatment of a safety margin of healthy tissue around the tumor.

Additionally local morphological tissue changes depend on the used technology (thermal ablation (RFA, MWA, cyoablation), biomechanical ablation (irreversible electroporation (IRE), chemoembolisation (TACE), radioembolisation (RE, SIRT), brachytherapy or external radiation (SBRT)), the imaging modality (CT, MRI or PET-CT) and the treated organ (liver, lung, kidney, brain or bone).

In the liver in arterial enhancing tumors (e.g. hepatocellular cancer or metastasis from neuroendocrine tumors) the concept of absence of arterial enhancement after thermal ablation or chemoembolisation is an accepted concept of local response assessment (1, 2, 11). Under these circumstances the modified RECIST criteria are widely accepted for local tumor control. These criteria only taking into account the arterial enhancing portion of the tumor. In radioembolisation (RE, SIRT) of hepatic malignancies the assessment of response differs from these mRECIST criteria, because RE/SIRT is a radiation therapy and similar to external beam radiation RE/SIRT does not alter arterial tumor enhancement (9, 10, 15). For response assessment after RE/SIRT no established criteria exist, but there is increasing interest in functional imaging, especially with MRI using DWI, perfusion imaging and hepatocyte-specific contrast (gadoxetic acid).

After thermal ablation in renal cell cancer hemorrhagic ablation areal could be misinterpreted as a tumor recurrence-suspect arterial enhancement, so a native scan in MRI and CT is essential to quantify the extent of hemorrhagic tissue after ablation, in MRI additional subtraction images after intravenous contrast enhancement could simplify the detection of residual tumor or local tumor recurrence after thermal ablation.

In thermal ablation of lung tumor with CT the most frequent early imaging appearance is ground-glass opacity (GGO) of the ablated area. This early GGOs should extent more than 5 mm beyond the tumor margins (6) to ensure complete tumor ablation. After this early phase the ablated area appears as a well-demarcated homogeneous dense opacity in CT. In the further follow-up imaging this area will show a relatively slow involution with constant decrease of size. Additionally different CT shapes (atelectasis, cavitation, disappearance, fibrosis or nodular) of the treated area have been described and patter changes from one category into another have been described. To detect tumor recurrences after pulmonal ablation in CT is often difficult, but relies mostly on morphological changes of the ablation area. PET-CT is an alternative to detect tumor recurrancies after thermal ablation, but should not be used within the first 3 months after ablation, because inflammation could mimic active tumor.

Literature:

1. Adam SZ, Miller FH. Imaging of the Liver Following Interventional Therapy for Hepatic Neoplasms. Radiol Clin North Am. 2015;53(5):1061-76.

2. Bouda D, Lagadec M, Alba CG, Barrau V, Dioguardi Burgio M, Moussa N, Vilgrain V, Ronot M. Imaging review of hepatocellular carcinoma after thermal ablation: The good, the bad, and the ugly. Journal of magnetic resonance imaging : JMRI. 2016;44(5):1070-90.

3. Choi YW, Munden RF, Erasmus JJ, Park KJ, Chung WK, Jeon SC, Park CK. Effects of radiation therapy on the lung: radiologic appearances and differential diagnosis. Radiographics. 2004;24(4):985-97; discussion 98.

4. Coche E. Evaluation of lung tumor response to therapy: Current and emerging techniques. Diagn Interv Imaging. 2016;97(10):1053-65.

5. Covey AM, Hussain SM. Liver-Directed Therapy for Hepatocellular Carcinoma: An Overview of Techniques, Outcomes, and Posttreatment Imaging Findings. AJR Am J Roentgenol. 2017;209(1):67-76.

6. de Baere T, Tselikas L, Gravel G, Deschamps F. Lung ablation: Best practice/results/response assessment/role alongside other ablative therapies. Clin Radiol. 2017;72(8):657-64.

7. Gervais DA, Kalva S, Thabet A. Percutaneous image-guided therapy of intra-abdominal malignancy: imaging evaluation of treatment response. Abdom Imaging. 2009;34(5):593-609.

8. Ghaye B, Wanet M, El Hajjam M. Imaging after radiation therapy of thoracic tumors. Diagn Interv Imaging. 2016;97(10):1037-52.

9. Haddad MM, Merrell KW, Hallemeier CL, Johnson GB, Mounajjed T, Olivier KR, Fidler JL, Venkatesh SK. Stereotactic body radiation therapy of liver tumors: post-treatment appearances and evaluation of treatment response: a pictorial review. Abdom Radiol (NY). 2016;41(10):2061-77.

10. Kellock T, Liang T, Harris A, Schellenberg D, Ma R, Ho S, Yap WW. Stereotactic body radiation therapy (SBRT) for hepatocellular carcinoma: imaging evaluation post treatment. Br J Radiol. 2018;91(1085):20170118.

11. Lencioni R, Llovet JM. Modified RECIST (mRECIST) assessment for hepatocellular carcinoma. Seminars in liver disease. 2010;30(1):52-60.

12. Mattonen SA, Palma DA, Haasbeek CJ, Senan S, Ward AD. Distinguishing radiation fibrosis from tumour recurrence after stereotactic ablative radiotherapy (SABR) for lung cancer: a quantitative analysis of CT density changes. Acta Oncol. 2013;52(5):910-8.

13. Minami Y, Nishida N, Kudo M. Therapeutic response assessment of RFA for HCC: contrast-enhanced US, CT and MRI. World journal of gastroenterology : WJG. 2014;20(15):4160-6.

14. Nour-Eldin NE, Naguib NN, Tawfik AM, Gruber-Rouh T, Zangos S, Vogl TJ. CT volumetric assessment of pulmonary neoplasms after radiofrequency ablation: when to consider a second intervention? J Vasc Interv Radiol. 2014;25(3):347-54.

15. Riaz A, Miller FH, Kulik LM, Nikolaidis P, Yaghmai V, Lewandowski RJ, Mulcahy MF, Ryu RK, Sato KT, Gupta R, Wang E, Baker T, Abecassis M, Benson AB, 3rd, Nemcek AA, Jr., Omary R, Salem R. Imaging response in the primary index lesion and clinical outcomes following transarterial locoregional therapy for hepatocellular carcinoma. JAMA : the journal of the American Medical Association. 2010;303(11):1062-9.

16. https://www.acr.org/-/media/ACR/Files/RADS/LI-RADS/LIRADS_2017_Core.pdf?la=en

### 11:30 – 13:00 Neurooncology

#### A23 Major changes in the new 2016 WHO central nervous system tumours classification: what the radiologists need to know

##### Giovanni Morana^1^, Maria Luisa Garrè^2^, Andrea Rossi^1^

###### ^1^Neuroradiology Unit, Istituto Giannina Gaslini, Genoa, GE, Italy; ^2^Neuro-oncology Unit, Istituto Giannina Gaslini, Genoa, GE, Italy

####### **Correspondence:**Giovanni Morana (giovannimorana@gaslini.org)

The 2016 World Health Organization (WHO) classification of tumours of the Central Nervous System (CNS) officially represents an update of the 2007 CNS WHO classification.

The current classification introduces the concept of "integrated” or “layered” diagnoses based on a combination of both traditional histological criteria and molecular biomarkers, with the intended goals of improving diagnostic accuracy and patient management. Under the new WHO classification scheme, molecular and genetic data supplement rather than displace histologic classification, even though the rule “molecular beats histology” was introduced [1-3].

The result is a major restructuring in many of the brain tumour categories. Major changes include restructuring of diffuse gliomas, medulloblastomas and other embryonal tumours, incorporation of a genetically defined ependymoma variant, and recognition of newly defined entities, variants and patterns, including diffuse leptomeningeal glioneuronal tumour and multinodular and vacuolating tumour of the cerebrum. Several previously recognized brain tumour diagnoses, such as oligoastrocytoma, primitive neuroectodermal tumour, and gliomatosis cerebri, were redefined or eliminated altogether [4,5].

The glioma category has been significantly reorganized, with several infiltrating gliomas in children and adults now defined by genetic features for the first time.

Review of the major changes to the WHO brain tumour classification system that are most pertinent to radiologists is the focus of the present work with special emphasis on gliomas.

**References**

1. Louis DN, Perry A, Reifenberger G, von Deimling A, Figarella-Branger D, Cavenee WK, et al. The 2016 World Health Organization Classification of Tumors of the Central Nervous System: a summary. Acta Neuropathol. 2016, 131:803-20.
2. Johnson DR, Guerin JB, Giannini C, Morris JM, Eckel LJ, Kaufmann TJ. 2016 Updates to the WHO Brain Tumor Classification System: What the Radiologist Needs to Know. Radiographics 2017, 37:2164-2180.
3. Banan R, Hartmann C. The new WHO 2016 classification of brain tumors-what neurosurgeons need to know. Acta Neurochir (Wien). 2017, 159:403-418.
4. van den Bent MJ, Weller M, Wen PY, Kros JM, Aldape K, Chang S. A clinical perspective on the 2016 WHO brain tumor classification and routine molecular diagnostics. Neuro Oncol. 2017, 1;19:614-624.
5. Reifenberger G, Wirsching HG, Knobbe-Thomsen CB, Weller M. Advances in the molecular genetics of gliomas - implications for classification and therapy. Nat Rev Clin Oncol. 2017, 14:434-452

#### A24 New molecular classification of medulloblastoma in children

##### Thierry A.G.M. Huisman

###### Division of Pediatric Radiology and Pediatric Neuroradiology, Johns Hopkins Hospital, Baltimore, MD, USA

Medulloblastoma is the most common malignant pediatric brain tumor, and is a rapidly growing, invasive neoplasm that accounts for 40% of childhood tumors in the posterior fossa. In the United States, the overall incidence of medulloblastoma is around 1.5 per million people for the past 40 years, with a marginal male to female preponderance of approximately 1.5:1.4. Although the treatment and mortality of medulloblastomas in children improved significantly in the last decades, this group of pediatric brain tumors remains an important cause of severe morbidity in children.

Not until the recent past, medulloblastomas were classified based primarily on histopathology which included types such as classic medulloblastoma, desmoplastic/nodular, medulloblastoma with extensive nodularity (MBEN), large cell, and anaplastic medulloblastoma. Recently, a novel classification of medulloblastomas based on the underlying genetic mutation has been introduced and includes 4 different subgroups medulloblastomas: 1) wingless (WNT) group, 2) sonic hedgehog (SHH) group, 3) group 3, and 4) group 4. The differentiation between genetic subgroups of medulloblastomas has shown to be beneficial for therapy decision (different subgroups may benefit from different ,specific therapies) and influence long-term morbidity. More recent work aims to further refine subtype classification based on similarity network fusion and genome-wide DNA methylation and gene expression data, resulting in a total of 12 subtypes.

Despite the molecular advances and use of immunohistochemistry markers, logistical challenges remain including costs, lack of widespread availability and practicality to screen all patients with medulloblastoma in order to implement a subtype-specific treatment approach^.^ MR imaging (MRI) is universally used as a standard protocol in all patients with brain tumors and remains the primary method for diagnosis, assessment of therapy efficacy, and surveillance for these tumors.

Conventional MRI is useful in identifying and evaluating the different histopathological types of medulloblastomas and may suggest specific molecular subtypes based on morphological pattern, location, extent and contrast enhancement.

Advanced MR imaging such as diffusion-weighted imaging (DWI) and diffusion tensor imaging (DTI) have been shown to provide important additional information about the biology of brain tumors which can be utilized for medulloblastoma imaging, for example the correlation of higher mean apparent diffusion coefficient (ADC) values which were found to be more characteristic of large-cell/anaplastic medulloblastoma than classic medulloblastoma. Preliminary results showed that apparent diffusion coefficient analysis of the enhancing solid component of the tumor may contribute to the pre-operative molecular classification of pediatric medulloblastomas.

### Afternoon Session: 14:00 – 15:30 Lung Cancer

#### A25 Stratification of Lung Nodules and the New Fleischner Guidelines

##### Theresa C. McLoud (mcloud.theresa@mgh.harvard.edu)

###### Department of Radiology, Massachusetts General Hospital/Harvard Medical School, 55 Fruit Street, Founders 210, Boston, MA, USA

**Stratification of Lung Nodules**

Stratification of lung nodules for malignancy depends on a number of risk factors. They include both clinical and radiologic. Clinical risk factors include older age, history of smoking, history of extrathoracic cancer, family history of cancer and emphysema. Classic CT characteristics of lung cancer include the size of the nodule, i.e. greater than 8 mm, border characteristics, density, solidity, growth, and location. Presumptive benign diagnoses can only be made when a two-year stability has been established or calcifications are identified in a benign pattern. The presence of fat within a nodule or perifissural location are strong indications of benignity. Thin section (1.5 to 2 mm) CT is highly recommended for indeterminant solitary pulmonary nodules. Most nodules particularly small nodules are indeterminant and follow-up is the usual management.

The new Fleischner Guidelines published in 2017 provide a template for the management and follow-up of incidental nodules both solid and subsolid. These guidelines incorporate several changes from the original guidelines published in 2005. For solid nodules, the minimum threshold size for routine follow-up has been increased and fewer follow-up examinations are recommended for stable nodules. For subsolid nodules a longer period is recommended before initial follow-up and the total length of follow-up has been extended to 5 years. The guidelines are applied only to incidental nodules and do not apply to patients younger than 35 years of age and immunocompromised patients or patients with cancer. For lung cancer screening adherence to the existing American College of Radiology lung CT screening reporting and data system (Lung / RADS) guidelines is recommended.

References:

1. MacMahon H, Naidich P, Goo JM et al. Guidelines for management of incidental nodules detected on CT. From the Fleischner Society 2017. Radiology. 2017;284:228-243.
2. Webb WR. Radiologic evaluation of the solitary pulmonary nodule. AJR. 1990;154(4):701-708.
3. Erasmus JJ, Connolly JE, McAdams HP, et al. Solitary pulmonary nodules. I. Morphologic evaluation for differentiation of benign and malignant lesions. Radiographics. 2000;20(1):43-58.
4. Gould MK, Fletcher J. Iannettoni MD, et al. Evaluation of patients with pulmonary nodules: When is it lung cancer? ACCP evidence-based clinical practice guidelines (2^nd^ edition). Chest 2007;132(3 Suppl):108S-130S.
5. Silva M. Bankier AA, Centra F, et al. Longitudinal evolution of incidentally detected solitary pure ground-glass nodules on CT: Relation to clinical metrics. Diagn Interv Radiol. 2015;21(5):385-390.

#### A26 Lung cancer: unusual presentations

##### S. Diederich (stefan.diederich@vkkd-kliniken.de)

###### Department of Diagnostic and Interventional Radiology, Marien Hospital, Düsseldorf, Germany

**Common presentations of lung cancer**

Lung cancer most commonly presents as a pulmonary nodule or mass. The lesion is usually lobulated and/or spiculated due to lymphangitic spread, desmoplastic reaction or distal subsegmental collaps. If it occludes a bronchus a poststenotic atelectasis or pneumonia may be present.

**Atelectasis**

In rare cases endobronchial tumors may not present as nodule or mass even at CT but only with atelectasis or obstructive pneumonia. Histologically these centrally located endobronchial tumors most commonly represent squamous cell carcinoma. Atelectasis with no extraluminal mass is also commonly found in carcinoid / neuroendocrine carcinoma

**Lymphadenopathy with no lung lesion**

In aggressive lung cancer, particularly small cell lung cancer, imaging may show extensive hilar or mediastinal lymphadenopathy with no discernible lung lesion – probably either due to a purely endobronchial primary tumour or coalescence of a small pulmonary tumour with large hilar masses. Differential diagnosis in these cases is mainly Non-Hodgkin lymphoma, which may also present with extensive hilar and mediastinal lymphadenopathy.

**Diffuse consolidation**

Diffuse consolidation is typical for adenocarcinoma with lepidic growth. In a single lesion this may mimick a focal inflammatory lesion such as focal pneumonia or granulomatosis with polyangiitis (Wegener´s disease). In more diffuse disease infiltration of larger parts of a segment or lobe it may mimick pneumonia or other infiltrative lung disease. The pattern may be multifocal with involvement of other ipsilateral or contralateral lobes.

**Interstitial lung disease**

Occasionally lung cancer may be associated with extensive lymphangitic spread. If the primary pulmonary is small the disease may appear as a diffuse interstitial lung disease mimicking sarcoidosis or other DILD.

**References**

Bhatia K and Ellis S. Unusual lung tumours: an illustrated review of CT features suggestive of this diagnosis. Cancer Imaging 2006; 6: 72–82

Lee KS et al. Bronchioloalveolar carcinoma: clinical, histopathologic, and radiologic findings. Radiographics 1997; 17: 1345-1357

Rosado-de-Christenson ML et al. Bronchogenic carcinoma: radiologic-pathologic correlation. Radiographics 1994; 14: 429-446

Snoeckx A et al. Wolf in Sheep's Clothing: Primary Lung Cancer Mimicking Benign Entities. Lung Cancer. 2017; 112: 109-117

#### A27 Improving lung cancer screening using radiomics

##### F. Prior (FWPrior@uams.edu)

###### Department of Biomedical Informatics, University of Arkansas for Medical Sciences, Little Rock, Arkansas, USA

Lung cancer is the leading cause of cancer deaths and the second most common cancer in both men and women in the United States. Early detection is key to early intervention leading to improved survival. Low-dose CT screening has been shown to significantly reduce mortality but suffers from false positive rates as high as 58%. This not only increases the cost for unnecessary diagnostic tests, but also causes unnecessary anxiety for patients and their families. Quantitative image analysis coupled with deep learning techniques hold the potential to reduce this false positive rate.

Numerous computer-aided approaches have been developed for chest image analysis in the past fifty years. Most computational approaches to date focus on detecting and characterizing lung nodules. This dependency on predefined objects of interest requires detection and segmentation steps that are difficult to automate and limit the applicability of many approaches for automated screening. A recent literature review concludes that deep learning is currently becoming the dominant approach in the field and has produced very promising results.

Our group has chosen to employ a deep learning technique and to focus on risk prediction at the patient level, taking into account information from the whole lung. Our studies indicate that deep learning approaches can significantly reduce the false positive rate in lung cancer screening and have the potential to produce a fully automated screening assay.

1. Siegel R, Miller K, Jemal A. Cancer statistics, 2018. CA Cancer Journal of Clinicians. 68: 7-30.
2. Aberle DR, Adams AM, Berg CD, Black WC, Clapp JD, et al. (2011) Reduced lung-cancer mortality with low-dose computed tomographic screening. N Engl J Med 365: 395-409.
3. Kinsinger LS, Anderson C, Kim J, Larson M, Chan SH, King HA, Rice KL, Slatore CG, Tanner NT, Pittman K, Monte RJ, McNeil RB, Grubber JM, Kelley MJ, Provenzale D, Datta SK, Sperber NS, Barnes LK, Abbott DH, Sims KJ, Whitley RL, Wu RR, Jackson GL. Implementation of Lung Cancer Screening in the Veterans Health Administration. *JAMA Intern Med.* 177(3):399–406, 2017.
4. Bram van Ginneken. Fifty years of computer analysis in chest imaging: rule-based, machine learning, deep learning, Radiol Phys Technol. 2017; 10(1): 23–32.
5. Shih-Chung Benedict Lo; Jyh-Shyan Lin; Matthew T. Freedman; Seong Ki Mun. Computer-assisted diagnosis of lung nodule detection using artificial convolution neural network, Proceedings Volume 1898, Medical Imaging 1993: Image Processing; (1993); doi: 10.1117/12.154572

### 14:30 – 15:30 Imaging Complications of Cancer Therapy

#### A28 Acute abdominal complications of cancer therapy in children

##### AMJB Smets, EE Deurloo

###### Academic Medical Center, Amsterdam, The Netherlands

####### **Correspondence:**AMJB Smets: (a.m.smets@amc.uva.nl)

Depending on the type of malignancy, children with cancer are treated with different types of (chemo)therapy, all coming with a set of side effects and complication risks. The acute complications associated to therapy can be divided into those related to the effect they may have on the immune status of the patient and those directly related to therapeutic agent toxicity. Signs and symptoms of acute toxicity are myelosuppression, alopecia, nausea and emesis, mucositis, liver function disturbances, and allergic reactions. Immunosuppressive therapy and the disruption of the muco-cutaneous integrity by local tumor invasion, insertion of foreign bodies such as vascular catheters, surgery, radiotherapy and cytotoxic chemotherapy renders patients vulnerable to infection. During the course of their treatment and when a complication is clinically suspected, children with cancer are often referred for imaging studies. Awareness of these possible complications and their presentation allows radiologists to recognize them. In this lecture we will focus on abdominal complications of cancer therapy. The most common types in children will be discussed.

References:

1. Chavhan GB, Babyn PS, Nathan PC, Kaste SC. Imaging of acute and subacute toxicities of cancer therapy in children. Pediatric radiology. 2016;46(1):9-20; quiz 6-8.
2. Khoury NJ, Kanj V, Abboud M, Muwakkit S, Birjawi GA, Haddad MC. Abdominal complications of chemotherapy in pediatric malignancies: imaging findings. Clinical imaging. 2009;33(4):253-60.

#### A29 Complications of central venous lines and ports

##### Bernhard Gebauer (bernhard.gebauer@charite.de)

###### Charité Universitätsmedizin Berlin, Radiological Clinic, Charité Campus Virchow-Klinikum, Augustenburger Platz 1, Berlin, Germany

Central venous catheters and lines play an important role in anticancer treatment and a safe and reliable central venous position of catheter tip is important to prevent complications during treatment. Radiological methods are most frequently used to detect catheter complications, especially conventional x-ray, computed tomography and contrast angiography (sometimes linogram called). Ultrasound and MRI could be additional tolls in selected cases. Catheter history including symptoms during catheter usage, ability to withdraw blood over the catheter and local skin changes are of particular importance in the diagnosis of catheter complications.

Many catheter complications could be corrected, specially interventional radiology, so in many cases a catheter removal and re-implantation could be avoided.

Typical catheter complications are, catheter-related infection, catheter dysfunction , catheter occlusion & thrombosis, catheter migration, venous thrombosis, venous perforation, venous stenosis, catheter fracture or pinch-off, extravasation, catheter disconnection, fibrin-sheath, cardiac perforation, cardiac arrhythmias and skin erosion at entry side.

Literature:

1. Hentrich M, Schalk E, Schmidt-Hieber M, Chaberny I, Mousset S, Buchheidt D, Ruhnke M, Penack O, Salwender H, Wolf HH, Christopeit M, Neumann S, Maschmeyer G, Karthaus M, Infectious Diseases Working Party of the German Society of H, Medical O. Central venous catheter-related infections in hematology and oncology: 2012 updated guidelines on diagnosis, management and prevention by the Infectious Diseases Working Party of the German Society of Hematology and Medical Oncology. Ann Oncol. 2014;25(5):936-47.

2. Gaddh M, Antun A, Yamada K, Gupta P, Tran H, El Rassi F, Kim HS, Khoury HJ. Venous access catheter-related thrombosis in patients with cancer. Leukemia & lymphoma. 2014;55(3):501-8.

3. Schiffer CA, Mangu PB, Wade JC, Camp-Sorrell D, Cope DG, El-Rayes BF, Gorman M, Ligibel J, Mansfield P, Levine M. Central venous catheter care for the patient with cancer: American Society of Clinical Oncology clinical practice guideline. Journal of clinical oncology : official journal of the American Society of Clinical Oncology. 2013;31(10):1357-70.

4. Patel IJ, Davidson JC, Nikolic B, Salazar GM, Schwartzberg MS, Walker TG, Saad WA. Consensus guidelines for periprocedural management of coagulation status and hemostasis risk in percutaneous image-guided interventions. J Vasc Interv Radiol. 2012;23(6):727-36.

5. Surgeons ACo. Revised statement on recommendations for use of real-time ultrasound guidance forplacement of central venous catheters. Bull Am Coll Surg. 2011;96(2):36-7.

6. O'Grady NP, Alexander M, Burns LA, Dellinger EP, Garland J, Heard SO, Lipsett PA, Masur H, Mermel LA, Pearson ML, Raad, II, Randolph AG, Rupp ME, Saint S. Guidelines for the prevention of intravascular catheter-related infections. Clin Infect Dis. 2011;52(9):e162-93.

7. Patel RY, Friedman A, Shams JN, Silberzweig JE. Central venous catheter tip malposition. J Med Imaging Radiat Oncol. 2010;54(1):35-42.

8. McCann M, Einarsdottir H, Van Waeleghem JP, Murphy F, Sedgewick J. Vascular access management III: central venous catheters. J Ren Care. 2010;36(1):25-33.

9. Dariushnia SR, Wallace MJ, Siddiqi NH, Towbin RB, Wojak JC, Kundu S, Cardella JF. Quality improvement guidelines for central venous access. J Vasc Interv Radiol. 2010;21(7):976-81.

10. Gebauer B, Teichgraber UM, Werk M, Beck A, Wagner HJ. Sonographically guided venous puncture and fluoroscopically guided placement of tunneled, large-bore central venous catheters for bone marrow transplantation-high success rates and low complication rates. Support Care Cancer. 2008;16(8):897-904.

11. Gebauer B, Teichgraber UK, Podrabsky P, Werk M, Hanninen EL, Felix R. Radiological interventions for correction of central venous port catheter migrations. Cardiovasc Intervent Radiol. 2007;30(2):216-21.

#### A30 Imaging of Treatment Complication in the Pelvis

##### Aslam Sohaib

###### Department of Imaging, Royal Marsden Hospital, Fulham Road, London, UK

As in other parts of the body, the oncological treatment of malignancies in the pelvis may be undertaken with surgery, radiotherapy, medical therapies, and other minimally invasive therapies or with a combination of interventions. The types of treatments depend on the cancer type, stage and patient related factors. In all these imaging plays a central role in assessment of cancer therapies. This is most often looking for response or in the detection of recurrent disease and less commonly for treatment related complications.

In the interpretation of imaging following cancer therapies it is important for the radiologist to understand the malignancy, the treatment administered and to be aware of the spectrum of finding following the different types of treatments. This will allow the assessment of the therapy given to the patient but also to look for complication related to the treatment.

For surgical procedures common complications apply to all surgical operations e.g. haemorrhage. However to appreciate other specific complication the radiologist needs to know when and the details of procedures that have been performed e.g. pelvic exenteration or bladder reconstructions. Similar with minimally invasive therapies, the radiologist needs to have knowledge of the procedure and its likely outcomes eg HIFU treatment for prostate cancer.

Radiotherapy is commonly used in treating pelvic malignancies. The range and manifestation of radiotherapy on the pelvic viscera depends on the type and dose of radiotherapy and when it was administered. Changes in the target organ as well as other adjacent organs may be seen depending on the types of radiation treatment.

The combination of appropriate clinical information, imaging, including correlation with previous findings will allow for differentiation between therapy-induced complications and tumour recurrence or tumour related complication in the majority of cases. Therefore, imaging plays a central role in the multidisciplinary management of these patients.

**References**

1. Shah A., Sohaib S.A., Koh DM. (2015) Imaging of Complications and Toxicity Following Tumour Therapy: Pelvis and Genitourinary (Male). In: Kauczor HU., Bäuerle T. (eds) Imaging of Complications and Toxicity following Tumor Therapy. Medical Radiology. Springer, Cham

2. Forstner R., Cunha T.M. (2015) Female Pelvis: Genital Organs. In: Kauczor HU., Bäuerle T. (eds) Imaging of Complications and Toxicity following Tumor Therapy. Medical Radiology. Springer, Cham

### 16:00 – 17:30 Federation du Cancer de la Société Française de Radiologie

#### A31 Immunotherapy: a revolution in cancer care, a new challenge for imaging

##### C. Dromain

###### University Hospital Vaud, Lausanne, Switzerland

A wide range of cancer immunotherapy approaches have been developed including non-specific immune-stimulant such as cytokines (Interferon, IL2), cancer vaccines (peptide or dendritic-cell-based vaccines), adoptive T-cell therapy (TILs, CAR, TRC) and immune checkpoint inhibitors (anti CTLA-4, anti PD1 and anti PDL1). The most commonly used and intensively studied are the immune checkpoint inhibitors (ICIs). Their mechanism of action signifies a true shift in oncology where instead of targeting the tumor cells, ICIs target the immune system in order to break the cancer tolerance and stimulate the anti-tumor immune response. These new drugs have, since 2011, received marketing authorization for melanoma, lung, bladder, renal, and head and neck cancer with remarkable and long-lasting treatment response. The novel mechanism of action of these drugs, with immune and T-cell activation, lead to unusual patterns of response with presence of flare phenomenon or pseudo-progression more pronounced and more frequent than previously described responses. Pseudo-progression, that has been described in about 3-10% of patients treated using ICIs, corresponds to increase of tumor burden and/or appearance of new lesions due to Infiltration of the tumor by activated T-cells before the disease responds to treatment. To overcome the limitation of RECIST criteria to assess this specific changes in tumour burden, new criteria so-called irRC and then irRECIST were proposed. The major modification involved the inclusion of the measurements of new target lesions into disease assessments and the need of a 4-week CT re-assessment to confirm progression. More recently (2017) a consensus guideline iRECIST was developed by the RECIST working group.

#### A32 The interventional oncologist – the fourth musketeer of cancer care

##### F Cornelis, I Thomassin, F Boudghene

###### Sorbonne Université – ISCD / APHP - HUEP - Hôpital Tenon, Paris, FRANCE

Interventional Oncology (IO) is currently one of the most active medical specialties. Alongside medical oncology, surgery, and radiotherapy, IO turns out to be the fourth pillar of cancer care. The ongoing research may even expand its scope and importance worldwide.

The interventional oncologist is already involved in all stages of the patient's disease, from diagnosis to treatment. Likewise, he or she participates in the management of all related problems due to cancer during the follow-up. Therefore, the interventional oncologist has to set up a perfect organization, workflow and access to appropriate guidance tools or devices. This allows for performing the procedures safely and efficiently while being in daily practice. To achieve these goals and gain recognition, the interventional oncologist must be well-versed with the methods of the "dream team" (i.e. oncologists and surgeons), not only for determining the best-fit treatments but also for the evaluation of treatments. Besides consultation and participation in multidisciplinary team meetings, the interventional oncologist must be in close contact with his colleagues. He or she will thus have a complete appreciation of oncology, identifying each patient’s needs and finally cementing his or her place in the team effort involved in cancer care.

IO is rarely proposed as first-line treatment but rather as an alternative in fragile patients. The current recommendations exist based on the accumulated evidence of the benefits of these procedures; however, indications are neither exclusive nor definitive. It is still possible to think outside the box to enlarge them. Hence, interventional oncologists have to be aware of the promises but also the limits of their treatments. It is essential that the interventional oncologist engages himself or herself in fundamental, transversal or clinical research to develop, improve and confirm the therapeutic armamentarium of IO. To contribute to the further evolution of current guidelines, the interventional oncologist has to defend the minimal invasiveness of his techniques. They cause less pain, fewer side effects and shorter recovery times. Performing these procedures under local anesthesia or mild sedation on an outpatient basis may shorten the time required for treatment and hospitalization. It may even improve the quality of life of patients. Beyond the cost of the devices, which must evolve to be less binding, the rationale behind this is that global costs of these procedures could be less than conventional treatments. This has to be proved by setting up broad medico-economic studies. Expansion of the current indications might occur if oncologists or surgeons are involved in the process and willing to support it. But, it has to be kept in mind that the final goal of interventional oncologists is better cancer care than the mere comparison of techniques.

As cancer is a multifaceted disease, interventional oncologists can further provide innovative and effective minimally-invasive solutions to better care for the patients in a multidisciplinary team effort and thereby be recognized as the Fourth Musketeer of the cancer care.

This abstract was taken from a previously published work [1].

References

1. Cornelis FH. The interventional oncologist: The fourth musketeer of cancer care. *Diagnostic and Interventional Imaging* 2017 (Vol. 98, no. 9) 579-581.

### 16:00 – 17:30 Haematological Cancer

#### A33 PET/MRI when and how?

##### H. Chandarana

###### Department of Radiology, New York University School of Medicine, New York, NY, USA

PET imaging (PET/CT) is routinely used in evaluation of patients with various malignancies including lymphoma. Many of these patients also undergo a separate MRI examination as a problem solving tool. These PET (PET/CT) and MRI examinations are usually performed with temporal delay (at a different time and date). Introduction of hybrid PET/MR systems over the last 5 to 10 years now enables simultaneous or near simultaneous acquisition of PET and MRI information.

The role of hybrid PET/MRI is being investigated by many groups in various oncologic applications such as cancer detection, staging, and assessment of treatment response. These hybrid PET/MR systems provide not only metabolic PET information but multi-parametric MRI information (such as diffusion and perfusion weighted imaging) which is temporally and spatially correlated.

To test, validate, and clinically implement these systems and to take advantage of all of its various capabilities and functionalities we need to understand:

- different components of the system and how they have been modified from conventional PET and MRI
- how do these systems differ from each other and from conventional PET and MRI
- how to optimize protocols and implement them to answer clinically relevant questions.

We will discuss:

1. System and workflow considerations
2. Current and potential clinical indications for oncologic imaging
3. Limitations of PET/MR systems including in osseous and lung parenchymal imaging

Citations:

1. Parikh N, Friedman KP, Shah SN, Chandarana H. Practical guide for implementing hybrid PET/MR clinical service: lessons learned from our experience. Abdom Imaging. 2015 Aug;40(6):1366-73.
2. Delso G, Fürst S, Jakoby B, et al. Performance measurements of the Siemens mMR integrated whole-body PET/MR scanner. J Nucl Med. 2011 Dec;52(12):1914-22.
3. Paulus DH, Quick HH, Geppert C,et al. Whole-Body PET/MR Imaging: Quantitative Evaluation of a Novel Model-Based MR Attenuation Correction Method Including Bone. J Nucl Med. 2015 Jul;56(7):1061-6.
4. Martinez-Möller A, Eiber M, Nekolla SG, et al. Workflow and scan protocol considerations for integrated whole-body PET/MRI in oncology. J Nucl Med. 2012 Sep;53(9):1415-26.
5. Chandarana H, Heacock L, Rakheja R, et al. Pulmonary nodules in patients with primary malignancy: comparison of hybrid PET/MR and PET/CT imaging. Radiology. 2013 Sep;268(3):874-81.
6. Rakheja R, Chandarana H, DeMello L, et al. Correlation between standardized uptake value and apparent diffusion coefficient of neoplastic lesions evaluated with whole-body simultaneous hybrid PET/MRI. AJR Am J Roentgenol. 2013 Nov;201(5):1115-9.
7. Brandmaier P, Purz S, Bremicker K, et al. Simultaneous [18F]FDG-PET/MRI: Correlation of Apparent Diffusion Coefficient (ADC) and Standardized Uptake Value (SUV) in Primary and Recurrent Cervical Cancer. PLoS One. 2015 Nov 9;10(11).
8. Karan B, Pourbagher A, Torun N. Diffusion-weighted imaging and (18) F-fluorodeoxyglucose positron emission tomography/computed tomography in breast cancer: Correlation of the apparent diffusion coefficient and maximum standardized uptake values with prognostic factors. J Magn Reson Imaging. 2016 Jun;43(6):1434-44.
9. Heacock L, Weissbrot J, Radd R, Campbell N, Friedman K, Ponzo F, Chandarana H. Hybrid PET/MRI in evaluation of patients with lymphoma: Initial observations. AJR Am J Roentgenol. 2015; 204(4):842-8.

#### A34 Lymphoma

##### Marius E. Mayerhöfer^1,2^ (marius.mayerhoefer@meduniwien.ac.at)

###### ^1^Dept. of Biomedical Imaging and Image-guided Therapy, Medical University of Vienna, Vienna, Austria; ^2^Dept. of Radiology, Memorial Sloan Kettering Cancer Center, New York, NY, USA

Lymphomas can be divided into Hodgkin (HL) and Non-Hodgkin lymphomas (NHL) on the one hand, and aggressive and indolent lymphomas on the other hand. Imaging plays a central role for the assessment of lymphomas, not only for staging, but also for treatment response assessment.

While contrast-enhanced computed tomography (CE-CT) remains the most frequently used imaging test for lymphomas, the International Conference on Malignant Lymphoma (ICML) recommends the use of [18F]FDG-PET/CT for the vast majority of lymphomas, including the most common subtypes: HL, diffuse large-B-cell lymphoma (DLBCL), and follicular lymphoma [1]. [18F]FDG-PET/CT, which relies on the high gIucose metabolism observed in most lymphomas, is superior to CE-CT, in particular for response assessment after chemo- or immune-chemotherapy. There are two sets of rules for lymphoma response evaluation: the Lugano classification [1], and the recently published RECIL classification [2]. For FDG-avid lymphomas, both rely chiefly on the so-called 5-point Deauville score, which is based on a comparison of the lesions’ FDG uptake on PET with the FDG uptake of reference tissues, especially the liver. While both classifications distinguish between complete remission, partial remission, stable disease and progressive disease, RECIL includes “minimal response” as an additional category. Apart from this semi-quantitative evaluation, glucose metabolism on [18F]FDG-PET can also be measured quantitatively through calculation of standardized uptake values (SUV), metabolic tumor volume (MTV) and total lesion glycolysis (TLG), which may carry prognostic information [3, 4].

Apart from [18F]FDG-PET/CT, whole-body diffusion-weighted MR imaging (DWI) has been proposed as an alternative technique, providing results that are almost, though not quite, as good as those achieved with [18F]-FDG-PET/CT, both in terms of pre-therapeutic staging and treatment response assessment [5, 6]. It is based on the restriction of water movement in hypercellular tumors such as lymphomas, and is thus an indirect way of assessing cell density and size, and extracellular space tortuosity. As a radiation-free technique, it is particularly attractive for pediatric lymphoma patients, and for patients with lymphoma subtypes that are not, or only in a lower percentage of cases, FDG avid. The latter include marginal zone lymphomas (MZL, which include the MALT subtype), SLL/CLL (small lymphocytic lymphoma / chronic lymphocytic leukemia), Morbus Waldenström, and cutaneous T-cell lymphomas, for which the ICML currently does not recommend [18F]FDG-PET/CT [1]. Similar to [18F]FDG-PET, DWI has quantitative capabilities through the calculation of so-called apparent diffusion coefficients (ADC). However, compared to the SUVs on PET, ADCs tend to be less reliable, and are prone to motion artifacts.

PET/MRI has recently entered the clinical stage, and several studies have been performed in lymphoma patients. Due to the combination of the strengths of [18F]FDG-PET and DWI, and the reduced radiation exposure – 6-8 mSv for PET/MRI, as opposed to about 20 mSv for PET/CT – PET/MRI may be regarded the universal, though costly, imaging test for lymphoma, even though the actual benefit in routine clinical practice may only be small, and is histology-dependent [7]. A definitive advantage over PET/CT lies in the fact that delayed time-point PET, which improves detection of some indolent lymphomas [8], can be easily integrated into the PET/MRI work-flow.

Among novel PET radiotracers used in lymphomas, [68Ga]Ga-Pentixafor, which targets CXCR4 chemokine receptors [9], has great potential, because CXCR4, which mediates cell chemotaxis and migration, is overexpressed in lympho-proliferative diseases.

This abstract has been previously published [10].

**References**

1. Cheson B, Fisher RI, Barrington SF, Cavalli F, Schwartz LH, Zucca E, Lister TA: **Recommendations for initial evaluation, staging, and response assessment of Hodgkin and non-Hodgkin lymphoma: the Lugano classification.** *J Clin Oncol* 2014;**32**:3059-3068.

2. Younes A, Hilden P, Coiffier B, Hagenbeek A, Salles G, Wilson W, Seymour JF, Kelly K, Gribben J, Pfreunschuh M, Morschhauser F, Schoder H, Zelenetz AD, Rademaker J, Advani R, Valente N, Fortpied C, Witzig TE, Sehn LH, Engert A, Fisher RI, Zinzani PL, Federico M, Hutchings M, Bollard C, Trneny M, Elsayed YA, Tobinai K, Abramson JS, Fowler N, Goy A, Smith M, Ansell S, Kuruvilla J, Dreyling M, Thieblemont C, Little RF, Aurer I, Van Oers MHJ, Takeshita K, Gopal A, Rule S, de Vos S, Kloos I, Kaminski MS, Meignan M, Schwartz LH, Leonard JP, Schuster SJ, Seshan VE. **International Working Group consensus response evaluation criteria in lymphoma (RECIL 2017)**. *Ann Oncol*. 2017;**28**:1436-1447.

3. Ceriani L, Milan L, Martelli M, Ferreri AJM, Cascione L, Zinzani PL, Di Rocco A, Conconi A, Stathis A, Cavalli F, Bellei M, Cozens K, Porro E, Giovanella L, Johnson PW, Zucca E. **Metabolic heterogeneity on baseline 18FDG-PET/CT scan is a predictor of outcome in primary mediastinal B-cell lymphoma**. *Blood*. 2018 doi: 10.1182/blood-2018-01-826958.

4. Moskowitz AJ, Schöder H, Gavane S, Thoren KL, Fleisher M, Yahalom J, McCall SJ, Cadzin BR, Fox SY, Gerecitano J, Grewal R, Hamlin PA, Horwitz SM, Kumar A, Matasar M, Ni A, Noy A, Palomba ML, Perales MA, Portlock CS, Sauter C, Straus D, Younes A, Zelenetz AD, Moskowitz CH. **Prognostic significance of baseline metabolic tumor volume in relapsed and refractory Hodgkin lymphoma**. *Blood*. 2017;**130**:2196-2203.

5. Mayerhoefer ME, Karanikas G, Kletter K, Prosch H, Kiesewetter B, Skrabs C, Porpaczy E, Weber M, Pinker-Domenig K, Berzaczy D, Hoffmann M, Sillaber C, Jaeger U, Müllauer L, Simonitsch-Klupp I, Dolak W, Gaiger A, Ubl P, Lukas J, Raderer M: **Evaluation of diffusion-weighted MRI for pretherapeutic assessment and staging of lymphoma: results of a prospective study in 140 patients.** *Clin Cancer Res* 2014;**20**:2984-2993.

6. Mayerhoefer ME, Karanikas G, Kletter K, Prosch H, Kiesewetter B, Skrabs C, Porpaczy E, Weber M, Knogler T, Sillaber C, Jaeger U, Simonitsch-Klupp I, Ubl P, Muellauer L Dolak W, Lukas J, Raderer M: **Evaluation of diffusion-weighted magnetic resonance imaging for follow-up and treatment response assessment of lymphoma: results of an 18F-FDG-PET/CT-controlled prospective study in 64 patients.** *Clin Cancer Res* 2015;**21**:2506-2513.

7. Giraudo G, Raderer M, Karanikas G, Weber M, Kiesewetter B, Dolak W, Simonitsch-Klupp I, Mayerhoefer ME: **[18F]-FDG-PET/MR in lymphoma: comparison with [18F]-FDG-PET/CT and with the addition of diffusion-weighted MRI**. *Invest Radiol* 2016;**51**:163-169.

8. Mayerhoefer ME, Giraudo C, Senn D, Hartenbach M, Weber M, Rausch I, Kiesewetter B, Herold CJ, Hacker M, Pones M, Simonitsch-Klupp I, Müllauer L, Dolak W, Lukas J, Raderer M: **Does delayed-time-point imaging improve F-18-FDG-PET in patients with MALT lymphoma? Observations in a series of 13 patients**. *Clin Nucl Med* 2016;**41**:101-10

9. Herrmann K, Lapa C, Wester HJ, Schottelius M, Schiepers C, Eberlein U, Bluemel C, Keller U, Knop S, Kropf S, Schirbel A, Buck AK, Lassmann M: **Biodistribution and radiation dosimetry for the chemokine receptor CXCR4-targeting probe 68Ga-pentixafor**. *J Nucl Med* 2015;**56**:410-416.

10. Mayerhöfer ME. Proceedings of the International Cancer Imaging Society (ICIS) 17th Annual Teaching Course. In *Cancer Imaging* 2017 Sept (Vol. 17, No. 1, p. 24). BioMed Central.

#### A35 Pediatric Lymphoma

##### Stephan D. Voss

###### Department of Radiology, Boston Children's Hospital, Harvard Medical School, Boston, MA USA

Pediatric Lymphoma, both Hodgkin and non-Hodgkin subtypes, has served as the model in pediatric oncology for the use of functional imaging to guide response-based treatment strategies. Over the past 10 years the incorporation of functional imaging, mainly FDG-PET, into standard response assessment algorithms has allowed response-based protocols to be developed with the goal of reducing late affects related to chemo- and radiation therapy. The purpose of this presentation is to provide an overview of the imaging techniques and response definitions used in Pediatric Lymphoma. We will review both Hodgkin and non-Hodgkin lymphoma, understanding that while many similarities exist, important differences are also notable. The use of PET/MRI will be discussed along with a review of quantitative techniques to refine definitions of FDG-PET response. Finally we will conclude with a brief discussion of surveillance imaging and new tracers and techniques in development for imaging Pediatric Lymphoma.

**References:**

Barrington, S.F. and Kluge, R. FDG PET for therapy monitoring in Hodgkin and non-Hodgkin lymphomas. Eur J Nucl Med Mol Imaging (2017) 44(Suppl 1): S97-110.

Colleran GC, Kwatra N, Oberg L, Grant FD, Drubach L, Callahan MJ, MacDougall RD, Fahey FH, Voss SD (2017) How we read pediatric PET/CT: indications and strategies for image acquisition, interpretation and reporting. Cancer Imaging (2017) 17 (1):28. doi:10.1186/s40644-017-0130-8

Hasenclever D, Kurch L, Mauz-Körholz C, Elsner A, Georgi T, Wallace H et al. qPET – a quantitative extension of the Deauville scale to assess response in interim FDG PET scans in lymphoma. Eur J Nucl Med Mol Imaging (2014). 41: 1301 – 1308

Sandlund, J. T., Guillerman, R. P., Perkins, S. L., Pinkerton, C. R., Rosolen, A., Patte, C., Reiter, A. and Cairo, M. S. (2015). International Pediatric Non-Hodgkin Lymphoma Response Criteria. J Clin Oncol. 33(18), 2106–2111.

Voss, SD. Surveillance imaging in pediatric Hodgkin Lymphoma. Curr Hematol Malig Rep. 2013 8(3):218-25

#### A36 Bone marrow imaging in children

##### Gabriele Masselli (gabriele.masselli@uniroma1.it)

###### Radiology Department. Umberto I Hospital. Sapienza University. Rome, Italy

Bone marrow is one of the largest organs in the body and is visible in every magnetic resonance (MR) imaging study. It is composed of a combination of hematopoietic red marrow and fatty yellow marrow, and its composition changes throughout life in response to normal maturation (red to yellow conversion) and stress (yellow to red reconversion). MR imaging is highly sensitive for detection of altered marrow signal intensity, and the T1-weighted spin-echo sequence provides the most robust contrast between yellow marrow and disease. Heterogeneous red marrow and red marrow hyperplasia can mimic marrow disease, but should be distinguished from neoplastic replacement (leukemia, lymphoma, primary bone sarcomas, hematogenous metastases) and expected posttreatment changes (radiation therapy, chemotherapy, colony-stimulating factor, bone marrow transplant). Nonneoplastic edema-like processes can also alter marrow signal intensity, including trauma, infection, inflammation (chronic recurrent multifocal osteomyelitis, juvenile inflammatory arthritis), altered biomechanics, and chronic regional pain syndrome. Unfortunately, MR imaging findings are often nonspecific and overlap among many of these vastly different causes.

PET-CT has a high sensitivity when assessing marrow infiltration in pediatric malignancies. Advances in radiologic modalities may obviate the use of invasive, painful, and costly procedures like bone marrow biopsy. Furthermore, biopsy results are limited by insufficient tissue or the degree of marrow infiltration (diffuse vs. focal disease). PET-CT can improve the precision of biopsy when used as a guiding tool.

Therefore, a definitive diagnosis is reliant on a combination of imaging findings, clinical evaluation, laboratory assessment, and occasionally tissue analysis.

**References**

Chan BY et al. MR Imaging of Pediatric Bone Marrow. Radiographics. 2016 Oct;36(6):1911-193

Burdiles, Babyn PS. Pediatric bone marrow MR imaging. Magn Reson Imaging Clin N Am. 2009 Aug;17(3):391-409

Hassan A et al. 18F-FDG PET-CT imaging versus bone marrow biopsy in pediatric Hodgkin's lymphoma: a quantitative assessment of marrow uptake and novel insights into clinical implications of marrow involvement. Eur J Nucl Med Mol Imaging. 2017 Jul;44(7):1198-1206

Raissaki M et al Multifocal bone and bone marrow lesions in children - MRI findings. Pediatr Radiol. 2017 Mar;47(3):342-360. doi: 10.1007/s00247-016-3737-1.

Cistaro A, et al. Italian Multicenter Study on Accuracy of 18F-FDG PET/CT in Assessing Bone Marrow Involvement in Pediatric Hodgkin Lymphoma. Clin Lymphoma Myeloma Leuk. 2018 Apr 14. pii: S2152-2650(18)30011-9

### Tuesday 9^th^ October – Morning Session: 8:30 – 10:30 Multiparametric Imaging

#### A37 Multiparametric MR imaging of tumour biology

##### A R Padhani (anwar.padhani@stricklandscanner.org.uk)

###### Consultant Radiologist and Professor of Cancer Imaging, Paul Strickland Scanner Centre, Mount Vernon Cancer Centre, Rickmansworth Road, Northwood, Middlesex, United Kingdom

**Introduction**

Cancer development is characterised by the development of several biological capabilities (cancer hallmarks), which include sustained proliferative signalling, evading growth suppressors, resisting cell death, replicative immortality, inducing angiogenesis, and the activation of invasion mechanisms and metastasis. Mutations in multiple hallmarks are needed for tumor development, progression, spread and therapy resistance. Imaging techniques can map these hallmarks, showing tumour heterogeneity in their distribution, and in so doing have impacts on tumour diagnosis and management.

Precision oncology requires detailed knowledge of changing tumor biology, which biopsies (either targeted/liquid) cannot provide, especially for serial & heterogeneous assessments in therapy settings. The ability of functional & molecular imaging to enable noninvasive, quantitative measurements of tumor biology in-situ, allows of the full disease burden to be assayed serially. In so doing, imaging of therapy targets, drug pharmacokinetics and pharmacodynamics can provide markers of therapy efficacy, which are complementary to tissue-based biomarkers.

***Tumour proliferation and clonal mutations***

Cancers growth arises from cumulative, non-lethal genetic damage in mechanisms that normally control cell proliferation. Mutations and epigenetic DNA methylation enhance cellular circuits able to maintain proliferation, despite ‘bottlenecks’ of lethal mutations, immunity and environmental (including treatment related) barriers. At the same time, mutations also drive other cellular machinery needed for sustaining growth, progression and therapy resistance.

It is possible to observe tumour cellularity and cell viability with diffusion MRI, and to quantify diffusion imaging using ADC values (x10^-3^ mm^2^/s; μm^2^/s). The mechanisms of programmed tumor cell death (PCD) affects ADC changes in response assessment settings. For example, necroptosis results in the death of large numbers of cancer cells, including stroma destruction. There is much inflammation and extracellular oedema accompanying cell and nuclear lysis (membrane disruption) which results in marked increases in free water pool and higher ADC values. On the other hand, apoptotic cell death results in fewer cells being lost without adjacent cell/stroma damage. Organelles and proteins in apoptotic bodies are intact. Lipid droplets are present and little inflammation is observed. Therefore, ADC change depends on balance between apoptosis, autophagy & tumour repopulation, and only modest ADC rises maybe seen.

***Angiogenesis***

Neoangiogenesis is critical for tumour growth and driven by low tissue oxygen levels. Developing neovasculature is disorganised, with irregular and tortuous vessels. Structural abnormalities of tumour vessels are the basis for differential contrast enhancement. The clinical deployment of anti-vascular drugs has made imaging of angiogenesis important in assessing therapeutic efficacy. Traditional anatomic imaging is insensitive to detect early changes as antiangiogenic therapies are cytostatic. Multiple quantitative imaging techniques can depict the functional properties of neoangiogenic blood vessels. Two major dynamic contrast enhancement MR techniques are commonly used: dynamic susceptibility (T2*W DSC-MRI) and dynamic contrast enhancement (T1W DCE-MRI).

***Metabolic reprogramming in cancer***

The oncogenes promoting cancer cell proliferation are involved in the metabolic transformation via HIF-1^α^/MYC, PI3/AKT, mTOR & AMPK pathways. Mitogenic signals promote nutrient uptake & synthesis of building blocks of DNA, RNA, proteins and lipids. Heterogeneity in environment pO_2_, pH levels and nutrient availability combine with altered tumor metabolism, resulting in a continuous supply of building blocks that allow cancer cells to survive and proliferate under selective pressures.

Common metabolic events in cancer include (1) anaerobic glycolysis even in conditions of high oxygen tension (Warburg phenomenon), (2) increased pentose phosphate pathway for nucleotide synthesis (RNA/DNA), (3) increased serine biosynthesis: one-carbon metabolism for amino acid & folate metabolism, (4) shutdown of aerobic metabolism (with increased aerobic glycolysis) results in increased glutamine metabolism to replenish the TCA cycle, for fatty acid biosynthesis & amino acid metabolism. Multiple other tissue specific metabolic event also occurs, for example in prostate cancer where zinc and citrate metabolism are enhanced.

Work in for studying metabolism using hyperpolarized 13C compounds and dynamic nuclear polarisation is mostly undertaken in preclinical work to date and human work in its infancy. However, MR spectroscopy allows limited evaluations of human tumor metabolism. MRS observations in cancer include (1) the appearance or relative elevations of known metabolites (lactate, lipids, choline), (2) reductions of metabolites that should be present because of neoplastic replacement including N-acetylaspartate (neuronal marker) and citrate & polyamines (prostate glandular markers).

The choline signal conveys information on cell membrane synthesis (Kennedy pathway) and degradation. Free lipids depict necrosis & apoptosis in tumours, whereas the lactate signal informs on the level of anaerobic metabolism.

***Challenges multiparametric imaging in cancer***

1. Integrating multiple, spatially heterogeneous 4D imaging biomarkers at patient/region/lesion levels; this is a bioinformatics challenge suitable for artificial intelligence approaches.
2. Understanding the biology behind the image (gene expression profiles, proteomics, serum & urinary biomarkers) and the likely therapeutic implications.
3. Dealing with heterogeneity that exists within lesions, between lesions (in the same patient) between patients, and over time (in response to therapy.)
4. Developing roadmaps for multiparametric imaging BM qualification, fit for drug development and for aiding precision oncology developments.

**Conclusions**

Multiparametric imaging enables biologic assessments of cancer because of its multidimensional and quantitative nature. Imaging biomarkers are multispectral (multiple imaging biomarkers reflecting multiple biologic properties), multispatial and temporally resolved. Multiparametric imaging enables more accurate detection, localization, characterization and response assessments. Precision oncology (*right treatment for the right patient, at the right time, for the right duration*) requires appropriately validated imaging biomarkers (and tissue BMs). Imaging biomarkers development frameworks are accelerating the adoption of quantitative imaging in drug development and for high precision oncology

**Key references**

Hanahan D, Weinberg RA. Hallmarks of Cancer: The Next Generation. Cell 2011; 44 (5): 646–674.

Wan L, Pantel K, Kang Y. Tumor metastasis: moving new biological insights into the clinic. Nat Med. 2013; 19(11):1450-64.

Tan CP, Lu YY, Ji LN, Mao ZW. Metallomics insights into the programmed cell death induced by metal-based anticancer compounds. Metallomics. 2014; 6(5):978-95.

Cell Death - Autophagy, Apoptosis and Necrosis. Edited by Tobias M. Ntuli, ISBN 978-953-51-2236-4, 444 pages, Publisher: InTech,

Perfusion MRI in the early clinical development of antivascular drugs: Decorations or decision-making tools? M Zweifel, AR. Padhani. European Journal of Nuclear Medicine and Molecular Imaging – 2010; 39:164-182

Cairns RA, Harris IS, Mak TW. Regulation of cancer cell metabolism. Nat Rev Cancer. 2011; 11(2):85-95.

Martinez-Outschoorn UE, et al. Cancer metabolism: a therapeutic perspective. Nat Rev Clin Oncol. 2017; 14(1):11-31.

Lee G, Lee HY, Park H, Schiebler ML, van Beek EJ, Ohno Y, Seo JB, Leung A. Radiomics and its emerging role in lung cancer research, imaging biomarkers and clinical management: State of the art. Eur J Radiol. 2017; 86:297-307

Imaging biomarkers roadmaps for cancer imaging. O’Connor JPB et al. Nat Rev Clin Oncol. 2017; 14(3):169-186

#### A38 Multiparametric imaging of prostate

##### Jelle Barentsz and Roel Mus

###### Radboud University Medical Centre, Department of Radiology and Nuclear Medicine, Nijmegen, the Netherlands

Please see www.MRI-prostate-Barentsz.nl for papers referred to in the talk.

#### A39 Multiparametric imaging for early response assessment in head & neck cancer

##### A.D. King (King2015@cuhk.edu.hk)

###### Chinese University of Hong Kong, Hong Kong, PRC

Head and neck cancer treatment

Head and neck squamous cell carcinoma is the most common cancer in the head and neck. Concurrent chemoradiotherapy is the standard non-surgical treatment for advanced stage disease but ~ 25-50% of cancers are resistant to treatment. Therefore, there is a need for response assessment at primary and nodal sites early in the course of treatment. Treatment options for patients with non-responding cancers include radiotherapy escalation, addition of targeted agents, early cessation of additional induction chemotherapy regimens, or change to surgical treatment, while potential options for patients with responding cancers include de-escalation of treatment to reduce side effects. In addition, response assessment early after the end of treatment (~6 weeks) is desirable to allow more timely detection of patients with residual cancer in the head and neck who would benefit from salvage surgery or a radiotherapy boost.

Imaging modalities and assessment parameters

Assessment of treatment response has traditionally been based on change in tumour size, but size reduction during treatment lags response and may not be an accurate indicator of long-term outcome. Furthermore, in the post-treatment setting residual masses may be caused by residual cancer or benign post-treatment inflammation and scarring. MRI and FDG-PET provide valuable additional functional parameters for response assessment. These two modalities are being used in the post-treatment setting and are under intense investigation for intra-treatment assessment. This lecture will emphasise the role of MRI which has the advantage of providing both anatomical and functional parameters in the same examination. The emphasis will be on functional MRI using DWI but will also include DCE-MRI, MRS and CEST, and the complimentary role of functional MRI and FDG-PET in multiparametric imaging. Standardisation of functional MRI techniques remains problematic (imaging protocols, map analysis methods and outcome measures) and is complicated by an increasing array of MRI parameters and differing thresholds. Despite these limitations there are promising trends in early response assessment and these are summarised below.

Early intra-treatment response assessment

DWI is the most promising functional MRI technique. In the first few days of treatment cancers may show a transient decrease in ADC (cell swelling), but thereafter responding cancers show an increase in ADC as cells die, this increase being aided in the early phase by a radiation-induced increase in perfusion. Differences between responders and non-responders are detected early after the start of treatment (~ 2 -3 weeks), responders tending to have a significantly higher rise in ADC (> 14% - 24%) and shift of ADCs to the high ADC range, compared to non-responders. Further research is still ongoing regarding the exact timing of the first intra-treatment scan and the value of serial imaging, but a fall in ADC after an initial rise (repopulation of tumour cells) is linked to an unfavourable response. There is less research into DCE-MRI and FDG-PET, but early intra-treatment rises in K trans and Ve (2 weeks), and larger decreases in FDG activity (1-2 weeks) have been observed in responders compared to non-responders. Very early results using CEST imaging observe a rise in the levels of amide protons in non-responders, while MRS at 2 weeks has not been shown to predict response.

Early post-treatment response assessment

Post-treatment assessment is based on anatomical imaging, a focal residual mass of ≥1 cm and/or reduction in size (<50% primary site and < 80-90% nodal site) indicating high likelihood of residual cancer. However, anatomical criteria have low PPVs which are problematic, especially in the early post-treatment assessment of large necrotic HPV-related nodes which take time to resolve. The addition of T2-weighted imaging and DWI improves the diagnostic performance of MRI 6 weeks after the end of treatment. Residual cancers tend to show intermediate T2 signal intensity and restricted diffusion (b-1000 high + ADC map low signal intensity) in the solid component of a mass, like that seen before treatment. MRS also shows a persistent choline peak in residual cancers. Studies have shown that DWI is complimentary to FDG PET, reducing the FDG-PET false positive results for persistent cancer caused by post-treatment inflammation, so reducing the number of unnecessary examinations under anaesthesia.

Summary

Multiparametric imaging of head and neck cancer has a role in early post-treatment response assessment, and is close to being used for early intra-treatment response assessment. Advances in response assessment will accelerate as new functional imaging techniques emerge and more features can be extracted and analysed from existing large data sets using computer assisted interpretation (radiomics).

#### A40 Radiomics and AI: future of cancer imaging without radiologists?

##### H.-P. Schlemmer

###### German Cancer Research Center, Heidelberg, Germany

There is compelling evidence that recent advances in computer science will play a vital role in future medicine assisting diagnosis and treatment (Rahmesh AN 2004). Decision support systems are already in routine use in hospitals for selecting most appropriate imaging methods and imaging protocols according to the latest international guidelines. Computer science has important potential to facilitate and analyse US, CT and MRI images in oncology e.g. automated cancer detection, segmentation and characterization as well as quantitative therapy response assessment. Various artificial intelligence (AI) techniques including e.g. neuronal networks, fuzzy logic, evolutionary computation, deep learning or computer vision are currently emerging. Radiomics refers to sophisticated computer algorithms that extract numerous quantitative imaging features (mostly from ROIs pre-selected by radiologists). Various applications are currently emerging including e.g. automated detection and characterization of lung, breast and prostate cancer.

Novel healthcare informatics and communication technology open amazing opportunities for improving cancer imaging. With the increasing demand for cancer imaging, ever greater demand for expert knowledge in oncologic radiology is developing. But oncologic imaging specialists are usually located only in larger hospitals and dedicated care cancer centers, which, in turn, are located only in larger metropolitan areas. As a consequence, staffing shortage in rural, low-resource and less-educated areas will be inevitable. For a hospital administration it may even be easier to purchase high-performance imaging devices than to recruit imaging specialists. Accordingly, actual imaging opportunities may lie dormant. Support of imaging decisions for best possible utilization of local radiologic capabilities is feasible using the novel communication technology (DiPiro PJ 2017). Oncologic radiologists are in particular familiar with the situation that imaging studies acquired at other institutions are transmitted to their centers for second-opinion reporting and follow-up assessment. An extended radiology consulting service can accordingly include recommendation and implementation of state-of-the-art imaging protocols, quality control, image interpretation and documentation of the imaging studies according to most recent guidelines. Even clinical decision support by participation in interdisciplinary tumor boards sessions is on principle possible from a technological point of view. The growth of the internet with broad band capabilities has made teleradiology already a daily experience for many radiologists. National telemedicine programmes are currently under development in order to share expertise for improved cross-border medical services (Saliba V 2012). For the time being, however, major obstacles still hinder this progress including (1) legal aspects including data privacy, (2) financing, (3) local infrastructure and resources and (4) cultural factors (Saliba V 2012).

Cross-border telemedicine combined with AI may have significant impact on improved care of cancer patients, particularly in rural areas and developing countries where expert knowledge is not available. Exploiting the opportunities of AI, consulting oncologic radiologists may act as remote “information specialists” providing access to a specialist service (Jha S 2016). Current research on AI is emerging considerably, and further advancements in healthcare informatics and the built-up of supra-regional networks will help to overcome barriers. Novel IT technologies open the window to improving cancer imaging and making a high-quality imaging service available for more patients.

References:

DiPiro PJ, Krajewski KM, Giardino AA, Braschi-Amirfarzan M, Ramaiya NH. Radiology Consultation in the Era of Precision Oncology: A Review of Consultation Models and Services in the Tertiary Setting. Korean J Radiol. 2017; 18(1): 18-27

Jha S and Topol E. Adopting to Artificial Intelligence. Radiologists and Pathologists as Informaation Specialists. Jama 2016; 316(22): 2353-4

Rahmesh AN, Kambhampati C, Monson JR, Drew PJ. Artificial intelligence in medicine. Ann R Coll Surg Engl 2004; 86: 334–338

Saliba V, Legido-Quigley H, Hallik R, Aaviksoo A, Car J, McKee M. Telemedicine across borders: a systematic review of factors that hinder or support implementation. Int J Med Inform. 2012; 81(12): 793-809.

### 11:00 – 11:30 Keynote Lecture 3

#### K3 Cancer imaging: through a looking glass

##### Dow-Mu Koh

###### Royal Marsden Hospital, Sutton, Surrey, UK

Imaging in cancer has become an indispensable tool for treatment decision making, its success has been driven to a large extent by technical innovation in the past decades, which allows us to pin-point small volume disease, characterise cancers and their biology, assess the effectiveness of therapies and provide novel prognostic information. However, the pace of change poses a challenge to our discipline, as new technologies are often released before recent introductions can be thoroughly tested or validated. Nonetheless, promising developments have often filtered quickly into clinical practice.

Cancer imaging is at the cusp of another major revolution, which will be driven by artificial intelligence/ machine learning and big data. Radiologists should prepare to embrace these developments, by active engagement with stakeholders to define the role of machines in radiological workflows, so that machines can work synergistically with and support humans to be better and more effective radiologists. There is an immense opportunity for radiologists to become imaging physicians, who are more active in directing patient care and management alongside our clinical colleagues.

The coming era of growing reliance on machines and potential information overload from multi-modal and integrated diagnostics can lead to a highly de-personalised radiological profession when paradoxically providing increasingly personalised medical data. In such an environment, we run the risk of becoming mere providers of information, rather than communicators of knowledge or wisdom. We should use artificial intelligence to free up time to enable synthesis of new knowledge from the information we gather, which is subsequently distilled into wisdom for clinical practice. Radiologists have become increasingly isolated from direct patient contact, and there is chance to re-orient this relationship, which will help foster greater understanding of our profession and better patient-centred engagement. To maintain the relevance of our profession, it is important for radiologists to re-think their role in the new healthcare enterprise. Our decisions today will shape the practice of tomorrow.

### 11:30 – 13:00 Liver Tumours II

#### A41 Post-therapeutic changes

##### Wolfgang Schima

###### Department of Diagnostic and Interventional Radiology, Göttlicher Heiland Krankenhaus, Barmherzige Schwestern KH, and Sankt Josef-KH, Vinzenzgruppe, Vienna, Austria

In the past few years, great improvements have been made to achieve tumour control of primary and secondary liver malignancies. To date, hepatic resection is considered the most effective treatment option for metastases, CCC, and early stages of HCC. However, tumour ablation has gained considerable ground as an alternative to surgery. And transarterial chemoembolization (TACE) is the standard of care as treatment for HCC in intermediate stage, but it may also have potential for treatment of liver metastases. Highly efficient combinations of chemotherapeutic and antiangiogenic may lead to complete disappearance of metastases (histologic complete remission) or at least they will prolong life in palliative situations. Thus, the role of imaging not only to plan treatment, but also for assessment of therapy response and follow-up is of utmost importance [1].

Contrast-enhanced multiphasic MDCT is widely used in the evaluation of HCC after local tumour treatment [2]. Necrotic lesions appear in general as low-density areas with a thin rim of contrast enhancement in the arterial and portal venous phase, which may represent granulation tissue. Another frequent finding is the presence of perilesional parenchymal enhancement, which likely represents a benign physiologic hemodynamic response [3]. In contrast, residual tumour after incomplete tumour ablation or TACE may display focal or nodular peripheral enhancement in the arterial or portal venous phase. At MRI, DWI has been found be helpful to detect early tumour recurrence, together with dynamic gadolinium-enhanced imaging (with subtraction). In the hepato-biliary phase of liver-specific MR contrast agents, tumour necrosis as well as viable tumour may display low signal intensity (due to lack of hepatocellular uptake).

A variety of extrahepatic primary malignancies spread to the liver via the arterial or portal venous blood supply to seed metastases. Colo-rectal cancer patients with a limited number of liver-only metastases clearly benefit from surgical resection. Tumour ablation (either by radiofrequency, microwave, electroporation or laser) has shown great promise as an alternative for patients, who refuse surgery or in whom surgery resection is contra-indicated for medical reasons. As a joint procedure in combination with surgery it helps to achieve parenchyma-sparing local tumour control in patients with deep lesions. Side-by-side comparison of the pre- and post treatment CT scan (with the knowledge of the procedure performed) may help in the confident diagnosis of residual tumour post treatment: it shows if the size of necrosis in the post treatment scan actually covers completely the tumour seen on the pre-contrast scan. At MRI, necrosis post tumour ablation may show low signal intensity on T2-weighted images and high signal intensity on T1-weighted images due to coagulation necrosis. Dynamic T1-weighted GRE pulse sequences are recommended to show perfusion of vital tumour, which helps to assess therapy response. However, a recent study demonstrated that a single T1w GRE pulse sequence with fat-suppression is very accurate in predicting treatment efficacy after tumour ablation [4]. Volumetric portal venous phase enhancement as a sign of response has been shown to be associated with survival [5]. Gadolinium enhancement is also key to detect tumour recurrence. DWI has surpassed T2w fat-suppressed TSE imaging for detection of local tumour recurrence and new intrahepatic metastases.

Recently dynamic contrast-enhanced perfusion MRI assessment of tumour response to anti-angiogenesis drugs was described. These results suggest that dynamic contrast-enhanced MRI may serve as a precise biomarker to monitor pharmacological response to angiogenesis inhibitors than measurements of tumour diameter.

In summary, contrast-enhanced multi-phasic MDCT and MRI are the best tools to assess tumour response to therapy. Multiparametric MRI with diffusion-weighted imaging and dynamic gadolinium-enhanced sequences (and perfusion assessment) is very sensitive to assess local therapy response. In patients with treated liver metastases, MDCT has the advantage to monitor liver and extrahepatic disease.

**References:**

**1.** Bajpai S, Kambadakone A, Guimaraes AR, Arellano RS, Gervais DA, Sahani D. **Image-guided treatment in the hepatobiliary system: Role of imaging in treatment planning and posttreatment evaluation.** *Radiographics.* 2015;35:1393-418.

2. Lencioni R, Crocetti L. **Local-regional treatment of hepatocellular carcinoma.** *Radiology* 2012;262:43-58

3. Spina JC, Ulla M, Yeyati EL, et al. **MDCT findings after hepatic chemoembolization with DC-beads: what the radiologist needs to know.** *Abdom Imaging.* 2013;38:778-84.

4. DOnofrio M, Cardobi N, Ruzzenente A, et al. **Unenhanced magnetic resonance imaging immediately after radiofrequency ablation of liver malignancy: preliminary results.** *Abdom Radiol.* 2018;43:1379-1385.

5. Corona-Villalobos CP, Halappa VG, Geschwind JFH, et al. **Volumetric assessment of tunmour response using functional MR imaging in patients with hepatocellular carcinoma treated with a combination of doxorubicin-eluting beads and sorafenib.** *Eur Radiol* 2015;25:380-390.

#### A42 LI-RADS: necessary or not?

##### JP Heiken

###### Mayo Clinic, Rochester, Minnesota, USA

Hepatocellular carcinoma (HCC) is the second most common cause of cancer-related mortality worldwide. When recognized at early stages, HCC can be cured with appropriate therapy, which may involve resection, percutaneous ablation, transarterial chemoembolization or orthotopic liver transplantation (OLT). Since 2001, multiple expert panels in different parts of the world have advocated the noninvasive diagnosis of HCC if certain imaging criteria are met. Therefore, accuracy of classifying lesions as HCC or non-HCC on imaging studies is critical to patient management.

All of the consensus guidelines for the diagnosis of HCC prior to LI-RADS have important shortcomings, which supports the need for LI-RADS. None of the guidelines prior to LI-RADS defines or illustrates the pertinent imaging features on which the noninvasive diagnosis of HCC is based. LI-RADS not only establishes standardized imaging feature terminology, it precisely defines the imaging features and provides illustrations for clarification.

Secondly, none of the guidelines prior to LI-RADS has tried to clarify the probability that a lesion not fulfilling the criteria for definite HCC might be HCC. LI-RADS addresses this shortcoming by providing a comprehensive system to categorize each liver observation based on the likelihood that it is HCC, a non-HCC malignancy or a benign abnormality. A number of recent studies have provided data validating the appropriateness of the LI-RADS categories.

LI-RADS is of particular importance in the United States and Western Europe where OLT is considered the treatment of choice for patients with cirrhosis and HCC. Currently, approximately 25% of liver transplants performed in the United States are on patients with HCC, because patients with cirrhosis who develop HCC are given additional priority on the liver transplant waiting list independent of their liver function. Thus, the conservative approach of LI-RADS to the noninvasive diagnosis of HCC, which favors specificity over sensitivity, makes sense in the United States. The rationale is that if a patient with an imaging diagnosis of HCC can potentially receive higher priority on the transplant waiting list than a patient with poorer liver function but no HCC, then we need to be as certain as possible that the diagnosis is accurate. Such considerations are similar in Western Europe where 12% of liver transplants are performed on patients with HCC.

An additional advantage of LI-RADS over other consensus guidelines for the diagnosis of HCC is that by standardizing terminology, interpretation, reporting and imaging management LI-RADS improves communication among radiologists, hepatologists and surgeons, thus enhancing the opportunity to improve patient outcomes.

### Poster Abstracts

#### P1 Typical and atypical presentations of paediatric solid tumours

##### Pace E, MacVicar D. (Erika.Pace@rmh.nhs.uk)

###### The Royal Marsden, London, UK

**Learning objectives:**

- To highlight radiological features of common paediatric extracranial neoplasms correlated with demographic, clinical and pathological information;
- To illustrate demographically atypical presentations.

**Content organisation:**

Wilms’ tumour is the commonest renal tumour in children, characterised by large mass lesions, often with the characteristic ‘claw sign’, within the renal parenchyma. Peak incidence is 3-4 years, but the tumour occasionally occurs in adults. The demographics of renal tumours are discussed.

Rhabdomyosarcoma is pathologically diverse with a variety of cell types, and can occur at any site, although head & neck and pelvic tumours account for the majority of cases. It is anatomically heterogeneous and may occur in young adults.

In paediatrics, hepatoblastoma is the most frequent liver tumour. However, as age increases, a wider differential of liver tumours emerges which will be illustrated.

Typically “adult tumours” may occur in children, for example nasopharyngeal carcinoma and other head & neck tumours, adrenal carcinoma and a variety of sarcomas. Examples will be presented.

**Conclusions:**

Correlation with demographic information is a fundamental of paediatric tumour diagnosis. However it should be recognised that typically adult tumours may occasionally present in children, and vice versa.

#### P2 Prevalent diagnostic discrepancy patterns found in chest x-ray error cases

##### SJJ Touyz, M MacKenzine, K Kim, P Janousek, G Grazvydas

###### Pennine Acute Hospitals NHS Trust, Manchester, UK

####### **Correspondence:** SJJ Touyz (sarah.touyz@doctors.org.uk)

**Background:**

Diagnostic radiological errors are a recognised issue, with ~30% error rates in chest x-rays(CXR) often leading to medicolegal problems. Regular discrepancy meetings analyse errors and are encouraged for quality improvement by local trusts and the Royal College of Radiologists. Reviewing error cases improves diagnostic outcomes through education.

**Aims:**

This study retrospectively reviews CXR error cases referred to local discrepancy meetings at a general district hospital in an acute multi-site trust in the UK. The aims are to: (i) iidentify recurrent patterns of errors for quality improvement, (ii) highlight patterns/areas needing further analysis during reporting, (iii) and scrutinise current practice of discrepancy meetings.

**Methods:**

Retrospective analysis of 51 CXR error cases from discrepancy meetings was done from 2009-2017 using PACS/CRIS. A database with various parameters was made for statistical analysis.

**Results:**

The average patient age was 68.5, with 56% males and 44% females. 76% had a history of smoking.>60% of discrepancy errors were perceptual, 29% cognitive and 8% communicative. Most errors were primary malignancies(>55%) and infections(14%). Missed lesions were in the peripheral(41%), hilar(30%) and parahilar(24%) regions. 40% of cases were in the upper zones. The most common referred symptom was cough, and most referrals came from A&E.

**Conclusion:**

Discrepancy meetings educate reporters and hope to improve diagnostic accuracy (particularly for malignancies). Perceptual errors were the most common error made in CXR reporting. Peripheral and upper zones in smoking patients referred with cough need extra scrutiny when reporting. Improvements to current discrepancy meeting methodology is encouraged to optimise outcomes and effectiveness of the meetings and ensure quality improvement for future patient outcomes.

#### P3 Imaging features of osteochondromas and chondrosarcomas

##### Khin Y Thein^1^, Hsueh W Cheong^2^, ^3^Wilfred C.G. Peh^3^

###### ^1^Khoo Teck Puat Hospital, Singapore, Singapore; ^2^Changi General Hospital, Singapore, Singapore; ^3^Khoo Teck Puat Hospital, Singapore, Singapore

####### **Correspondence:** Khin Y Thein (kythein@yahoo.com)

**Learning objectives:**

To review the spectrum of imaging findings of osteochondromas and chondrosarcomas in different imaging modalities.

**Content organisation:**

Osteochondromas are one of the commonest bone tumours, and are most often found at the ends of long bones. Often solitary, the development of multiple osteochondromas is characteristic of diaphyseal aclasis (or hereditary multiple exostosis) in which the risk of malignant transformation to chondrosarcoma is increased. Chondrosarcoma varies in histology and prognosis; mesenchymal and dedifferentiated tumours have the worst prognosis with high rates of local recurrence and metastasis. Multimodality imaging has a useful and complementary role in enabling lesion characterisation, detection of lesion extent and malignant change, surgical planning and staging.

We will discuss imaging characteristics of osteochondromas and chondrosarcomas and their complications as well as pseudotumour that mimics chondrosarcomas.

**Conclusions:**

Although plain radiograph is critical in the initial assessment of these tumours which are often characteristic, additional features on CT and MR images are invaluable adjunct tools for optimising tumour characterisation.

Understanding and recognising the spectrum of appearances of these tumours allow improved patient assessment and are vital for optimal clinical management, including diagnosis, biopsy, staging, treatment, and expectations for prognosis.

#### P4 NI-RADS: structured reporting of head and neck squamous cell carcinoma post-treatment imaging

##### Douglas J. Quint, Ellen Hoeffner, Remy Lobo, John Kim, Guarang Shah, Diana Gomez-Hassan, Ashok Srinivasan

###### University of Michigan Medical Center, Ann Arbor, Michigan, USA

####### **Correspondence:** Douglas J. Quint (djquint@umich.edu)

**Learning Objectives:**

a) To review a recently developed template [NI-RADS: Head and Neck Imaging Reporting and Data System] for reporting cross-sectional scans of patients’ status post-surgery, chemotherapy (chemotx) and/or radiation therapy (RT) for treatment of head & neck squamous cell carcinoma (SqCCa).

b) To understand how using this template adds value for clinicians and patients with respect to patient management and prognosis.

**Content Organisation:**

Similar to the 25 year old BI-RADS structured reporting template used for mammography, a template has been developed by an ACR group over the past 3 years for structured reporting of follow-up imaging performed on head/neck SqCCa patients who have undergone surgery, chemotx and/or RT.

Scan findings are divided into 4 categories based on imaging features which are reviewed and demonstrated in this exhibit.

The findings present on a post-therapy imaging study (CT, MRI, CT-PET) are reported with respect to the primary site of disease and the appearance of neck nodes. Based on imaging findings, scans can be classified with respect to suspicion of disease recurrence, associated risk of recurrent neoplasm and recommended management as:

**Table:** 

**Category**	**Description**	**Risk of Disease Recurrence**	**Recommended management**
1	No suspicion	4%	Surveillance
2	Low suspicion	17%	Direct inspection &/or short-term imaging follow-up
3	High suspicion	59%	Biopsy
4	Known recurrence	~100%	Treatment

**Conclusion:**

Adoption of a new structured template [NI-RADS] will add value to imaging performed in head/neck post-therapy SqCCa patients as well-defined categorization of findings should have reproducible implications with respect to patient management and prognosis.

#### P5 Pancreatic neuroendocrine tumours: a case-based imaging review with clinicopathologic correlation

##### Lindsey Storer, Jason Scapa, Steven Raman, Barbara Kadell, Maitraya Patel

###### University of California, Los Angeles, Los Angeles, California, USA

####### **Correspondence:** Lindsey Storer (lstorer@mednet.ucla.edu)

**Learning Objectives:**

The purpose of the exhibit is to:

1. Present a comprehensive multimodal discussion on the various subtypes of pancreatic neuroendocrine tumours (NETs), including insulinomas, gastrinomas, VIPomas, glucagonomas, somatostatinomas, and nonfunctioning NETs and their associated clinical features
2. Demonstrate the imaging features of neuroendocrine tumours on CT, MRI, and nuclear medicine imaging
3. Provide pathology correlation to imaging findings with review of tumour grading
4. Review treatment strategies, including surgery and minimally invasive techniques, such as ablation and chemoembolization

**Content Organisation:**

- Epidemiology of pancreatic neuroendocrine tumours
- Clinical features including associated syndromes (multiple endocrine neoplasia type 1, von Hippel-Lindau, neurofibromatosis type 1, and tuberous sclerosis), symptoms, and serum markers
- Multimodality imaging features and technical parameters for ideal detection – CT, MRI, endoscopic ultrasound, and nuclear medicine imaging (indium-111 octreotide scintigraphy, gallium-68 Dotatate PET)
- Pathology of tumour subtypes
- Staging implications and treatments

**Conclusion:**

Integrating knowledge of the multimodality imaging appearance with the clinical features of pancreatic neuroendocrine tumours is important in the diagnosis and management of these patients.

#### P6 Small bowel masses: an illustrative case-based review

##### Lindsey Storer^1^, Monica Deshmukh^2^, Barbara Kadell^1^, Matilda Jude^2^, Maitraya Patel^1^

###### ^1^University of California, Los Angeles, California, USA; ^2^Olive View - UCLA Medical Center, Los Angeles, California, USA

####### **Correspondence:** Lindsey Storer (lstorer@mednet.ucla.edu)

**Learning Objectives:**

The purpose of the exhibit is to:

1. Illustrate the wide range of malignant small bowel tumours including carcinoid, adenocarcinoma, lymphoma, gastrointestinal stromal tumours, leiomyosarcomas, and metastases, as well as benign lesions such as lipomas, leiomyomas, and polyps. Polyposis syndromes will also be reviewed.
2. Describe common and distinct imaging characteristics of small bowel masses
3. Integrate radiologic assessment with clinical features and treatment to provide guidance on further workup to referring specialists

**Content Organisation:**

- Epidemiology of malignant and benign small bowel masses
- Pathophysiology with histologic correlation
- Multimodality imaging appearance on CT, MRI, fluoroscopy, CT enterography, MR enterography, and nuclear imaging
- Clinical Features including presentation, treatment, and prognosis

**Conclusion:**

Small bowel tumours are rare, but their incidence has been increasing over the past several decades. Early diagnosis is often challenging for clinicians and knowledge of these tumours enables the radiologist to help guide clinicians in the workup of affected patients.

#### P7 Musculoskeletal pseudotumours

##### Santiago Andres, Delfina Pascuzzi, Ramos Paula, Pomés Felipe, Eyheremendy Eduardo

###### Hospital Alemán, Buenos Aires, Argentina

####### **Correspondence:** Delfina Pascuzzi (delfinapascuzzi@hotmail.com)

**Learning objectives:**

The objective of this presentation is to be able to distinguish conditions that on initial radiological studies might be confused with malignant lesions mimicking musculoskeletal tumours. Moreover, to review some patterns of benign lesions that can be mistaken for neoplasic lesions.

**Content Organisation:**

Musculoskeletal pseudotumours are not infrequent and is a term that has typically been used to indicate the presence of a mass which is felt to represent a neoplasm at some level of observation.

To improve MRI diagnosis for pseudotumours by radiologists it is important to correlate with radiographs, ultrasound, computed tomography and clinical history.

Musculoskeletal pseudotumours can be subdivided anatomically into those related to superficial and soft tissues and into those related to the bone.

Because of its unspecific clinical and radiological presentation, this disease constantly causes diagnostic and therapeutic difficulties.

We propose diagnostic tools for evaluation of musculoskeletal tumours and pseudotumours.

We included in the differential diagnosis lymphangioma, muscle hematoma, abscess, osteomyelitis.

**Conclusion:**

There are several diseases that can mimic musculoskeletal tumours.

It is necessary to take them into account and know how to use diagnostic keys for an accurate diagnosis.

#### P8 Non adenomatous lesions of the adrenal gland: CT and PET/CT findings

##### E. Casalini Vañek, P. Ramos, A. Monjes and EP Eyheremendy

###### Hospital Alemán. Buenos Aires, Argentina

####### **Correspondence:** P. Ramos (pramos@hospitalaleman.com)

**Learning Objectives:**

Describe the main differential diagnosis of non-adenomatous adrenal masses. Identify the main imaging findings of these entities.

**Content Organisation:**

Imaging studies of the adrenal glands are usually necessary in three clinical circumstances: patients who may have received a clinical diagnosis of adrenal hormone hyperfunction, patients who have undergone imaging studies of the suprarenal glands to confirm the suspicion of a metastatic disease, and finally, patients who underwent imaging studies for other indications and were accidentally detected a lesion on these glands.

These lesions - in symptomatic and asymptomatic patients - can be classified according to several criteria, namely: size, site of anatomical origin, morphological characteristics or biological behavior.

The differential diagnoses of non-adenomatous adrenal masses that we describe are:

- Simple cysts
- Pheocromocytoma
- Adrenal hiperplasia
- Adrenal hemorrhage
- Neoplasms

CT imaging is the preferred diagnostic imaging technique used for the detection and characterisation of adrenal lesions with reported specificity of identifying the mass as an adenoma being close to 100%, though the sensitivity is lower. Positron emission tomography is generally performed in patients with cancer and it yields information about the biochemical processes that may precede gross anatomic changes.

**Conclusion:**

The adrenal gland is involved in a variety of benign, malignant primary and metastatic tumours. The use of a correct study algorithm, with the appropriate and accessible diagnostic tools, allows the correct characterization of the injuries.

The role of the radiologist is of great importance in the identification and diagnosis of tumours and tumour-like conditions in symptomatic and asymptomatic patients.

#### P9 Utility of rectal MRI in the evaluation of low rectal cancer to help in the surgical planning

##### Nazar M, Ramos P, Pomes F, Castro Cavallo L, Escolar Navas M

###### Hospital Alemán, Buenos Aires, Argentina

####### **Correspondence:** De Luca S (sdeluca@hospitalaleman.com)

**Learning Objectives:**

To review the utility of high resolution MRI in identifying the correct surgical approach based on the relation of the tumour with mesorectal and extralevator planes.

**Content Organisation:**

Low rectal cancer ( < 5 cm from the anal verge) remains a global challenge due to high positive pathological circumferential resection margin rates principally attributed to anatomic considerations as the fact that the mesorectal fascia tapers downward at this level and higher perforation rates. Preoperative anatomically based High Resolution MRI systems may be able to predict which patients may be at risk from traditional surgery and enable patients to undergo a more radical excision, thus ensuring adequate resection margins.

We will review:

- MRI low rectal cancer plane staging.
- Key High Resolution MRI anatomical landmarks.
- Cases.

**Conclusion:**

Anatomically based High Resolution MRI staging for low rectal cancer is an excellent tool to reduced margin positive and to improve outcome in patients with low rectal cancer.

#### P10 Whole-body MRI: a pictorial review of incidental findings from a tertiary oncology centre

##### R Kulanthaivelu, B Shepherd, J Smart, A King, K Tung

###### University Hospital Southampton, Southampton, United Kingdom

####### **Correspondence:** R Kulanthaivelu (roshini@doctors.org.uk)

**Learning Objectives:**

To highlight the incidence and range of incidental findings that can be encountered with whole-body MRI by reviewing those encountered at our tertiary oncology centre in 2017.

**Content Organisation:**

Whole-body MRI is emerging as a useful tool in the management of oncology patients. Following the 2015 International Myeloma Working Group recommendations, whole-body MRI replaced skeletal surveys in myeloma assessment, because it is a highly sensitive examination with wide coverage that can both quantify disease extent and qualify treatment response without exposure to ionising radiation. New applications are subsequently being identified, e.g. assessment of metastatic prostate cancer and lymphoma staging. The high sensitivity and wide coverage associated with whole-body MRI however, comes at the cost of multiple incidental findings.

We present a pictorial review of incidental findings encountered at our tertiary oncology centre in 2017, illustrating the spectrum of disease that can be encountered.

- 57 whole-body MRI examinations were performed in 2017 as a part of routine myeloma assessment.
- 11 (19%) incidental findings were encountered ranging from thyroid eye disease, parotid lesions, multinodular goitre, hydronephrosis, pathological fractures, sacral haemangioma, lung, sigmoid and pancreatic cancers.
- 6 (11%) of these patients required additional imaging for further assessment.

**Conclusions:**

- Incidental findings are commonly encountered with whole-body MRI due to its high sensitivity (19% of studies).
- As radiologists, we need to be aware of the prevalence and spectrum of possible incidental findings that can be encountered while reporting these studies and know how to manage them so that potentially life-threatening diagnoses are not missed.

#### P11 Advanced non-small cell lung cancer CT characteristics in molecular medicine: correlation with ALK and EGFR molecular phenotype

##### Carrera Cecilia, De Luca Silvina, Troncoso Fiorella, Castro Cavallo Luciano, Monjes, Alejandra, Eyheremendy Eduardo

###### Hospital Alemán, Buenos Aires, Argentina

####### **Correspondence:** De Luca Silvina (sdeluca@hospitalaleman.com)

**Learning objectives:**

Review adenocarcinoma molecular features Investigate epidemiological characteristics of patients with molecular mutations to be able to subdivide them into study groups.

Establish the differences in computed tomographic (CT) characteristics between patients with advanced lung adenocarcinoma ALK gene rearrangement and those who have EGFR mutations.

**Contents Organisation:**

Adenocarcinomas are characterised by distinct genomic changes. ALK+ EGFR+ NSCLC have distinct epidemiological and CT characteristics regarding gender, age population and tumour size, margins, central or peripheral location, lymph node metastasis, intrathoracic metastasis and association with large pleural effusions.

Case reviews of 30 patients in our institution from January 2017 to April 2018. A triple blind analysis model was applied to discriminate clinical and CT characteristics between these types of mutation.

**Conclusions:**

ALK+ status was associated with male middle age patients, central tumour location, tumour lobulated margins, pleural and/or pericardic effusion and lymphangitic lung metastasis while those with EGFR + mutations where more common in older female patients and where associated with spiculated margins and a tendency for hematogenous tumour spread, such as miliary and bone metastasis.

#### P12 Liposarcoma histological subtypes with MDTC imaging correlation

##### Casalini Emilia, De Luca Silvina, Escolar Navas Marceliano, Rodríguez Gina , Eyheremendy Eduardo

###### Hospital Alemán, Buenos Aires, Argentina

####### **Correspondence:** De Luca Silvina (sdeluca@hospitalaleman.com)

**Learning objectives:**

Review the different histological subtypes of liposarcomas. Describe the findings encountered in imaging studies of liposarcoma variants. Know the histo / imagenologic correlation of liposarcomas.

**Content Organisation:**

The liposarcoma is a malignant tumour of mesodermic origin derived of the adipose tissue. Liposarcoma’s types, according to his histological diagnosis, are: mixoide, pleomorphic, well differentiated and dedifferentiated.

Five cases of liposarcomas were evaluated retrospectively in MDCT and MR performed between January 2016 and January 2018.

We found 3 cases with a well differentiated subtype, 1 case with a dedifferentiated subtype and 1 mixed subtype case (Well differentiated - myxoid).

**Conclusions:**

The images are indispensable in the evaluation of soft tissue masses. Although it often does not allow the diagnosis of certainty, they provide essential information for the surgeon.

Because of that, they are a useful tool for diagnosis as for the monitoring of these tumours.

The work of a multidisciplinary team, composed of radiologist, clinician, surgeon and pathologist is essential to obtain better results.

#### P13 TNM staging of pancreatic adenocarcinoma. MDCT-Histopathology correlation

##### De Luca Silvina, Carrera Cecilia, Di Cecco María de los Milagros, Barale Cintia, Eyheremendy Eduardo, Pablo Capitanich, Laura Mattioni

###### Hospital Aleman, Buenos Aires, Argentina

####### **Correspondence:** Barale Cintia (cintub@hotmail.com)

**Learning objectives:**

The main purpose of this exhibit is to correlate pancreatic adenocarcinoma CT imaging using TNM staging system to histopathological.

Perform the diagnosis and a correct staging of the pancreatic tumour, establishing criteria of resectability, and assisting the surgeon in the preoperative planning before the finding of relevant anatomical variants.

**Content organisation:**

Decisions about diagnostic management and resectability should involve multidisciplinary assessment with appropriate high-quality imaging studies to evaluate the extent of disease. Carefully timed scan acquisition allows accurate local and distant staging as well as assessment of local resectability. In addition, we evaluate vascular maximum intensity projection (MIP) images and curved MPR images obtained along the planes of the great vessels.

We review Multidetector volumetric computed tomography (MDCT) scans of 23 adult patients with known or suspected pancreatic adenocarcinoma performed between January 2009 and April 2018. Findings were then compared with surgical and pathologic results.

Our preliminary results showed difficulties to asses between similar tumour sizes, especially between T2 and T3 stages. However, we were successful in assessing T4 and M1 (86% average accuracy) and therefore determining unresectability of tumours.

**Conclusion:**

Based on our analysis, we conclude that it is difficult to perform a correct anatomo-radiological correlation, where the greatest discrepancy lies in objectifying the actual size of the tumour and its extension.

Nevertheless, promising results are obtained when evaluating resectability of the tumour since it has direct impact on the surgical/clinical management decisions.

TNM staging for pancreatic adenocarcinoma is not frequently included in radiology reporting but is a useful tool for the preoperative assessment of pancreatic adenocarcinoma.

#### P14 Somatostatin imaging: what we know to date and where we are headed

##### A Omar, T Gruning, B Drake

###### University Hospitals Plymouth NHS Trust, Plymouth, UK

####### **Correspondence:** A Omar (asha.omar@nhs.net)

The imaging of neuroendocrine tumours has evolved considerably over the past 40 years. Discovered only within the last 45 years, Somatostatin (Ss) is a neuropeptide which exerts its effects on multiple cell types, all over the body.

Approximately 10 years after the initial discovery of Ss, research into somatostatin receptor (SSTR) subtypes and the cellular pathways it affects really began to progress. There are five Ss receptor subtypes with different binding affinities for the neuropeptide; the most widely expressed receptors and therefore most useful for imaging are SSTR 2 and 5.

The creation of a Ss analogue and the ability to label it with an appropriate radionuclide, has been influential in discovering what we know about SSTR expression in both normal tissue and in pathological entities. This includes neuroendocrine tumours and has helped to evaluate how such tumours behave.

Traditional diagnostic imaging of neuroendocrine tumours using computed tomography (CT), has limitations in the sensitivity of tumour identification. Functional imaging using radiopharmaceuticals increases sensitivity significantly. Traditionally this has used gamma emitting radionuclides, namely Indium-111. Increasingly, positron emitting tomography (PET) imaging, using pharmaceuticals labelled with Gallium-68 is being performed due to the superior sensitivity in tumour detection. Furthermore, peptide receptor radionuclide therapy (PRRT) is a growing area of interest amongst both the nuclear medicine and oncology communities.

This educational poster seeks to provide the reader with a background into the history of Ss imaging and the range of pathologies that may be imaged. It will serve as a balanced overview of the imaging modalities available to date. We shall focus on functional imaging and discuss the advantages and limitations of using traditional gamma radionuclides as well as the increasingly available PET radiopharmaceuticals. We discuss the growing field of PRRT and consider what the future holds for both the imaging and treatment of neuroendocrine tumours.

#### P15 **RECIST and iRECIST criteria: our daily practical experience**

##### De Luca S, Carrera C, Di Cecco M, Barale C, Fernandez M, Monjes A, Eyheremendy E, Gómez Abuin G, Tamburelli M

###### Hospital Aleman, Buenos Aires, Argentina

####### **Correspondence:** Di Cecco M (mdicecco@hospitalaleman.com)

**Learning objectives:**

Describe our daily use of RECIST criteria in practice, including its limitations and define adequate comparison time points as an appropriate tool to interpret solid tumour response assessment.

Describe our initial experience in applying iRECIST in a clinical oncologic-protocol setting.

**Content organisation:**

Tumours respond differently to immunotherapies compared with chemotherapeutic drugs, raising questions about the assessment of changes in tumour burden—a mainstay in the evaluation of cancer therapeutics that provides key information about objective response and disease progression. A consensus guideline—iRECIST—was developed by the RECIST working group for use of modified Response Evaluation Criteria in Solid Tumours (RECIST version 1.1) in cancer immunotherapy trials.

New concepts were developed, including unconfirmed (iUPD) tumour growth and new lesions, confirmed progression (iCPD) and pseudoprogression.

iRECIST requires the confirmation of progression to confirm or discard pseudoprogression regarding temporal tumour changes caused by immunotherapy.

The main insight of RECIST 1.1 is to continue treatment and repeat imaging in case of a mixed response or equivocal findings.

The key point understanding of iRECIST criteria is to define real tumour status according to clinical and humoral settings that could require eventual change in management, so confirmation of progression needs at least a new scan in 4 weeks.

**Conclusions:**

RECIST and iRECIST criteria included in Radiology Reporting are a useful tool for oncologists to asses response to treatment. These must be chosen based on the Tumoural type and treatment. Multidisciplinary case study together with clear, thorough and interactive imaging reports are of key importance for success.

#### P16 The devastation of metastatic heart disease: spread, imaging, and treatment options

##### I. Masood, A. Saleem, T. Hunsaker, L. Frank

###### University of Texas Medical Branch, Galveston, Texas, USA

####### **Correspondence:** L. Frank (lufrank@utmb.edu)

**Learning objectives:**

To review the spectrum of metastatic disease to the heart and pericardium in correlation with types of spread, imaging characteristics, anatomic predilection, and treatment options.

**Content organisation:**

Metastases are the most common malignant cardiac tumours and by far more common than primary cardiac tumours. They can spread by direct invasion from adjacent malignancy, such as lung or mediastinal cancer, by lymphatic, or hematogeneous dissemination.

We will demonstrate each specific way of vascular, direct, and lymphatic metastatic spread to the heart with anatomic predilection of particular type of metastasis, using case by case approach.

We will review most common primary malignancies, known to metastasise to the heart and pericardium, including: uterine leiomyosarcoma, lung cancer, thymic cancers, carcinoid, melanoma, breast cancer, and lymphoma.

We will present diagnostic modalities and imaging characteristics of cardiac metastasis:

-Diagnostic modalities (Echocardiography, Computed Tomography, Cardiac MRI, PET-CT, and Coronary Angiography)

-Behavior and imaging characteristics of tumours, which are usually similar to the primary malignancy

-Patterns of Gadolinium enhancement

We will discuss surgical, radiation, and chemotherapy treatment options of cardiac and pericardial metastatic disease.

**Conclusions:**

Metastatic disease to the heart and pericardium remains under recognised despite accumulating evidence in literature and diagnostic capabilities of modern medicine. Cardiac metastasis is a devastating disease with poor prognosis, which almost always upstages the primary malignancy and significantly limits treatment options.

#### P17 Sleep abnormalities in non-CNS malignant tumours

##### Linda J Larson-Prior, Aaron S Kemp, Ahmad Baghal, Shorabiddin Syed, Ellyn E Matthews

###### University of Arkansas for Medical Sciences, Little Rock, AR, USA

####### **Correspondence:** Linda J Larson-Prior (ljlarsonprior@uams.edu)

**Aim**:

Sleep disorders are common cancer comorbidities that often remain a significant problem post therapy. In this study, we investigated commonly diagnosed sleep disorders and their relationship to cancer type and therapy.

**Methods**:

We mined electronic health record data from the UAMS Clinical Data Warehouse for patients diagnosed with lung, breast and prostate cancers. Of 13,192 cancer patients 1,261 had a diagnosed comorbid sleep disorder. To evaluate the impact of sleep disturbances, we compared prevalence of sleep disorders by cancer type, treatment modality, and comorbidities (anxiety, fatigue, and pain) by cross-tabulation using chi-square as a test statistic.

**Results**:

Of 1261 patients (20-93 years; 61.0±12.4, 71.5%F) 57.9% were diagnosed with breast (99.5%F), 27.2% with lung (51.0%F) and 14.9% with prostate cancers. The most common sleep comorbidity was sleep disordered breathing (SDB, 68.6%) followed by insomnia (41.6%). Breast cancer patients exhibited the highest levels of insomnia (51%), while lung cancer patients were most impacted by SDB (84%) with the lowest percentage of insomnia (26%). The most common sleep disturbance in prostate cancer was SDB (71%). Patients receiving hormone therapy for lung and prostate cancers, and women receiving chemotherapy for breast cancer, all showed significantly elevated prevalence of insomnia and lower prevalence of SDB, relative to patients who did not receive those respective therapies.

**Conclusions**:

We report a prevalence of 9.6% of patients with diagnosed sleep disorders, likely an underestimate of those with sub-clinical or undiagnosed sleep disturbances that may impact therapeutic response and quality of life.

#### P18 Prediction of breast lesion malignancy in high risk patients with BI-RADS 4 lesion on multiparametric MRI-Based radiomic

##### Camille Schreiner ^1^, Benjamin Leporq ^2^, Agnes Coulon ^1^, Olivier Beuf ^2^, Frank Pilleul ^1^

###### ^1^Département de Radiologie, Centre de lutte contre le Cancer, Lyon, France; ^2^Univ Lyon, INSA‐Lyon, Université Claude Bernard Lyon 1, UJM-Saint Etienne, CNRS, Inserm, CREATIS, Lyon, France

####### **Correspondence:** Frank Pilleul (frank.pilleul@lyon.unicancer.fr)

**Aim:**

To develop a multiparametric MRI (mpMRI)-based radiomic method to predict cancer in BI-RADS 4 breast lesions in high risk patients.

**Methods:**

46 high risk oncogenetic patients with BI-RADS 4 lesion, histology and 1.5T mpMRI data available were retrospectively enrolled. Fat-suppressed perfusion weighted (PWI) images at 3 min and subtraction; diffusion weighted (DWI), (b0 and 700) images and apparent diffusion coefficient maps were used as radiomic fingerprints.

Images were manually segmented by a radiologist to extract the radiome from each fingerprint. The radiome included 87 features describing shape, size, distribution and texture in images and frequency domain. Overall, 435 features were integrated. The learning base dimension was reduced using a backward selection by thresholding on t-test p-value. A *k*-nearest neighbor algorithm with a cosine node function and 10 neighbors was used as a classifier. Validation was performed with the holdout method (75% of data used for training and 25% for testing).

**Results:**

Based on t-test p-value, only texture and distribution features provided relevant descriptors to be included in model training. To classify between benign lesion and cancer, model performances were 0.95 AUROC with 100% (95% CI: 100 – 100%); sensitivity, 80% (95% CI: 45 – 115%) specificity, 85.7% (95% CI: 60 – 111%) positive predictive value, 100% (95% CI: 100 – 100%) negative predictive value and 90.9% (75% CI: 74 – 107%) accuracy (Fig.1).

**Conclusion:**

mpMRI-based radiomic may be helpful to non-invasively predict cancer among BI-RADS 4 lesion in high risk patients. These results need to be confirmed on a larger prospective validation cohort.

#### P19 CT guided percutaneous biopsies: a retrospective audit of 167 cases

##### Koween Vencatasawmy, David Walter, Bhavini Billimoria

###### Kettering General Hospital, Kettering, UK

####### **Correspondence:** Koween Vencatasawmy (Koween.vencatasawmy@nhs.net)

**Aims and Background:**

Computed Tomography (CT) Guided Biopsies are safe and effective but are a significant time burden on the CT scanner, competing with inpatient and outpatient scanning. We evaluated CT guided biopsies that were performed in our service over 5 years to assess diagnostic yield and safety.

**Materials and Methods:**

Retrospective data were analysed on all patients who underwent a CT guided biopsy from 1^st^Jan 2013 until 29 March 2018. Reports, study images and original requests were reviewed to determine biopsy site, biopsy material, patient demographics, clinical indication and complication rate. The histology reports and any immediate post biopsy scans were also reviewed to determine outcome.

**Results:**

Data from 172 CT guided Biopsies were performed by 9 Consultants and 5 Registrars over this 5-year period, 26.9% were referred by Haematologists, 16.2% by Medical Specialities and 56.9% by Surgical Specialities.

5 were not technically possible and abandoned (2.9%). Of the biopsies which were performed 151/167 (90.4%, 95% CI: 85.0% - 94.0%) of samples were diagnostic. 164 (98.2%) were performed for suspected malignancy, with 117 (71.3%) histological diagnoses of cancer identified. No complications of biopsies were found.

**Conclusion:**

Our findings show that our CT Guided Biopsy is a safe and effective service, with a low risk of complication and a high diagnostic yield.

#### P20 Diagnostic accuracy for malignancy in image guided intra-abdominal biopsies in a tertiary care radiology department

##### O O’Brien, O Cram (owen.o’brien@nhs.net)

###### Queen Elizabeth University Hospital, Glasgow, UK

**Aim:**

Tissue biopsies of intra-abdominal organs are indicated for diagnosing and staging primary malignancies, identifying metastases and diagnosing benign lesions. This audit evaluates the diagnostic yield and complication rates for intra-abdominal biopsies taken for clinically suspected cancer diagnoses in a tertiary care radiology department.

**Materials and methods:**

All image guided intra-abdominal biopsies taken for clinically suspected cancer diagnoses over 10 months were included. Histology reports, radiology reports, and clinical notes were analysed.

**Results:**

42 CT guided and 70 US guided biopsies were identified. 90% (101/112) of biopsies had a diagnostic yield, 83% (35/42) for CT guided and 94% (66/70) for US guided. 96% of biopsies with a diagnostic yield were positive for malignancy (97/101), 94% (33/35) of CT and 97% (64/66) of US guided biopsies.

Under CT guidance, kidneys were biopsied most frequently (12/42). The diagnostic yield was 92% (11/12). 91% (10/11) of these were diagnostic of malignancy, the majority were renal cell carcinoma (7/10). Under US guidance, liver was biopsied most frequently (50/70). The diagnostic yield was 96% (48/50). 98% (47/48) of these were diagnostic of malignancy, the majority were metastases (40/47). There was no significant difference between number of cores taken and whether the biopsy was diagnostic or non-diagnostic (p-value 0.97 and 0.08 for CT and US, respectively).

13% (15/112) experienced minor pain and bleeding. There were no other complications.

**Conclusions:**

A high diagnostic yield for malignancy was demonstrated in biopsies taken for clinically suspected cancer diagnoses. Complication rates were within acceptable limits.

#### P21 A more than one thousand four hundred patients experience

##### J Iorio, L Cuneo, F Bambacci, C Montenegro, M Brisco, L Fassola, J Coronil, D Polillo, FPeña, M Eleta

###### Imaxe, Buenos Aires, Argentina

####### **Correspondence:** J Iorio (drajiorio@gmail.com)

**Aim**:

The aim of this study is to present our experience between the correlation of 18 fluorocholine (FCH) positron emission tomography (PET)/computed tomography (CT) findings and PSA levels in detecting local recurrence, lymphatic/haematogenous involvement in patients with prostate carcinoma.

**Methods**:

1421 patients with prostate cancer were enrolled in our institution between July 2012 and May 2018. Patients were separated in two main groups: biochemical progression 1230 (86%) and 191 (14%) staging. Each group was divided in three subgroups according to PSA levels: ≤1 ng/ml; ≥ 1-5ng/ml and >5 ng/ml. All of them underwent “Dual phase” PET-CT consisting of initial pelvis starting 20 minutes after the injection of 18 F-Choline followed by 1 hour delayed PET of the whole body.

**Results**:

2321 lesions showed increased uptake on FCH-PET and were interpreted as local recurrence or metastases. According to biochemical progression we identified that the majority of patients have PSA levels of >5 ng/ml (713 patients 58%) and the most frequent site of compromised was pelvic lymph nodes(34%), local recurrence(27%) and extrapelvic lymph nodes(23%). On the other hand, for staging patients we have found that most of them have PSA levels of ≤1 ng/ml (105 patients 55%) and we have detected that they most commonly have local compromise (37%), pelvic lymph nodes (32%) and extrapelvic lymph nodes(15%).

**Conclusions**:

In our experience 18 FCH PET/CT is a useful tool to detect lesions in patients with biochemical progression but it seems to have limited value for staging.

#### P22 MRI assessment of lymphadenopathy in patients with cancer of the cervix and HIV co-morbidity

##### Mutala TM¹, Kimatu DM¹, Mugi B², Othieno P², Kosgei RJ¹

###### ^1^University of Nairobi, Nairobi, Kenya; ^2^Kenyatta National Hospital, Nairobi, Kenya

####### **Correspondence:** Mutala TM (mutala@uonbi.ac.ke)

**Aim:**

To assess the pattern of lymph node involvement on MRI in patients with HIV infection and cervical cancer co-morbidity.

**Materials and Methods**:

Eleven patients with established HIV infection were identified from a group of 41 patients that had been referred for pelvic MRI tumour assessment within the Kenyatta National Hospital and Plaza Imaging Centre in Nairobi, Kenya between March 2017 and September 2017. The pattern of lymphadenopathy was assessed using T1W, T2W, DWI and DCE MRI sequences. Size and morphological characteristics were defined as the key descriptors in predicting nodal involvement of the disease. Involved lymph node groups were also described. Data was analysed using SPSS v.20 statistical software and subjected to a Fisher’s exact test for significance.

**Results**:

Patients with HIV demonstrated significantly more nodal disease than non-HIV patients at a p-value 0.0028. The number of nodal groups involved was also higher in the group of patients who had HIV infection.

**Conclusion**:

The results of this small study indicate that more nodal disease is likely to be encountered in the HIV patient than in the non-HIV patient during MRI assessment of cervical cancer. More studies will be required to establish the factors behind our observations.

#### P23 Evaluation of multiparametric MRI (Mpmri) at 3T in prostate cancer in a private outpatient setting

##### M Pixberg^1^, J Ghafur^1^, D Hercher^2^, J Müller - Hübenthal^1^

###### ^1^Praxis im Koelntriangle, Cologne, Germany; ^2^Urologisches Zentrum Refrath, Bergisch Gladbach, Germany

####### **Correspondence:** M Pixberg (martin.pixberg@web.de)

**Aim:**

Assess the performance of mpMRI @ 3T according to PI-RADS 2.0 scoring system.

Find a possible correlation between ADC Value and Gleason Score.

**Materials & Methods:**

Between 1.1.2017 and 31.3.2018 a total of 151 mpMRI examinations of the prostate were taken, Specifications of PI-RADS 2.0 were applied to a Skyra XQ pTx 64 ch (Siemens, Erlangen, Germany). Follow up information was provided by referring doctors for 43 patients on the basis of a questionnaire, patients are yet to be contacted.

**Results:**

Index lesions were classified PIRADS 2 (n=45), 3 (n=21), 4 (n=47) and 5 (n=32).

In PIRADS 3 lesions the median PSA density was 0,085 ng/ml/ml, median ADC Value was 0,98.

PIRADS 4: PSAD 0,114 ng/ml/ml; ADC Value 0,9.

PIRADS 5: PSAD 0,23 ng/ml/ml; ADC Value 0,69.

From 79 lesions classified PIRADS 4 and 5, 16 biopsies were taken, 6 received surgery, 0 received radiation therapy. 4 patients decided against biopsy, 53 pending information.

Gleason score was obtained for 26 index lesions.

Gleason 6 lesions (n=10) had a mean ADC Value of 0,9;

Gleason 7 lesions (n=12) a mean ADC Value 0,68;

Gleason 8 lesions (n=3) a mean ADC Value of 0,89 and

Gleason 9 lesion (n=1) an ADC Value of 0,97.

**Conclusions:**

PSAD is higher and ADC lower in PIRADS 5 vs. PIRADS 3 index lesions. On the basis of limited follow up data, no correlation of ADC Value and Gleason Score could be established. These preliminary data will be supplemented by Ultrasound fusion biopsy data from 2018.

#### P24 3d doppler mapping of cutaneous and subcutaneous lymphoma

##### Robert Bard (rbard@cancerscan.com)

###### Bard Cancer Center, New York, NY, USA

**Aim:**

Demonstrate the accuracy of 3D power Doppler sonography mapping of malignant neovascularity in lymphoma.

**Methods:**

2911 patients with dermal/subdermal pathologies were scanned with a 17 mHz linear probe with 3D/4D doppler image reconstruction over a 3 year period. Vascular index percentage of vessel neovascularity was computed using computed histogram vessel analysis.

**Results:**

6 patients were proven lymphoma. Prominent vascular patterns reflected invasive tumour potential. Dormant lesions had no penetrating vessels. Unsuspected feeding vessels were mapped prior to biopsy and changes in surgical approach occurred in 3/6 patients with connections to major arteries. 2 lesions were not clinically palpable.

**Conclusion:**

3d power Doppler imaging appears to be an accurate guide to tumour aggression. Non-palpable lesions may be detected with this technology. Unsuspected vascular connections may be discovered and appropriate biopsies/treatment performed.

#### P25 Vascular mapping of treated melanoma metastases

##### Robert Bard (rbard@cancerscan.com)

###### Bard Cancer Center, New York, NY, USA

**Aim:**

To compare the accuracy of 3d power doppler sonography (3d-pds) mapping of malignant melanoma lymphadenopathy with pet/ct.

**Methods:**

19 patients with malignant adenopathy were pre and post treatment scanned with a 17 mhz linear array probe with 3d doppler image reconstruction. Vascular index percentage of vessel neovascularity was followed using computed histogram vessel analysis. Patients were simultaneously imaged by dce-mri and/or pet/ct scans.

**Results:**

Vascular patterns reflect invasive tumour potential. Inactive lesions had non penetrating vessels. Aggressive metastases showed vascular index (vi) > 23%. Medium grade tumours had vi between 5-20%. Low grade lesions showed vi < 5%. 9 melanoma metastases were nonpalpable.

**Conclusion:**

3d power doppler imaging appears to be an accurate non-invasive guide to tumour aggression and treatment efficacy as followed by histogram computed vessel density indices. Non palpable lesions may be discovered with this technology.

#### P26 Prostate cancer neovascular response to antioxidants

##### Robert Bard (rbard@cancerscan.com)

###### Bard Cancer Center, New York, NY, USA

**Aim:**

To show response to therapy by 3-D Doppler vessel density imaging.

**Methods:**

111 patients treated with antioxidant supplement therapies composed of beta sitosterol, resveratrol, herbal antioxidants were followed over a four year period with prostate cancer were prospectively scanned with a GE Voluson E-9 unit employing endorectal 18 mhz probe employing conventional 3D/4D imaging using 3d angio and glass body power Doppler image reconstruction. 88 patients had Gleason 3, 23 had Gleason 4. Follow up at 6, 12, 18, 24, 30 and 36 months was obtained. Vessel index was assessed on sonography by 3D histogram analysis and by DCE-MRI. Follow up biopsies were obtained shortly after imaging studies which occurred on a 6 month basis.

**Results:**

Gleason grade 3 (low grade): 72/88 patients had decreased vascular indices indicating positive response to the protocol. PSA lowering was noted. Gleason grade 4 (high grade): 10/23 patients had decreased vascular indices indicated positive response to the protocol. 8 patients (Gleason 3+4 or 4+3) showed disease progression and PSA rise indicating negative response to the protocol. DCE-MRI confirmed all ultrasound tumour vascular findings. Biopsy correlation was good.

**Conclusion:**

Vessel density ultrasound indexing and DCE-MRI analysis correlated well with positive biochemical response to antioxidant therapies. Non responding patients with aggressive tumours were referred for alternative treatments in a timely manner.

#### P27 Breast cancer survivors: Is routine ‘mammograms only’ enough or is it time for personalised follow up?

##### T Telford, G J Bansal

###### The Breast Centre, UHL, Cardiff and vale UHB, Cardiff, UK

####### **Correspondence:** G J Bansal (gjbansal@gmail.com)

**Aim:**

The aim of this study was to evaluate factors affecting local recurrence in ‘margin negative’ breast cancer survivors and their mode of presentation.

**Materials and Methods:**

Between 2011 and 2017, 1800 breast cancer patients were treated in the University hospital. Out of these, 41 patients developed local recurrence and 25 had contralateral breast recurrence. Factors including type of cancer, grade, receptor status, presence of ductal cancer in situ (DCIS), lymphovascular invasion (LVI) in primary cancer, along with mode and time of presentation of recurrence was analysed.

**Results:**

80.5% of local recurrences were symptomatic at the time of presentation, lump being the most common presentation followed by rash and pain. Rest (19.5%) were detected on follow-up mammograms. 56% had breast-conserving surgery. 36% of primary cancers were lymph node positive and received axillary node clearance. 54% received radiotherapy on the side of the local recurrence. The receptor type of primary and recurrent cancer matched in 88% of patients, with Estrogen receptor (ER) positive and Triple negative (TN) being the most common receptors in recurrent cancers. 58.3% of primary cancer had LVI and 78.3% had DCIS. 41% of local recurrent cancers occurred within 5 years and 46% after 10 years**.**

**Conclusion:**

80.5% of locally recurrent cancers present as symptomatic lumps and continue to present after 10 years. This questions the role of current UK annual ‘mammographic only’ screening in breast cancer survivors for 5 years.

#### P28 Assessment of pediatrics neck masses by diffusion MR imaging

##### A. Baiomy^1^, A. Youssef^2^, A. Nada^1^

###### ^1^MD Anderson Cancer Center, Houston, Texas, USA; ^2^National Cancer Institute Cairo University, Cairo, Egypt

####### **Correspondence:** A. Nada (amn_med09@cu.edu.eg)

**Purpose:**

Head and neck masses in children represent a spectrum of malignant tumours and benign lesions that are sometimes difficult to be differentiated by conventional imaging techniques. Our objective was to determine whether diffusion-weighted images (DWI) and calculated apparent diffusion coefficient (ADC) values correlate with the pathologic diagnosis of pediatric head and neck lesions and therefore allows more accurate lesions characterisation.

**Materials and Methods:**

One hundred pediatric patients with recently diagnosed head and neck tumours who underwent preoperative conventional MRI and DWI were included in this study. The average ADC obtained from each tumour was compared with the histological diagnosis of benign, locally malignant or malignant.

**Results:**

We found a significant negative correlation between average ADC and tumour histopathologic diagnosis (P < 0.001, r = *-0.54*): The mean ADC values of the malignant tumours, locally malignant masses and benign lesions were ( 0.83±0.23) ×10^–3^, (1.43±0.17) ×10^–3^ and (1.65±0.58)×10^–3^ mm^2^ s^−1^, respectively . The ADC value of benign and locally malignant lesions are overlapped. Cut off value ≤ 1.19 ×10^–3^ mm^2^ s^−1^ was used for differentiation between benign and malignant pediatric head and neck masses with a sensitivity of 97.3%, specificity of 80.0%, and positive predictive value of 94.7% and negative predictive value of 88.9%.

**Conclusion:**

Diffusion MRI study is a significant, accurate, fast, non-invasive and non-enhanced technique that can be used for characterisation of the head and neck lesions and to differentiate malignant from benign lesions relying on calculated ACD value.

#### P29 Uses of various techniques to minimise the risk of pneumothorax in Image guided thoracic biopsies

##### B K Choudhury

###### Dr B Borooah Cancer Institute, Guwahati, India

####### **Correspondence:** B K Choudhury (choudhury60@gmail.com)

**Aim:**

The purpose of our study was to determine the efficacy of uses of different techniques to minimise the risk of pneumothorax in image-guided thoracic biopsies.

**Methods:**

Needle biopsies were performed in 1890 patients from February, 1989 to December, 2016 using Fluoroscopy, Ultrasound and Computed Tomography ( CT ) as guidance. After reviewing the patient’s data, a plan was formulated and a needle was guided to the lesion for biopsy with an appropriate Image guidance. Several techniques were applied to minimise the risk of pneumothorax and these included immediate dependent positioning of puncture site, extrapleural approach, widening of extrapleural space by saline injection, transsternal approach, pathway through non-aerated lung, minimizing pathway of normal aerated lung, avoiding fissure & bullae, use of small bore needle etc. All patients underwent chest radiography to detect a pneumothorax.

**Results:**

Fluoroscopy, Ultrasound and CT as guidance were used in 170 (8.99%), 325 (17.19%) and 1395 (73.81%) cases respectively. Diameter of masses ranges from 1.0 cm to 12 cm. Results obtained in 1655 cases (87.57%). Malignant cases were 1285 (77.64 %). Complications included pneumothorax in 43 (2.28%) and haemoptysis ( 7 ). There was no pneumothorax in Ultrasound guided biopsy. Pneumothorax occurred in 10 cases following fluoroscopic guidance & 33 cases in CT guidance. Only 3 cases of pneumothorax required placement of a chest tube, rest were small & resolved spontaneously. Incidence of pneumothorax was significantly low because of uses of several techniques.

**Conclusion:**

Most important and common complication of Image-guided thoracic biopsy is pneumothorax which can be significantly minimised by using several techniques.

#### P30 Breast tumours associated with Li-Fraumeni syndrome: review of histopathological spectrum and imaging features

##### Caroline Malhaire^1^, Pia AKL^1^, Marick Lae^2^, Sophie Frank^3^, Marion Gauthier-Villars^4^, Anne Tardivon^1^

###### ^1^Radiology department, Institut Curie, PSL Research University, Paris, France; ^2^Pathology department, Institut Curie, PSL Research University, Paris, France; ^3^Medical Oncology department, Institut Curie, PSL Research University, Paris, France; ^4^Oncogenetic department , Institut Curie, PSL Research University, Paris, France

####### **Correspondence:** Caroline Malhaire (caroline.malhaire@curie.fr)

**Learning Objectives:**

To review the histopathological types and imaging features of breast tumours associated with Li-Fraumeni Syndrome (LFS)

**Content Organisation:**

LFS is a rare autosomal dominant heritable syndrome related to a germline mutation of the tumour suppressor gene TP53 that predispose carrying patients to a broad spectrum of cancers, including breast cancer, sarcoma of soft tissue and bone, brain tumours and adrenocortical carcinoma. Breast cancer are the more frequent tumours associated with LFS. Nonetheless, less is known about the distribution of histopathogical types of breast lesions in LFS.

We will present the review of LFS cases with breast disease identified from the database of our institution (Institut Curie, Paris, France), including mainly breast carcinoma, phyllodes tumour, ductal in situ carcinoma and breast sarcoma.

We will emphasise the radio-histological correlations of the various breast lesion types and present their different management.

We will discuss the current guidelines for imaging screening and surveillance of LFS patients.

**Conclusions:**

Breast tumours are the most frequent tumours associated with LFS with around 30% of patients diagnosed with cancer aged < 30 years. Breast lesions associated with LFS are not limited to invasive carcinoma. It is important for radiologists to be familiar with the wide spectrum of breast tumours in LFS, since LFS patients are exposed to a broad range of cancers occurring at a particularly young age making the screening a major clinical challenge.

#### P31 Role of computed tomography in predicting lesser sac/omental disease in patients with advanced ovarian carcinoma

##### S Mukhopadhyay, S Sen, A Chandra, D Lingegowda, A Chatterjee, P Ghosh, A Mukhopadhyay, R Shrestha

###### Department of Radiology and Gynaecological Oncology, Tata Medical Center, Kolkata, India

####### **Correspondence:** S Mukhopadhyay (sumitmukhopadhyayrad@gmail.com)

**Aims**:

Lesser sac (LS) involvement is reported in 60-70% of patients undergoing cytoreductive surgery (PDS or IDS) for advanced (FIGO III/IV) epithelial ovarian cancer (AEOC) . We aimed to study whether computed tomography (CT) scan can accurately predict the lesser sac/omental disease pre-operatively.

**Methods**:

Retrospective observational study between Jan 2015 to Dec 2017. CT scan of the abdomen performed within 3 weeks of surgery was evaluated by a dedicated radiologist blinded to the operative findings. Clinical data was obtained from the Redcap database and hospital electronic medical records. Intraoperative findings of lesser sac/omentum (LS) involvement were prospectively recorded in a predesigned proforma with laparoscopic evaluation for small volume disease and photographic documentation.

**Results**:

Pre-operative CT and intraoperative details were correlated in 213 patients. 83 (39%) patients had documentation of LS disease in the intraoperative notes. Retrospective review of the CT scans of 3 years (n=213) showed 86% sensitivity, 84% specificity, 82% PPV and 87% NPV in predicting LS lesions. CONCLUSIONS: CT scan is highly accurate in predicting the LS disease in patients with AEOC. However, it requires a learning curve and correlation with intra-operative photographs to build up expertise in recognizing small volume lesions.

### Oral Abstracts

#### O1 CT radiomic features predict microsatellite instability in colorectal cancer

##### Golia Pernicka JS^1^, Gagniere J^2,4^, Chakraborty J^2^, Yamashita R^1^, Nardo L^1^, Creasy JM^2^, Petkovska I^1^, Gonen M^3^, Weiser MR^2^, Simpson AL^2^, Gollub MJ^1^

###### ^1^Departments of ^1^Radiology, ^2^Surgery, ^3^Epidemiology & Biostatistics, Memorial Sloan Kettering Cancer Center, New York, NY, USA; ^4^Department of Digestive and Hepatobiliary Surgery, U1071 INSERM / Clermont-Auvergne University, University Hospital of Clermont-Ferrand, Clermont-Ferrand, France

####### **Correspondence:** Golia Pernicka JS

**Aim:**

Colorectal cancer (CRC) can be classified as either having microsatellite instability (MSI), present in approximately 15% of cases, or as microsatellite stable (MSS). MSI CRC have a slightly better prognosis and are more responsive to immunotherapy than traditional chemotherapy. This study aims to predict MSI status of CRC using quantitative image analysis of preoperative CT.

**Materials and Methods:**

In this IRB-approved retrospective study, patients with resected stage II-III CRC between 2004 and 2012 were included if they had known mismatch repair proteins expression status, determined using immunohistochemistry, and preoperative portal venous phase CT. A radiologist manually segmented the primary CRC using ITK-SNAP software. Computer based quantitative image analysis was performed using MatLab to capture regional variation in enhancement pattern. Imaging features alone or in combination with clinical variables (age, location of tumour) were included in multivariate analysis to predict MSI status and randomly separated into training (80%) and test (20%) sets.

**Results:**

198 stage II-III CRC patients met the inclusion criteria. 134 (68%) patients were MSS compared to 64 (32%) patients with MSI CRC. Quantitative imaging features predicted MSI with an AUC of 0.74 for the training set and 0.72 for the test set. Imaging features combined with clinical variables reached an AUC of 0.77 for the training set and 0.73 for the test set.

**Conclusions:**

This study reveals the potential of quantitative image analysis to predict MSI for primary CRC.

#### O2 MRI findings following papillon contact x-ray brachytherapy for rectal cancer

##### M Dunstan, A Stewart, T Rockall, K Potter

###### Royal Surrey County Hospital, Guildford, UK

####### **Correspondence:** M Dunstan (matthew.dunstan@doctors.org.uk)

Teaching/Discussion points:

Papillon radiotherapy (rectal contact X-ray brachytherapy) may be used as a first line treatment for patients with rectal cancer. It is appropriate for elderly patients for whom surgical resection would carry a high-risk and for patients who will not accept a permanent stoma. If Papillon fails, major curative surgery must be offered. The ability to recognise response, regrowth or recurrence on surveillance MRI is therefore critical.

- Discuss the role of MRI in the surveillance of patients post Papillon radiotherapy (rectal contact X-ray brachytherapy) when used as first line treatment
- Describe the MRI features of response and regrowth or recurrence on surveillance MRI
- Our surveillance protocol

Image – Post treatment surveillance MRI
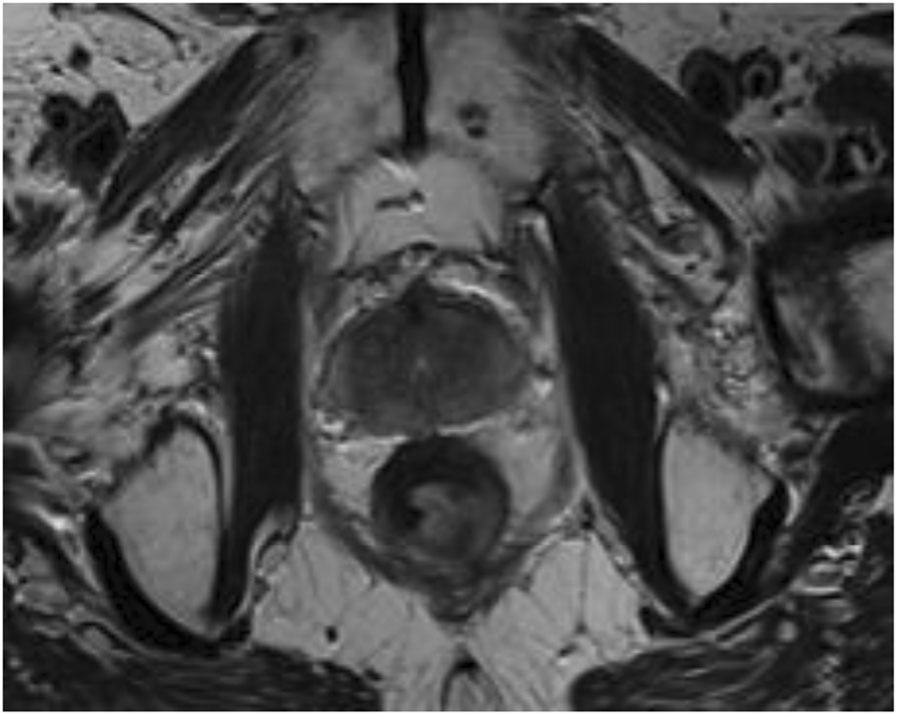


#### O3 Radiogenomics in rectal adenocarcinoma: associations between qualitative and quantitative MRI imaging features and genetic mutations

##### Horvat N, Veeraraghavan H, Pelossof RA, Jorge MCFPT, Arora A, Khan M, Marco M, Cheng C, Gonen M, Gollub MJ, Garcia-Aguillar J, Petkovska I

###### Memorial Sloan Kettering Cancer Center, New York, USA

####### **Correspondence:** Petkovska I (petkovsi@mskcc.org)

**Purpose:**

To investigate associations between genetic mutations and rectal MRI qualitative diagnostic imaging features and quantitative texture features in patients with rectal adenocarcinoma at primary staging.

**Materials and Methods:**

IRB approved this retrospective study with the following inclusion criteria: patients with rectal adenocarcinoma, genome sequencing and availability of pretreatment rectal MRI. Two radiologists reviewed the scans for qualitative imaging features and segmented the volume of the tumour. Consensus clustering was performed using all the computed texture measures (n=34). Consensus clustering used the K-means algorithm with Euclidean distance between the texture measures. The clusters resulting from the algorithm were used to enumerate the prevalence of gene mutations in those clusters.

**Results:**

The final study population was 65 patients with 241 genes sequenced. The five most frequent gene mutations were APC (n=57, 87.7%), TP53 (n=45, 69.2%), KRAS (n=30, 46.1%), FBXW7 (n=11, 16.9%), and PIK3CA (n=13, 20%). There was no significant correlation between qualitative imaging features and genetic mutations after adjusting for multiple comparisons (p>0.05). The mutations in frequently occurring APC and TP53 were most prevalent in the clusters C1 and least prevalent in cluster C3. Similarly, KRAS mutation was most frequent in the C1. In general, mutations were most frequent in cluster C1 when compared to mutations in clusters C3 and C4.

**Conclusion:**

This pilot study showed no significant correlation between qualitative MRI imaging features and genetic mutations after adjustments for multiple comparisons. However, promising correlations were observed among quantitative features and genetic mutations. Further studies with larger sample size are warranted to further evaluate and validate our preliminary data.

#### O4 Diagnostic accuracy of magnetic resonance (MRI) to predict axillary lymph node metastases in patients with breast cancer

##### A Jaipal, G J Bansal (gjbansal@gmail.com)

###### The Breast Centre, UHL, Cardiff and vale UHB, Cardiff, UK

**Aim:**

The aim of this study was to evaluate the diagnostic accuracy of preoperative Breast MRI for axillary lymph node (LN) metastases in two groups of patients with breast cancer. Those who received Neo-adjuvant chemotherapy (NAC) prior to surgery were compared to those which proceeded straight to surgery (non NAC).

**Materials and Methods:**

Between 2011- 2018, 198 patients with primary breast cancer were divided in two groups (NAC and non–NAC). The preoperative MRI results of axilla were compared to postoperative results in both groups. The axillary LN status (number of abnormal LNs, long axis-short axis ratio, loss of fatty hila and cortical thickness) was recorded. SPSS (ver 21) was used for data analysis and two-sided p<0.05 was taken as statistically significant.

**Results:**

There were 112 patients in the non-NAC group and 86 patients in NAC group. Sensitivity of MRI to detect axillary metastases was 80.95% and specificity was 94.51% in the non –NAC group versus 67.74% and 81.13% in the NAC group (*p= 0.001*). There was greater correlation between the number of abnormal nodes on MRI and postoperative results in the non–NAC group (*p<0.001*).

**Conclusions:**

MRI was less sensitive and specific for the assessment of axillary LNs in patients who received NAC prior to surgery compared to those who do not receive NAC. Low sensitivity of MRI in patients who receive NAC underscore the importance of post NAC biopsy of nodes that turn normal on MRI after NAC.

#### O5 Renal events following intravenous iodinated contrast media administration among inpatients admitted with cancer

##### Chaan Ng^1^, Sanjeeva Kalva^2^, Candace Gunnarsson^3^, Michael P Ryan^3^, Erin R Baker^3^, Dustin M Dunham^4^, Novena A Rangwala^4^, Ravindra Mehta^5^

###### ^1^MD Anderson Cancer Center, Houston, TX, USA; ^2^University of Texas Southwestern Medical Center, Dallas, TX, USA; ^3^CTI Clinical Trial & Consulting Services, Covington, KY, USA; ^4^GE Healthcare, Marlborough, MA, USA; ^5^University of California San Diego UCSD Medical Center, La Jolla, CA, USA

####### **Correspondence:** Candace Gunnarsson (cgunnarsson@ctifacts.com)

**Aim:**

Little evidence exists examining risk of renal adverse events (AEs) in patients with cancer and chronic kidney disease (CKD) undergoing imaging procedures with iodinated contrast media (CM). The objective of this study was to use real-world data to assess whether oncology inpatients undergoing CT or CT angiography (CTA) with CM have higher rates of acute renal AEs than those without cancer.

**Materials and Methods:**

Inpatient visits from the Premier Hospital Database from January 2010 through September 2015 were eligible for inclusion. The outcome of interest was a composite of acute kidney injury, acute renal failure requiring dialysis, contrast-induced AKI, and renal failure. Multivariable models adjusted for patient demographics and comorbidities were developed to assess incremental risk of acute renal AEs by CT/CTA with or without iodinated CM, CKD stage, and cancer subtype.

**Results:**

Inpatient visits (N=29,850,475) across 611 hospitals were analysed. The baseline risk for acute renal AEs in patients without cancer or CKD and no CT/CTA or CM was 0.5%. The absolute risk increases from baseline by 0.2% with CT/CTA and additional 0.8% with iodinated CM. Patients with CKD undergoing CT/CTA with CM have absolute risk of 4.1%-9.7%, increasing with CKD severity. For cancer patients, absolute risk further increases, varying from 0.3%-2.3% depending upon cancer subtype.

**Conclusions:**

Hospitalised oncology patients are at higher risk of acute renal AEs following contrast-enhanced CT compared to those without cancer. Understanding the complex bidirectional relationship between cancer and kidney function and application of mitigation strategies is an important consideration for oncology patients.

#### O6 The use of imaging as a predictor of malignant solitary fibrous tumours of the pleura

##### Agarwal P, Gaikstas G (p.agarwal@doctors.org.uk)

###### Pennine Acute Hospital, Manchester, UK

**Teaching/Discussion points:**

- Highlight the difficulties in diagnosing malignant solitary fibrous tumour of the pleura.
- Describe common features of malignancy seen in patients with solitary fibrous tumours of the pleura in MRI, CT and PET/CT scans.
- Discuss the need for multimodal imaging in diagnosing malignant solitary fibrous tumour of the pleura

Written informed consent was obtained from the participant.
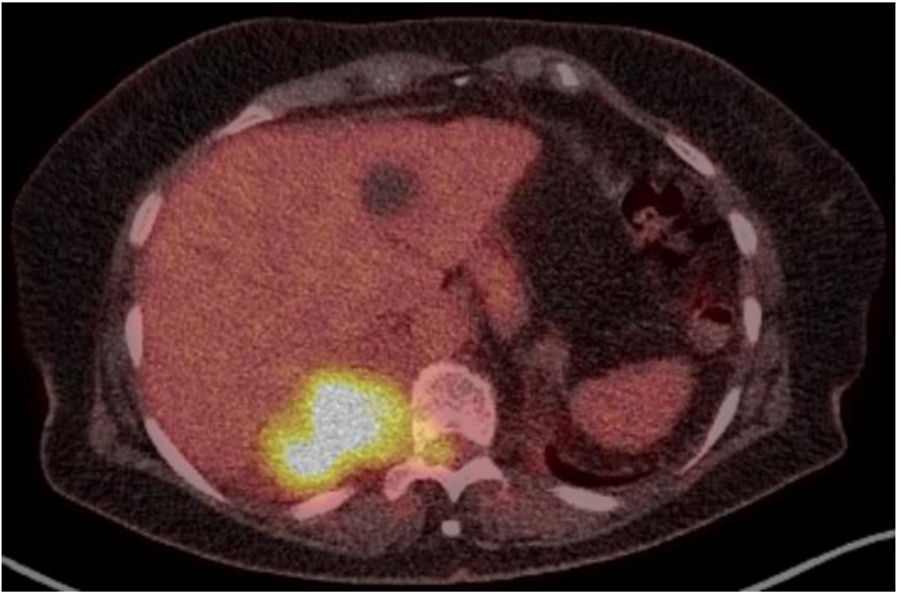


#### O7 Salivary gland tumours: can adding diffusion-weighted imaging to conventional MRI improve accuracy in characterised?

##### D Hady, A Nada, A Youssef, R Assad

###### National Cancer Institute Cairo University, MD Anderson Cancer Center, National Cancer Institute Cairo University, Faculty of Medicine Cairo University, Cairo, Egypt

####### **Correspondence:** A Nada (amn_med09@cu.edu.eg)

**Objective:**

Management plan in treatment of salivary gland tumours varies according to pathological type. Thus, evolving the imaging role to correctly predict the benign or malignant nature of the lesion is of the essence. We aim to investigate the value of adding Diffusion weighted images (DWI) to the conventional MRI in prediction of the salivary gland tumours nature and enhancing the capacity of preoperative prediction of benign and malignant ones.

**Materials and Methods:**

A retrospective study of 51 patients (27 females and 24 males, mean age of 37.2±22.6 years) with salivary gland tumours; Conventional MRI in addition to DWI with calculated apparent diffusion coefficient (ADC) value were reviewed for all patients. The results were correlated with histopathological findings.

**Results:**

There were 16 benign and 35 malignant lesions as detected by histopathological diagnosis. There is statistically significant difference between the ADC for benign and malignant lesions (1.39 ± 0.52x ^10-3^mm^2^/s and 0.69 ± 0.22x 10^-3^mm^2^/s) respectively.)

The optimal cutoff ADC value was determined for discrimination of these lesions is 1.075 with 97.14 % sensitivity and 75 % specificity.

Using Conventional MRI alone in predicting benign and malignant lesions has the sensitivity and specificity of 64.7 % and 43.7 % respectively.

After combining DWI, the accuracy has increased, as the sensitivity and specificity were 100%, 87.5% respectively with 94.4% positive predictive value and 100% negative predicative value.

**Conclusion:**

Adding DWI to the conventional MRI can provide better assessment of salivary gland tumours and predict the benign and malignant lesions.

#### O8 Can MRI radiomics predict the type of histone H3 mutations in diffuse intrinsic pontine glioma?

##### Jessica Goya-Outi^1*^, Fanny Orlhac^2^, Raphael Calmon^3^, Vincent Frouin^4^, Cathy Philippe^4^, Nathalie Boddaert^3^, Stéphanie Puget^3^, Jacques Grill^5^, Irène Buvat^1^, Frédérique Frouin^1^

###### ^1^IMIV, Inserm, CEA, CNRS, Univ. Paris-Sud, Université Paris Saclay, Orsay, France; ^2^Epione, INRIA Sophia-Antiopolis, Valbonne, France; ^3^Hôpital Necker Enfants Malades, Paris, France; ^4^UNATI, Neurospin, CEA, Université Paris-Saclay, Gif-sur-Yvette, France; ^5^Gustave Roussy; CNRS UMR 8203, Université Paris Saclay, Villejuif, France

####### **Correspondence:** Jessica Goya-Outi (jessicaouti@gmail.com)

**Aim:**

Diffuse intrinsic pontine glioma (DIPG) is a rare pediatric cancer with poor prognosis. Diagnosis is made based on typical MRI appearance but histone H3 mutations (H3.3K27M, H3.1K27M) in the tumour are associated with patient overall survival. Our aim was to study whether MRI radiomics could predict these mutations.

**Methods:**

Diagnostic MRI, including T1-weighted, post-contrast T1-weighted, T2-weighted, and FLAIR scans of 31 patients (9 H3.1K27M, 22 H3.3K27M) were analyzed. MRI intensities were standardised and images resampled to 1 mm^3^ voxels. For each patient, the largest spherical region inside the tumour was defined and a 5 mm radius sphere was used to scan this region. By computing 79 radiomic features inside each 5 mm sphere location, 79 feature maps were estimated per image (316 per patient). The mean values within the maps were used for the classification task. Features were selected according to their robustness to sphere delineations, and to their area under the ROC curve. Sets of highly correlated features were represented by the most discriminative feature. Random Forest classifiers were built using: conventional clinical criteria (sex, age, tumour volume) (RF1), selected image features (RF2), clinical criteria and selected image features (RF3). F1-weighted scores in Leave-One-Out Cross-Validation were used to characterise the classifiers’ performance.

**Results:**

F1-weighted scores were 0.686, 0.714, and 0.836 for RF1, RF2, and RF3, respectively. For RF3, the H3.3K27M sensitivity and specificity were 91% and 67%.

**Conclusions**:

Combination of clinical and MRI radiomic features can help to discriminate the type of histone H3 mutation in DIPG non-invasively.

#### **O9 Diffusion-weighted imaging can differentiate pleural dissemination from malignant pleural mesothelioma emphema, or pleural effusion**

##### K. Usuda^1^, S. Iwai^1^, A. Funasaki^1^, A. Sekimura^1^, N. Motono^1^, M. Matoba^2^, M. Doai^2^, S. Yamada^3^, Y. Ueda^4^, H. Uramoto^1^

###### ^1^Department of Thoracic Surgery, Kanazawa Medical University, Kahoku District, Japan; ^2^Department of Radiology, Kanazawa Medical University, Kahoku District, Japan; ^3^Department of Pathology and Laboratory Medicine, Kanazawa Medical University, Kahoku District, Japan; 4 Department of Pathophysiological and Experimental Pathology, Kanazawa Medical University, Kahoku District, Japan

####### **Correspondence:** K. Usuda (usuda@kanazawa-med.ac.jp)

**Aim:**

Although DWI is useful for the assessment of lung cancer and mediastinal tumour, it is not clear whether DWI is useful for the assessment of pleural lesions. The aim of this study is to determine whether DWI can differentiate pleural dissemination of lung cancer from malignant pleural mesothelioma (MPM), empyema or pleural thickness, or pleural effusion.

**Material & methods:**

DWI using a single-shot echo-planar method were performed with respiratory triggered scan. b value was set as 0 and 800 s/mm2. The optimal cutoff value of ADC for diagnosing malignancy in DWI was determined to be 1.70×10-3mm2/sec as previously reported. There were 6 pleural disseminations, 11 MPMs, 7 empyemas or benign pleural thicknesses, and 5 pleural effusions. All the cases were evaluated by surgery or pathological examination.

**Results**:

ADC values of pleural lesions were 1.271±0.32 ×10-3mm2/sec in MPM, 1.38±0.43 ×10-3mm2/sec in pleural dissemination, 2.08±0.23 ×10-3mm2/sec in empyemas or benign pleural thicknesses and 3.54±10-3mm2/sec in pleural effusions. ADC values of the malignant pleural lesions were significantly lower than those of benign pleural lesions. For diffusion pattern of DWI, 5 of 6 pleural disseminations showed scattered & strong diffusion and 1 pleural dissemination diffused & strong diffusion; all the 11 MPMs showed diffused & strong diffusion; all of empyemas or benign pleural thicknesses showed diffused & moderate diffusion; none of pleural effusions showed any diffusion.

**Conclusions:**

DWI can qualitatively evaluate pleural lesions and differentiate pleural dissemination of lung cancer from MPM, emphema, benign pleural thicknesses or pleural effusion.

#### O10 Generating evidence for clinical benefit of PET/CT: results of the first oncologic PET/CT registry in Germany

##### Autoren: Pfannenberg C^1^, Gückel B^1^, Gatidis S^1^, Olthof SC^1^, Reimold M ^2^, la Fougere C^2^, Nikolaou K^1^, Martus P^3^

###### ^1^Department of Diagnostic and Interventional Radiology, University of Tuebingen, Tuebingen, Germany; ^2^Department of Nuclear Medicine, University of Tuebingen, Tuebingen, Germany; ^3^Institute of Clinical Epidemiology and applied Biostatistics, University of Tuebingen, Tuebingen, Germany

####### **Correspondence:** Pfannenberg C (christina.pfannenberg@med.uni-tuebingen.de)

**Aim:**

To evaluate the impact of PET/CT on clinical management based on a prospective data registry. The registry was developed to meet the requirements for PET/CT coverage by public health insurance.

**Methods:**

We evaluated a prospective patient cohort having a clinically indicated PET/CT at one German University Center from 4/2013 to 8/2016. The registry collected questionnaire data from requesting physicians on intended patient management before and after PET/CT. 4,504 patients with 5,939 PET/CT examinations were enrolled in the registry, including evaluable data from 3,724 patients receiving 4,754 scans. The impact of PET/CT was assessed across 22 cancers types, for different indications and categories of treatment and non-treatment (watching, additional imaging, biopsy).

**Results:**

The most frequent PET/CT indication was cancer staging (59.7%). Melanoma, lung and prostate cancer, lymphoma and NET accounted for 70% of cases. Overall, PET/CT was associated with a 37.1% change in clinical management ( 95% CI, 35.7-38.5), most frequently (30.6%) from nontreatment to treatment. The frequencies of changes ranged from 28.3% for head and neck cancers up to 46.0% for melanomas. The impact of PET/CT was greatest in reducing demands for additional imaging which decreased from 66.1% to 6.1%. Pre-PET/CT planned invasive tests could be avoided in 72.7% of cases. In case the intended management was treatment, the treatment goal (curative or palliative) changed after PET/CT in 21.7% of cases.

**Conclusions:**

The data of this large prospective registry confirmed that physicians often change their intended management on the basis of PET/CT consistent across different cancer types and indications.

#### O11 Management impact of 68Ga-THP-PSMA in high-risk and biochemically recurrent prostate cancer

##### Cook GJR^1^, Hughes S^2^, Morris S^2^, Challacombe B^2^, Cathcart P^2^, Popert R^2^, Brown C^2^, Dasgupta P^1^, John J^1^, Mallia A^1^, Young J^1^, Gibson V^2^, Warbey V^1^

###### ^1^King’s College London, London, UK; ^2^Guy’s & St Thomas’ NHSFT, London, UK

####### **Correspondence:** Cook GJR (gary.cook@kcl.ac.uk)

**Aim:**

To determine the impact on clinical management of newly diagnosed patients with high-risk prostate cancer and patients with biochemical recurrence (BCR) using 68Ga-THP-PSMA PET-CT, a new kit form of PSMA developed for clinical imaging.

**Methods**:

118 consecutive patients (50 pre radical treatment, 68 BCR) had management plans documented at the MDM before 68Ga-THP-PSMA PET-CT. All patients underwent 10min dynamic pelvis, followed by 60 min half body, acquisitions after injection of 102-182 (mean 159) MBq 68Ga-THP-PSMA. Post scan management plans were then recorded after MDM discussion or clinical review. Gleason score, PSA and PSA doubling time (dt) were also recorded.

**Results**:

High-risk pre-radical treatment: 12/50 (24%) patients prior to first radical treatment (6 to a different treatment modality, 6 intramodality) had management changed. Gleason scores ≥8 were associated with detection of more nodal (25% vs 12.5%) or bone (12.5% vs 6.5%) metastases.

BCR: 40/68 (59%) of scans were positive. Positivity rate increased with PSA level (PSA 0.5-1.0 ng/ml 35%, PSA 5.0-10.0 ng/ml 91%), PSA dt of < 6 months (58.3% vs 45.7%) and Gleason score ≥8 (78.9% vs 51.2%). Positive scans were associated with higher PSA levels (0.82-71.0, mean 6.23ng/ml vs 0.11-14.2, mean 1.74ng/ml). Clinical management changed in 23/68 (34%) patients (15 intermodality, 8 intramodality).

**Conclusion**:

68Ga-THP-PSMA PET-CT influences clinical management compared to standard workup in significant numbers of patient with high-risk prostate cancer pre radical treatment and in those with biochemical recurrence.

#### O12 Quantified PET/CT parameters can predict non-small cell lung cancer pathologic characteristics

##### A Doroudinia^1^, M Bakhshayesh Karam^2^, B Behzadi^3^

###### ^1^Chronic Respiratory Diseases Research Center, Tehran, Iran; ^2^National Research Institute of Tuberculosis and Lung Diseases (NRITLD), Tehran, Iran; ^3^Shahid Beheshti University of Medical Sciences, Tehran, Iran

####### **Correspondence:** A Doroudinia (abtin1354@gmail.com)

**Aim:**

Relationship between maximum standardised uptake value (SUV_max_) with tumour size and tumour pathological characteristics can suggest equations between SUV_max_ and tumour size in patients with non-small cell lung cancer (NSCLC) which can help differentiate pathology types.

**Material and Methods:**

We retrospectively analysed the FDG-PET/CT findings in 98 patients with NSCLC. Statistical differences were considered significant when P<0.05. Correlation between SUV_max_ and other variables were determined by Pearson and Spearman correlation. Both linear and nonlinear regression analysis were used to determine equations between SUV_max_ and tumour size to help differentiate between pathology types.

**Results:**

The mean SUV_max_ in patients with squamous cell carcinoma was significantly higher than those with adenocarcinoma (21.35±1.73 versus 13.75±0.89, p=0.000). The results of regression analysis indicated that among all equations determined with relative accuracy the "cubic equation" has the highest accuracy when considering the relationship between SUV_max_ and tumour size in patients with adenocarcinoma. In patients with squamous cell carcinoma, the most accurate equation was obtained using the "quadratic equation".

**Conclusion:**

There was significant correlation between SUV_max_ and tumour differentiation and tumour size in patients with adenocarcinoma. SUV_max_ of patients with squamous cell carcinoma had also significant correlation with tumour size. Overall SUV_max_ of patients with NSCLC could be predicted by tumour size value. In patients with squamous cell carcinoma compared to those with adenocarcinoma, SUV_max_ with less accuracy can be determined by tumour size. Linear regression analysis line slope can be used as an index for distinguishing adenocarcinoma from squamous cell carcinoma.

